# Entropy of human leukocyte antigen and killer-cell immunoglobulin-like receptor systems in immune-mediated disorders: A pilot study on multiple sclerosis

**DOI:** 10.1371/journal.pone.0226615

**Published:** 2019-12-17

**Authors:** Maurizio Melis, Roberto Littera, Eleonora Cocco, Jessica Frau, Sara Lai, Elena Congeddu, Paola Ragatzu, Maria Serra, Valentina Loi, Roberta Maddi, Roberta Pitzalis, Sandro Orrù, Luchino Chessa, Andrea Perra, Carlo Carcassi

**Affiliations:** 1 Medical Genetics, Department of Medical Sciences and Public Health, University of Cagliari, Cagliari, Italy; 2 Complex Structure of Medical Genetics, R. Binaghi Hospital, ASSL Cagliari, ATS Sardegna, Italy; 3 Multiple Sclerosis Center, R. Binaghi Hospital, University of Cagliari/ATS Sardegna, Cagliari, Italy; 4 Center for the Study of Liver Diseases, Department of Medical Sciences and Public Health, University of Cagliari, Cagliari, Italy; 5 Unit of Oncology and Molecular Pathology, Department of Biomedical Sciences, University of Cagliari, Cagliari, Italy; Centro Cardiologico Monzino, ITALY

## Abstract

**Background:**

Entropy is a thermodynamic variable statistically correlated with the disorder of a system. The hypothesis that entropy can be used to identify potentially unhealthy conditions was first suggested by Schrödinger, one of the founding fathers of quantum mechanics. Shannon later defined entropy as the quantity of information stored in a system. Shannon’s entropy has the advantage of being adaptable across a variety of disciplines, including genetic studies on complex immunogenetic systems such as the human leukocyte antigen (HLA) and killer-cell immunoglobulin-like receptor (KIR) systems.

**Methods:**

In our study, entropy associated to the HLA and KIR systems was compared between a cohort of 619 Sardinian healthy controls and a group of 270 patients affected by multiple sclerosis (MS), the latter stratified into 81 patients with primary progressive multiple sclerosis (PPMS) and 189 patients with relapsing remitting multiple sclerosis (RRMS).

**Results:**

The entropy associated to HLA four-loci haplotypes (A, B, C, DR) and combinations of two inhibitory KIR genes was significantly higher in patients affected by RRMS than in healthy controls. No significant differences were observed for patients with PPMS. By calculating the total HLA and KIR entropy ratio in each subject, it was possible to determine the individual risk of developing MS, particularly RRMS.

**Conclusions:**

In addition to the standard statistical methods used to evaluate immunogenetic parameters associated to immune-mediated disease, the analysis of entropy measures the global disorder status deriving from these parameters. This innovative approach may represent a useful complementary tool to the risk assessment of immune-mediated disorders. Improved risk assessment is particularly important for family members of patients with MS. However, further investigation is warranted to confirm our findings and to evaluate the validity of the entropy-based method in other types of immune-mediated disorders.

## Introduction

Entropy is a thermodynamic variable related to temperature and heat. According to Boltzmann’s equation, entropy can be interpreted as a measure of the disorder of a system, including living organisms, as discussed by Schrödinger in 1944 in his renowned essay “What is life?” [1]. This interpretation of entropy is in accordance with the one introduced by Shannon in his theory of communication in 1948, when he defined entropy as a measure of the information stored in a system [2].

These different interpretations of entropy are equivalent and the choice to apply one or the other simply depends on the issue under investigation.

The main difficulty encountered when applying entropy in medical research is the development of a method capable of computing the entropy associated to patient conditions. The definition of entropy introduced by Shannon can be adapted to fit the study of complex genetic systems, such as human leukocyte antigens (HLA) and killer-cell immunoglobulin-like receptors (KIR), and their impact on immune response mechanisms.

Human leukocyte antigens of the major histocompatibility complex are well known for their crucial role in immune response mechanisms. The HLA system has specific characteristics in each population. For instance, in Sardinia (Italy) the HLA four-loci haplotypes with the highest frequencies are HLA-A*30, B*18, C*05, DR*03 and HLA-A*02, B*58, C*07, DR*16 [3]. Instead, in other populations the predominant four-loci haplotypes may be different. For this reason, it is possible that association of specific genes or haplotypes with a disease may be found in some populations, but not in others. HLA class I molecules interact with KIRs, a class of receptors that are expressed on the surface of several cells implicated in immune regulation. Among these, natural killer (NK) cells are the most important.

NK cells are a heterogeneous subpopulation of lymphocytes that have a central role in innate immunity and strongly contribute to the regulation of adaptive immune responses [4].

NK cell recognition of target cells depends upon the delicate balance between multiple signals delivered by both activating (*KIR2DS1*, *KIR2DS2*, *KIR2DS3*, *KIR2DS4*, *KIR2DS5*, *KIR3DS1)* and inhibitory (*KIR2DL1*, *KIR2DL2*, *KIR2DL3*, *KIR2DL4*, *KIR2DL5*, *KIR3DL1*, *KIR3DL2*, *KIR3DL3*) receptors. KIRs expressed on the NK cell surface have an important role among the regulators of NK cell activity [5, 6]. Interactions between HLA class I molecules and these receptors are implicated in several human pathologies such as autoimmunity, reproductive failure, viral infections and cancer [7, 8, 9, 10, 11].

HLA and KIR entropy can be used to measure the disorder of the HLA and KIR systems which may be linked to the risk of a subject to develop an immune-mediated disease: the condition with the highest entropy value should be associated with the highest risk of the disease.

Among immune-mediated diseases, multiple sclerosis (MS) is one of the most disabling, as it may cause severe neurological disabilities in young adults. Its diagnosis relies on well established guidelines, but the identification of genetic and environmental factors that influence the risk of development or progression of the disease remains a challenge [12, 13, 14].

MS is an autoimmune disorder in which abnormal immune-mediated responses attack the myelin coating around nerve fibres in the central nervous system and the nerve fibres themselves [12]. Based on the clinical picture, disease course and etiopathogenesis, two main forms of MS have been distinguished: primary progressive multiple sclerosis (PPMS) and relapsing remitting multiple sclerosis (RRMS).

The aim of this study was to develop a test based on HLA and KIR entropy capable of individuating the risk of developing MS. Standard statistical methods were used to analyze the HLA and KIR systems in MS patients and controls. The differences found for these immunogenetic parameters were used to construct an entropy-based test for the risk of MS.

## Materials and methods

We investigated a cohort of 270 MS patients, stratified into 189 patients with RRMS and 81 patients with PPMS. All patients were unrelated and referred to the Sardinian Regional Government Center for Diagnosis and Treatment of MS. The inclusion criteria were as follows: diagnosis of MS according to revised McDonald criteria [15], clinical course of relapsing remitting or primary progressive MS [16], age older than 18 years. Patients who consented to the collection of their blood sample for DNA analysis were randomly recruited after signing written informed consent for participation in the study. Patients diagnosed with a clinically isolated syndrome or other diseases of the central nervous system were excluded.

The following data were collected for each patient: year of onset of MS, disease duration at the last follow-up, level of disability at the last follow-up according to the Expanded Disability Status Scale (EDSS) [17] and progression index (PI), calculated as EDSS/disease duration [18].

A group of 619 unrelated healthy controls were recruited from the Sardinian Voluntary Bone Marrow Donor Registry. In order to minimize the probability of autoimmune disease, age at enrolment was ≥ 50 years.

### HLA and KIR genotyping

HLA and KIR genotyping was performed on genomic DNA extracted from peripheral blood mononuclear cells according to standard methods. Patients and controls were typed at high resolution for the alleles at the HLA-A, -B, -C and DR loci using a polymerase chain reaction sequence-specific primer (PCR-SSP) method according to the manufacturer’s instructions (Allele-specific PCR-SSP kits: Olerup SSP AB, Stockholm, Sweden).

Genomic DNA from patients and controls was typed for the presence of the 14 KIR genes *KIR2DL1*, *KIR2DL2*, *KIR2DL3*, *KIR2DL4*, *KIR2DL5*, *KIR3DL1*, *KIR3DL2*, *KIR3DL3*, *KIR2DS1*, *KIR2DS2*, *KIR2DS3*, *KIR2DS4*, *KIR2DS5* and *KIR3DS1*, using PCR with primers specific for each locus according to a previously reported method [19, 20].

HLA and KIR typing validation was performed by Next Generation Sequencing on 96 randomly selected samples of patients and controls, according to a previously reported method [21].

The concordance of the two different methods was 100%.

The frequencies of the HLA alleles and haplotypes of controls and patients are listed in **S1 Table** while the frequencies of KIR genes and haplotypes are reported in **S2 Table**.

### Shannon’s entropy

If we have a set of possible events whose probabilities of occurrence are *f*_1_, *f*_2_,…,*f*_*n*_, then Shannon’s entropy, measuring the amount of uncertainty associated with the outcome, is given by [2]:

$$S=-k\sum_{i=1}^{n}f_{i}\;\log\;f_{i},$$

where *k* is a positive constant depending on the choice of the units of measurement. In all our calculations we set *k* = 100.

In the case of two possible events, with probabilities *f* (with 0≤*f*≤1) and *q* = 1−*f*, Shannon’s entropy becomes [2]:

S=−k(flogf+qlogq).

The entropy *S* has a maximum for $f=\frac{1}{2}$, i.e. when the probability *f* that the event occurs equals the probability *q* that it does not occur. *S* vanishes only if *f* is one or zero, i.e. only when we are certain of the outcome; in all other cases, the entropy *S* is strictly positive.

The Shannon’s entropy *S* associated to the HLA haplotypes or combinations of two KIR genes (KIR gene couples) of a subject was obtained using the following equation (**S1 File**):

$$S=-\frac{k}{N}\sum_{i=1}^{N}[f_{i}\;\log\;f_{i}{+(1-f}_{i})\log\left(1-f_{i}\right)],$$

where *N* is the number of HLA haplotypes or KIR gene couples for each subject and *f*_1_, *f*_2_,…,*f*_*n*_, are the frequencies of the *n* distinct HLA haplotypes or KIR gene couples observed in the controls (**S3 Table**).

In the **S1 File** we discuss the theoretical framework of the method and provide a detailed example of how to evaluate the entropy of a subject starting from his/her HLA and KIR genotype.

### HLA and KIR entropy

The probabilities of the HLA four-loci haplotypes were computed by considering the combinations of the HLA-A, -B, -C, and–DR alleles in the group of 619 controls. In each subject, both HLA-A alleles were coupled to each of the two HLA-B alleles, which in turn were coupled to both HLA-C alleles and these to each of the two HLA-DR alleles. Hence, for each control there were 2^4^ = 16 possible HLA four-loci haplotypes. The HLA haplotype probabilities were obtained by dividing the number of times each HLA four-loci haplotype occurred in the control cohort by the total number of HLA four-loci haplotypes (**S3 Table**).

The HLA entropy was evaluated by considering the sixteen HLA-A, -B, -C, -DR haplotypes which occurred in each subject. Therefore, we set *N* = 16 in Shannon’s entropy equation (**S1 File**).

In the analysis of the KIR system we excluded the KIR genes with frequencies greater than 90%, i.e. *KIR2DL1*, *KIR2DL4*, *KIR3DL2*, *KIR3DL3*. Subsequently, a logistic regression model [22] was applied to control for confounding variables and select, among the remaining set of ten KIR genes (*KIR2DL2*, *KIR2DL3*, *KIR2DL5*, *KIR3DL1*, *KIR2DS1*, *KIR2DS2*, *KIR2DS3*, *KIR2DS4*, *KIR2DS5*, *KIR3DS1)*, the ones that determined significant differences between the KIR entropy of MS patients and controls, i.e. the following four inhibitory KIR genes: *KIR2DL2*, *KIR2DL3*, *KIR2DL5*, *KIR3DL1*. For each subject, we only considered KIR gene combinations composed of two KIR genes from the previous set of four inhibitory KIR genes. This made it possible to maximize the number of combinations, which turned out to be $\left(\begin{array}{c} 4\\ 2 \end{array}\right)=6$. Each of these KIR gene couples can occur in four different ways: (1, 1), (1, 0), (0, 1) and (0, 0). Therefore, the total number of possible KIR gene couples was 4×6 = 24.

The probabilities of KIR gene couples were obtained by dividing the number of times each couple of specific inhibitory KIR genes occurred in the cohort of controls by the total number of possible KIR gene couples (**S3 Table**).

The KIR entropy was evaluated by considering, in each subject, the six possible couples of KIR genes from a set of four inhibitory KIR genes. Hence, we set *N* = 6 in Shannon’s entropy equation (**S1 File**).

The HLA and KIR entropy in each group of patients (189 subjects affected by RRMS and 81 patients with PPMS) was compared to the HLA and KIR entropy of a cohort of 619 Sardinian healthy controls.

The *absolute* entropy value *S*_*HLA*_ or *S*_*KIR*_ depends on the system under investigation and the chosen reference level. The aim of calculating entropy is to achieve a comparison between different systems (e.g. controls and patients) and so only the relative entropy value is important. For this reason, it seemed appropriate to consider the *ratio R*_*HLA*_ or *R*_*KIR*_ between the HLA or KIR entropy in each group of patients and the mean HLA or KIR entropy in the group of controls.

Moreover, we analyzed the total entropy of the HLA and KIR systems by computing the total entropy ratio *R*_*tot*_, which is given by the mean of the corresponding HLA and KIR entropy ratios:

$$R_{tot}=\frac{1}{2}(R_{HLA}+R_{KIR}).$$

The frequencies and entropies of all HLA four-loci haplotypes and couples of inhibitory KIR genes of the control group are listed in **S3 Table**, which thus allows us to evaluate the total entropy of a single subject according to his/her HLA and KIR genotype.

### Construction of a risk test for MS based on HLA and KIR entropy

In order to evaluate the application of the entropy-based method in clinical practice, we investigated the risk of MS in subjects whose HLA-KIR profile was known. Based on the total entropy ratio *R*_*tot*_ of RRMS patients and controls (see the subheading “Total entropy” in the Results section), it is possible to distinguish three entropy intervals corresponding to three degrees of risk (low, medium and high) for developing MS. To maximize accuracy of risk assessment, we used the 95% CIs of *R*_*tot*_ for controls and RRMS patients to define the cutoff values since the cohort of MS patients had a 95% CI of *R*_*tot*_ that presented a slight overlap with that of the control population.

As discussed in detail in the **S2 File**, the entropy ratio interval corresponding to a *low risk* degree ranged from zero to the upper endpoint of the 95% CI of the total entropy ratio of the controls [control *R*_*tot*_ (95% CI) **=** 1.00 (0.95–**1.05**); *R*_*tot*_<1.05 (low risk)]; the entropy ratio interval corresponding to a *high risk* degree ranged from the lower endpoint of the 95% CI of the total entropy ratio for patients affected by RRMS up to the maximum entropy [RRMS *R*_*tot*_ (95% CI) = 1.23 (**1.10**–1.35); *R*_*tot*_>1.10 (high risk)]; the entropy ratio interval corresponding to a *medium risk* degree ranged between the cutoff levels established for high and low risk degree (1.05≤*R*_*tot*_≤1.10).

Starting from the HLA and KIR genotype of a subject, it is possible to calculate the total entropy and corresponding entropy ratio *R*_*tot*_. Based on the value obtained for the total entropy ratio, the tested subject can be assigned to one of the three categories of risk for developing MS.

An analogous procedure can be separately exploited for the HLA or KIR entropy ratio, if only one of the two genotypes is available.

### Statistical analysis

All statistical computations were performed using R software version 3.5.2 [23]. In particular, the frequencies of the HLA haplotypes, HLA alleles, KIR genes and KIR gene combinations (**S1**, **S2** and **S3 Tables**) were obtained analytically using a programming code created with R language.

**S4 Table** shows the results obtained in our study for HLA linkage disequilibrium (LD). No statistically significant differences for LD were observed between the two groups of patients and controls.

Sample size calculations confirmed the ability of this study to detect minimum significant differences when comparing HLA or KIR entropies in our cohorts of patients and controls. Moreover, the case:control ratio reflected a high power of the study in relationship to the size of the cohorts (more details on sample size evaluation are given in the **S3 File**).

The data on Shannon’s entropy did not follow a normal distribution (as confirmed by the Shapiro-Wilk test) and the variances of the HLA and total entropy in the control group were much lower than the variances in the patient groups (as shown by the F test). However, our cohorts of patients and controls were large enough (more than 50 observations in each compared group) to justify the use of Student’s t distribution (**S4 File**).

Cochran’s rule (**S5 File**) was used to establish which HLA haplotypes and KIR gene combinations had frequencies that were sufficiently high to allow for significant comparisons between the two groups of patients and controls.

The 95% confidence intervals for continuous variables were calculated using Student’s t distribution. P values for comparison between patients and controls were obtained by Student’s t test. Only P values below 0.05 were considered to be statistically significant.

For categorical variables, comparisons between patients and controls were performed using the two-tailed Fisher’s exact test which provided both P values and odds ratios (ORs) with the corresponding 95% confidence intervals.

### Ethical statement

Written informed consent was obtained from each patient included in the study in accordance with the institutional and national ethical standards of the local human research committee. The study protocol, including informed consent procedures, conforms to the ethical guidelines of the Declaration of Helsinki and was approved by the responsible ethics committee (Ethics Committee of the Cagliari University Hospital; date of approval: January, 23, 2014; protocol number NP/2014/456). Records of written informed consent are kept on file and are included in the clinical record of each patient.

## Results

Our cohort of 270 MS patients was stratified into 189 patients with RRMS and 81 patients with PPMS. Basic characteristics of the two groups of patients are shown in **Table 1**. Most patients with RRMS were female (73.5%) while PPMS patients were more often male (54.3%). Subjects with RRMS were younger than those with PPMS [mean ± standard deviation (SD): 46.4 ± 10.3 vs 58.1 ± 10.9 years] and had a lower age at diagnosis in comparison to patients with PPMS (mean ± SD: 28.8 ± 8.7 vs 37.6 ± 10.4 years). The last expanded disability status scale (EDSS) was considerably smaller in the RRMS group compared to the PPMS group (mean ± SD: 2.21 ± 1.65 vs 7.60 ± 1.37), as was the progression index (mean ± SD: 0.15 ± 0.12 vs 0.45 ± 0.28) which is computed as disability grade divided by the duration of the disease.

**Table 1:** Basic characteristics of the group of 270 MS patients, stratified into 189 patients with RRMS and 81 patients with PPMS.

	189 RRMS patients		81 PPMS patients	
*Gender*	n (%)	95% CI	n (%)	95% CI
**Male**	50 (26.5)	20.1–32.8	44 (54.3)	43.3–65.3
**Female**	139 (73.5)	67.2–79.9	37 (45.7)	34.7–56.7
***Basic parameters***	**mean ± SD**	**95% CI**	**mean ± SD**	**95% CI**
**Age (yrs)**	46.4 ± 10.3	44.9–47.9	58.1 ± 10.9	55.7–60.5
**Age at diagnosis (yrs)**	28.8 ± 8.7	27.5–30.0	37.6 ± 10.4	35.3–39.9
**Last EDSS**	2.21 ± 1.65	1.97–2.45	7.60 ± 1.37	7.30–7.90
**Progression index**	0.15 ± 0.12	0.13–0.16	0.45 ± 0.28	0.39–0.51

SD = standard deviation; CI = confidence interval

### HLA entropy

**Fig 1:**
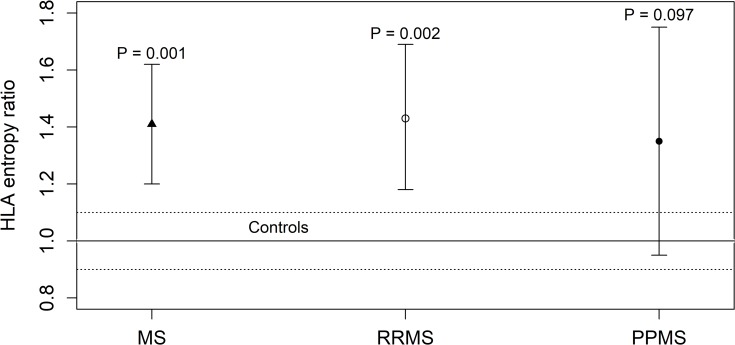
Ratio *R*_*HLA*_ between the HLA entropy of 270 MS patients and the mean HLA entropy of 619 healthy controls. The 270 MS patients were stratified into 81 patients with PPMS and 189 patients with RRMS. The error bars represent the 95% confidence intervals of the HLA entropy ratio of each group of patients. The solid line represents the HLA entropy ratio of the control group (*R*_*HLA*_ = 1) whereas the dotted lines delimit the corresponding 95% confidence interval.

We compared the HLA entropy *S*_*HLA*_ of a cohort of 619 Sardinian healthy controls to the HLA entropy of the total number of MS patients and the subgroups of 189 patients with RRMS and 81 patients with PPMS. The results of comparisons between patients and controls are shown in **Table 2** and **Fig 1**. We also computed the HLA entropy ratio *R*_*HLA*_ between the HLA entropy of each group of patients and the mean HLA entropy of the healthy controls.

**Table 2:** The HLA entropy *S*_*HLA*_ of healthy controls compared to the HLA entropy of MS patients with either PPMS or RRMS.

Samples	Size	*S*_*HLA*_ (95% CI)	*R*_*HLA*_ (95% CI)	P value
**Controls**	619	1.11 (1.00–1.22)	1.00 (0.90–1.10)	
**MS**	270	1.56 (1.32–1.79)	1.41 (1.20–1.62)	**0.001**
**- RRMS**	189	1.59 (1.31–1.87)	1.43 (1.18–1.69)	**0.002**
**- PPMS**	81	1.49 (1.05–1.94)	1.35 (0.95–1.75)	0.097

*R*_*HLA*_ is the ratio between the HLA entropy of patients and the mean HLA entropy of controls.

The mean HLA entropy of controls was 1.11. The HLA entropy ratio *R*_*HLA*_ of MS patients was higher than that of the control group (*R*_*HLA*_ = 1.41, 95% CI = 1.20–1.62, P = 0.001). Also the *R*_*HLA*_ of RRMS patients was significantly higher (*R*_*HLA*_ = 1.43, 95% CI = 1.18–1.69, P = 0.002) in comparison to the controls, as shown by the corresponding error bars in Fig 1 which lie above the dotted range (95% CI = 0.90–1.10). Interestingly, no significant differences were found for PPMS patients (P = 0.097). In fact, the corresponding error bar (95% CI = 0.95–1.75) intersects the dotted range.

### KIR entropy

**Fig 2:**
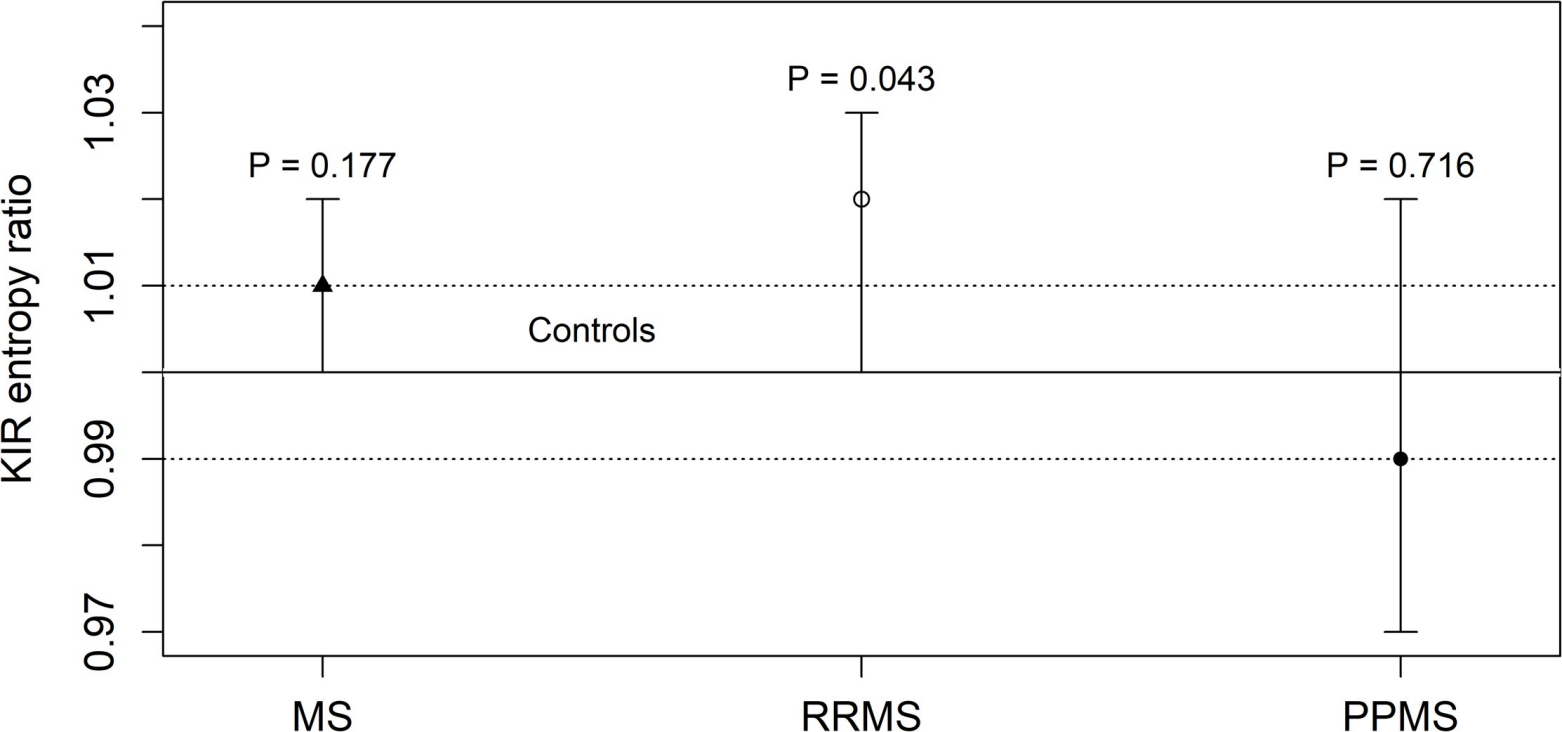
Ratio *R*_*KIR*_ between the KIR entropy of 270 patients affected by MS and the mean KIR entropy of 619 healthy controls. The 270 MS patients were stratified into 81 patients with PPMS and 189 patients with RRMS. The error bars represent the 95% confidence intervals of the KIR entropy ratio of each group of patients. The solid line represents the KIR entropy ratio of the control group (*R*_*KIR*_ = 1) whereas the dotted lines delimit the corresponding 95% confidence interval.

Class I molecules of the HLA system are specific ligands for KIRs. This prompted us to include the KIR gene system in our investigation of entropy. **Table 3** and **Fig 2** show the results of the comparison between the KIR entropy *S*_*KIR*_ of a cohort of 619 healthy controls and a group of 270 MS patients, stratified into two groups of patients affected by RRMS or PPMS. We also computed the KIR entropy ratio *R*_*KIR*_ between the KIR entropy of each group of patients and the mean of the KIR entropy of the control cohort.

**Table 3:** The KIR entropy *S*_*KIR*_ of 619 healthy controls compared to the KIR entropy of MS patients, stratified according to the diagnosis of PPMS or RRMS.

Samples	Size	*S*_*KIR*_ (95% CI)	*R*_*KIR*_ (95% CI)	P value
**Controls**	619	60.15 (59.60–60.71)	1.00 (0.99–1.01)	
**MS**	270	60.81 (60.04–61.57)	1.01 (1.00–1.02)	0.177
**- RRMS**	189	61.22 (60.35–62.08)	1.02 (1.00–1.03)	**0.043**
**- PPMS**	81	59.84 (58.25–61.43)	0.99 (0.97–1.02)	0.716

*R*_*KIR*_ is the ratio between the KIR entropy of patients and the mean KIR entropy of controls.

The KIR entropy ratio *R*_*KIR*_ of patients affected by RRMS was significantly higher than that of the controls (*R*_*KIR*_ = 1.02, 95% CI = 1.00–1.03, P = 0.043). No significant differences were found for the total group of MS patients or the subgroup of patients with PPMS.

The statistical significance obtained for the difference between the KIR entropy of RRMS patients and controls was lower than that found for the HLA entropy. A plausible explanation is that the number of different HLA four-loci haplotypes in both groups of RRMS patients and controls were almost eight hundred (**S1 Table**), while the number of combinations of the fourteen KIR genes in either group of controls or RRMS patients were less than forty (**S2 Table**). Consequently, the degree of disorder–measured by entropy–in the KIR system could not be as high as that observed in the HLA system.

In the supplementary **S1 Table**, we listed the frequencies of the 30 distinct HLA haplotypes satisfying Cochran’s rule [24] (i.e. with expected frequencies greater than 5), observed in both groups of RRMS patients and controls. Analogously, in the **S2 Table** we listed the 9 distinct KIR gene haplotypes observed in either group of controls or RRMS patients, which satisfy Cochran’s rule.

### Total entropy

As discussed at the end of the subsection “HLA and KIR entropy” in the Materials and Methods section, if both the HLA and KIR profiles of the patients are known, it is possible to consider the total entropy ratio *R*_*tot*_, which is equal to the mean of the corresponding HLA and KIR entropy ratios:

$$R_{tot}=\frac{1}{2}(R_{HLA}+R_{KIR}).$$

**Fig 3:**
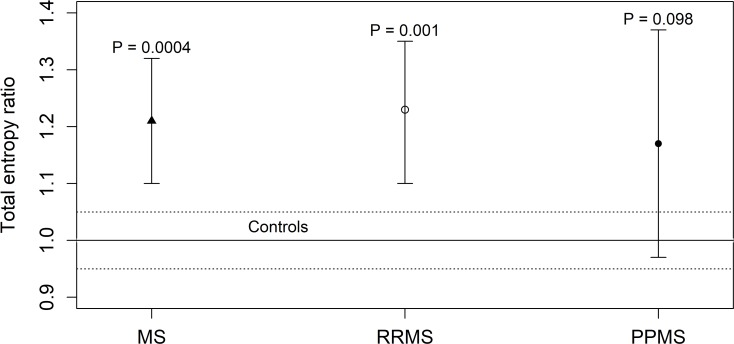
Total entropy ratio *R*_*tot*_ of 270 patients affected by MS in comparison to a cohort of 619 healthy controls. The total entropy ratio *R*_*tot*_ *is* given by the mean of the HLA and KIR entropy ratios *R*_*HLA*_ and *R*_*KIR*_. The 270 patients affected by MS were stratified into 81 patients with PPMS and 189 patients with RRMS The error bars represent the 95% confidence intervals of the total entropy ratio *R*_*tot*_ in each group of patients. The total entropy ratio of the control cohort (*R*_*tot*_ = 1) is represented by the solid line whereas the dotted lines delimit the corresponding 95% confidence interval.

The comparisons between patients and controls are shown in **Table 4** and **Fig 3**.

**Table 4:** Total entropy ratio *R*_*tot*_, given by the mean of the HLA and KIR entropy ratios *R*_*HLA*_ and *R*_*KIR*_, in a group of 270 patients affected by MS (stratified into patients with PPMS and RRMS) in comparison to a cohort of 619 healthy controls.

Samples	Size	*R*_*tot*_ (95% CI)	P value
**Controls**	619	1.00 (0.95–1.05)	
**MS**	270	1.01 (1.00–1.02)	**4.4∙10**^**−4**^
**- RRMS**	189	1.23 (1.10–1.35)	**0.001**
**- PPMS**	81	1.17 (0.97–1.37)	0.098

The total entropy of the RRMS patients was significantly higher than that of the controls (P = 0.001). In fact, the corresponding error bar (95% CI = 1.10–1.35) in **Fig 3** lies above the dotted range (95% CI = 0.95–1.05). Conversely, the total entropy of the PPMS patients was not significantly different from the total entropy of the controls (P = 0.098). In fact the corresponding error bar (95% CI = 0.97–1.37) intersects the dotted range.

**S1 Fig** illustrates the boxplots for HLA, KIR and total entropy in the group of 619 controls and the three groups of patients (270 patients affected by MS, 189 by RRMS and 81 by PPMS), showing that the HLA and total entropy (but not KIR entropy) was higher in patients affected by RRMS in comparison to healthy controls and patients affected by PPMS.

### Application of the entropy-based risk test in MS patients and controls

As discussed in the subsection “Construction of a risk test based on HLA and KIR entropy” of the Materials and Methods section, we established three degrees of risk for developing RRMS based on three different intervals of the total entropy ratio.

The results are shown below in **Table 5**.

**Table 5:** Risk of RRMS and intervals of total entropy ratios.

Risk degrees	Total entropy ratio intervals	Healthy controls n (%)	Patients with RRMS n (%)	Risk of RRMS	P value*	OR (95% CI)*
Low	*R*_*tot*_<1.05	435 (70.3)	101 (53.4)	-16.9%	**3.2∙10**^**−5**^	0.49 (0.34–0.69)
Medium	1.05≤*R*_*tot*_≤1.10	20 (3.2)	7 (3.7)	+0.5%	0.817	0.81 (0.40–2.89)
High	*R*_*tot*_>1.10	164 (26.5)	81 (42.9)	+16.4%	**3.0∙10**^**−5**^	2.08 (1.46–2.96)

*P values and 95% CIs were computed using the two-tailed Fisher’s exact test.

Subjects with a total entropy ratio in the interval *R*_*tot*_<1.05 had a lower risk of contracting RRMS (OR = 0.49); subjects with a total entropy ratio in the interval *R*_*tot*_>1.10 had a risk twice as high (OR = 2.08); subjects with a total entropy ratio in the interval 1.05≤*R*_*tot*_≤1.10 had an intermediate risk. The risk of RRMS is expressed by the difference between the percentage of RRMS patients and the percentage of healthy controls in the same range of total entropy.

Overall, the intervals of total entropy ratio in MS patients were the same as in RRMS patients. The risk of MS observed in our cohorts of MS patients and controls is shown in **Table 6**.

**Table 6:** Risk of MS and total entropy ratio intervals.

Risk degrees	Total entropy ratio intervals	Healthy controls n (%)	Patients with MS n (%)	Risk of MS	P value*	OR (95% CI)*
Low	*R*_*tot*_<1.05	435 (70.3)	154 (57.0)	-13.3%	**1.5∙10**^**−4**^	0.56 (0.41–0.76)
Medium	1.05≤*R*_*tot*_≤1.10	20 (3.2)	11 (4.1)	+0.9%	0.553	1.27 (0.54–2.83)
High	*R*_*tot*_>1.10	164 (26.5)	105 (38.9)	+12.4%	**2.6∙10**^**−4**^	1.76 (1.29–2.42)

*P values and 95% CIs were computed using the two-tailed Fisher’s exact test.

Subjects with a total entropy ratio in the interval *R*_*low*_<1.05 had a lower risk of contracting MS (OR = 0.56); subjects with a total entropy ratio in the interval *R*_*tot*_>1.10 had a risk nearly twice as high (OR = 1.76); subjects with a total entropy ratio in the interval 1.05≤*R*_*tot*_≤1.10 had an intermediate risk. The risk of MS was expressed by the difference between the percentage of MS patients and the percentage of healthy controls in the same range of total entropy.

Obviously, the risk test for PPMS was not sufficiently sensitive because of the lack of significant differences in total entropy between PPMS patients and controls (**Table 4**).

The risk test for RRMS was also performed considering the HLA and KIR systems separately. The HLA entropy ratio intervals [*R*_*HLA*_<1.10 (low risk), 1.10≤*R*_*HLA*_≤1.18 (medium risk) and *R*_*HLA*_>1.18 (high risk)] and the KIR entropy ratio intervals [*R*_*KIR*_<0.99 (low risk), 0.99≤*R*_*KIR*_≤1.00 (medium risk) and *R*_*KIR*_>1.00 (high risk)] were obtained analogously to the procedure described above for the total entropy (but considering the *lower* endpoint instead of the *upper* endpoint of the 95% CI of the control KIR entropy ratio). The results obtained for the HLA entropy ratio are shown in **Table 7**, whereas the results for the KIR entropy ratio are shown in **Table 8**. The risk test was not sufficiently sensitive when based exclusively on KIR entropy.

**Table 7:** Risk of RRMS and HLA entropy ratio intervals.

Risk degrees	HLA entropy ratio intervals	Healthy controls n (%)	Patients with RRMS n (%)	Risk of RRMS	P value*	OR (95% CI*)
Low	*R*_*HLA*_<1.10	432 (69.8)	103 (54.5)	-15.3%	**1.5∙10**^**−4**^	0.52 (0.37–0.74)
Medium	1.10≤*R*_*HLA*_≤1.18	19 (3.1)	7 (3.7)	+0.6%	0.641	1.21 (0.42–3.08)
High	*R*_*HLA*_>1.18	168 (27.1)	79 (41.8)	+14.7%	**2.0∙10**^**−4**^	1.93 (1.35–2.74)

*P values and 95% CIs were computed using the two-tailed Fisher’s exact test.

**Table 8:** Risk of RRMS and KIR entropy ratio intervals.

Risk degrees	KIR entropy ratio intervals	Healthy controls n (%)	Patients with RRMS n (%)	Risk of RRMS	P value*	OR (95% CI*)
Low	*R*_*KIR*_<0.99	108 (17.4)	22 (11.6)	-5.8%	0.070	0.62 (0.36–1.03)
Medium	0.99≤*R*_*KIR*_≤1.00	71 (11.5)	24 (12.7)	+1.2%	0.699	1.12 (0.65–1.87)
High	*R*_*KIR*_>1.00	440 (71.1)	143 (75.7)	+4.6%	0.230	1.26 (0.86–1.88)

*P values and 95% CIs were computed using the two-tailed Fisher’s exact test.

The method based on total, HLA or KIR entropy ratios made it possible to calculate the individual risk of a subject to develop MS, particularly RRMS.

### Practical example of entropy-based risk assessment in family members of MS patients

This is an example of how entropy can be used to individuate susceptibility to MS in family members of patients with different forms of MS.

**Fig 4:**
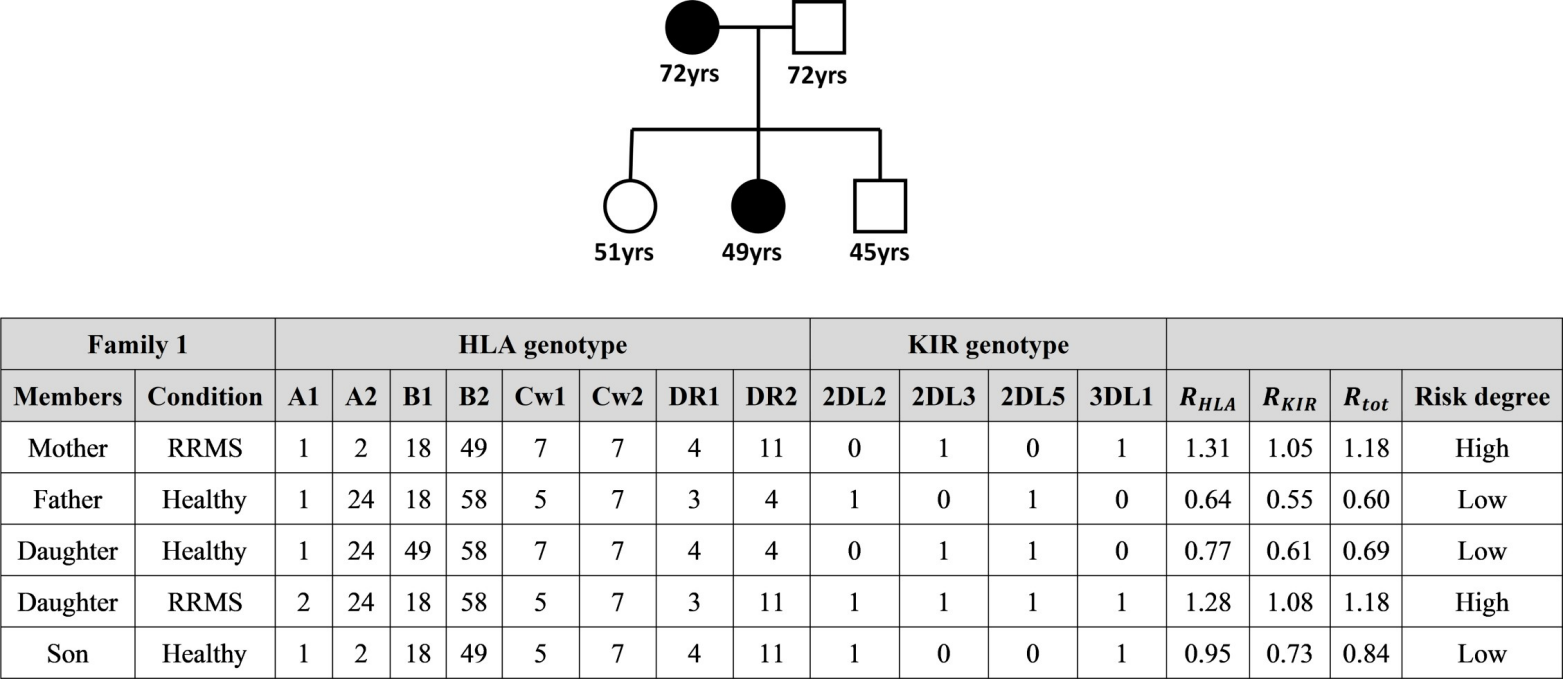
Entropy-based risk assessment in family members of a patient affect by RRMS. Total entropy ratio *R*_*tot*_ is given by the mean of the HLA and KIR entropy ratios *R*_*HLA*_ and *R*_*KIR*_. High risk: *R*_*tot*_ > 1.10; Low risk: *R*_*tot*_ < 1.05.

Family 1 is composed of 5 members and is illustrated in **Fig 4**.

The entropy-based test revealed a high risk of MS in the mother and one of the daughters (*R*_*tot*_ = 1.18), both affected by RRMS, and a low risk for the healthy father (*R*_*tot*_ = 0.60), son (*R*_*tot*_ = 0.84) and daughter (*R*_*tot*_ = 0.69).

**Fig 5:**
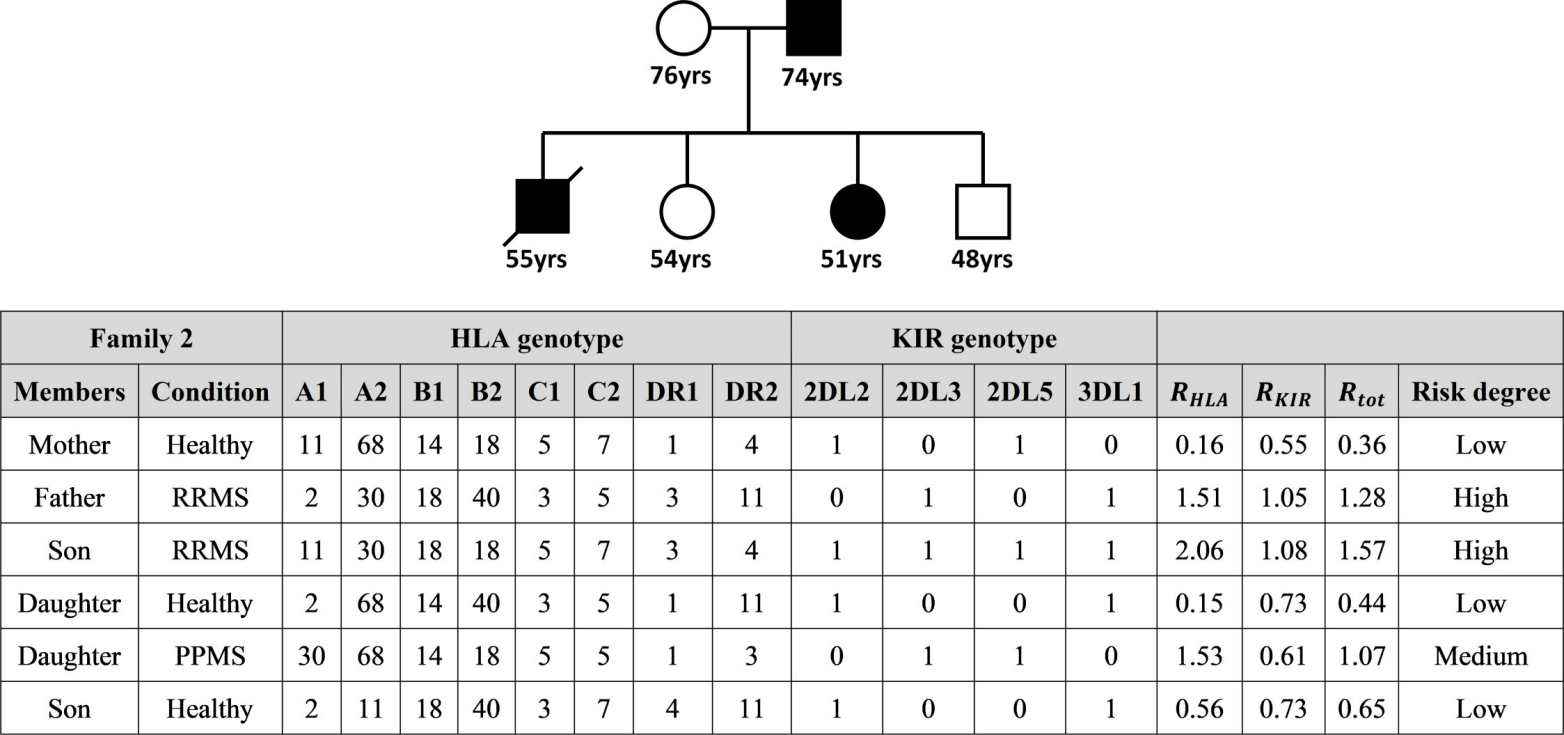
Entropy-based risk assessment in family members of a patient affected by RRMS. Total entropy ratio *R*_*tot*_ is given by the mean of the HLA and KIR entropy ratios *R*_*HLA*_ and *R*_*KIR*_. High risk: *R*_*tot*_ > 1.10; Low risk: *R*_*tot*_ < 1.05; Medium risk: 1.05 ≤ *R*_*tot*_ ≤ 1.10.

Family 2 is composed of 6 members and is shown in **Fig 5**.

Also in this retrospective analysis, the application of entropy was able to assign a high risk for MS to the father and deceased son (*R*_*tot*_ = 1.28 and *R*_*tot*_ = 1.57, respectively), both affected by RRMS, a medium risk to one of the daughters (*R*_*tot*_ = 1.07), affected by PPMS, and a low risk to the healthy mother, son and daughter (*R*_*tot*_ = 0.36, *R*_*tot*_ = 0.65, *R*_*tot*_ = 0.44, respectively).

## Discussion

Entropy might be the key to explaining what life is, as suggested by Erwin Schrödinger in his renowned book published in 1944 “What is life?” [1], which also inspired the work of Watson, Crick and Wilkins [25, 26] on deoxyribonucleic acid (DNA).

In 1948, Claude Shannon introduced a definition of entropy which could be adapted to a variety of disciplines [2], including the study of complex genetic mechanisms underlying the development of disease. Unfortunately, the understanding of genetics in those years was not sufficient to exploit entropy as a measure of the disorder associated to genetic systems, as probably Shannon himself hoped [27].

In the literature, the HLA system has been unambiguously associated with the development of MS, although associations vary among different populations. In European populations, MS is associated to the HLA-DR*15, DQ*06 haplotype [28, 29]. In the Sardinian population, MS has been found associated with the HLA-A*30, -B*18, -C*05, -DR*03 extended haplotype and the HLA-DR*03 and HLA-DQ*02 alleles [30, 31]. While many authors link MS to the HLA system, only a minority of studies have reported association between MS and KIR genes and the results are either contradictory or remain unconfirmed [32–34]. All these studies applied standard statistical tests which are useful in detecting single alleles and/or haplotypes that either confer protection or susceptibility to disease.

In our cohorts of patients and controls, association of HLA with MS was also evaluated by comparing the frequencies of HLA haplotypes and alleles in patients and controls (**S1 Table**). The results confirmed the association of MS with the HLA-A*30, -B*18, -C*05, -DR*03 extended haplotype (19.1% vs 12.4%; Pc = 0.023) and the well-known HLA susceptibility allele HLA-DR*03 (30.9% vs 22.0%; Pc = 9.2 x 10^−4^). However, these standard methods only show small differences in frequencies between patients and controls. Overall, they provide information that is not sufficient to reliably predict the risk of developing MS, especially in individuals who do not possess the aforementioned genetic characteristics.

The aim of the innovative entropy-based approach described here was to evaluate the genetic disorder related to the HLA or KIR system as a whole. The sensitivity of the entropy-based test was also confirmed by the analysis of the KIR gene cluster since standard approaches alone did not detect significant differences between the KIR gene profiles of patients and controls.

The HLA entropy of patients affected by RRMS was significantly higher than the HLA entropy of the controls (P = 0.002). A similar finding was obtained for KIR entropy, but with a lower level of significance (P = 0.043). No differences were observed for PPMS patients.

When analyzing the HLA and KIR systems combined, the total entropy was significantly higher in patients affected by RRMS (P = 0.001) compared to controls. On the other hand, no significant differences were observed when comparing the total entropy of healthy controls with that of patients affected by PPMS. Therefore, it is likely that the HLA and KIR systems do not have a relevant role in the pathogenesis of PPMS. This statement is furthermore supported by the absence of susceptibility HLA haplotypes and/or alleles in PPMS patients in this (**S1 Table**) and other studies [35]. The scarce influence of HLA and KIR systems in this form of MS may at least partially explain why PPMS patients respond poorly to immunomodulatory therapy with corticosteroids and drugs such as glatiramer acetate or interferon-beta [36].

In our study, the entropy of the complex KIR gene system was calculated by considering combinations of two KIR genes chosen from a subset of four inhibitory KIR genes selected by a logistic regression model. However, other approaches might be capable of providing a more effective evaluation of KIR entropy in immune-mediated disorders.

To the best of our knowledge, there are no studies in the literature describing association of Shannon’s entropy with immune-mediated disorders, apart from general discussions on the role of entropy in the fields of genetics and biology [37, 38]. The entropy-based risk test developed in this study was capable of detecting susceptibility to RRMS in 42.9% of patients (**Table 5**). The test was less sensitive for PPMS, possibly because the immunogenetic parameters chosen for this study have a minor role in this form of MS. The sensitivity of the entropy-based risk test would certainly benefit from the inclusion of other susceptibility genes such as those recently emerging from genome-wide association studies on MS and other immunogenetic disorders [39, 40].

The results of our pilot study suggest that the total entropy of the HLA and KIR systems may also be capable of associating the HLA and KIR profile of a subject with the risk of developing other diseases with an immune-mediated pathogenesis such as type 1 diabetes, Hashimoto’s thyroiditis, celiac disease, psoriasis, rheumatoid arthritis and systemic lupus erythematosus.

Overall, entropy can be adapted to the study of complex genetic systems and multifactorial diseases but requires standard methods of analysis to identify the genetic parameters necessary for the construction of the entropy-based algorithm. The introduction of entropy into clinical practice could provide precious support to the currently available methods for risk assessment of immune-mediated diseases. The specificity of entropy is likely to increase in proportion to the amount of immunogenetic data analyzed. It can be postulated that the more complex and data-rich systems are, the more the entropic pattern of a disease will be clear and specific. Ideally, the combined efforts of researchers could contribute to the construction of refined entropic patterns for many immune-mediated disorders. Our investigation of only two important immunogenetic systems cannot completely exclude a certain degree of overlap between the entropic patterns of MS and other immune-mediated diseases. Refined patterns of entropy could possibly improve discrimination between immune-mediated disorders that share etiological similarities and genetic susceptibility factors. Therefore, we strongly encourage researchers to verify the effectiveness of this approach in MS and other pathologies with a strong immune component.

## Supporting information

S1 File Shannon’s entropy: How it works in detail. (PDF)

S2 File Entropy ratio cutoffs. (PDF)

S3 File Sample size evaluation. (PDF)

S4 File Normality and skewness of entropy data. (PDF)

S5 File Cochran’s rule. (PDF)

S1 Table HLA alleles and haplotypes in patient and control cohorts. (PDF)

S2 Table KIR genes and haplotypes in patient and control cohorts. (PDF)

S3 Table Frequencies and entropies of KIR gene couples and HLA haplotypes. (PDF)

S4 Table Linkage disequilibrium. (PDF)

S1 Fig Boxplots. The following boxplots represent Shannon’s entropy associated to HLA-A, -B, -C, -DR haplotypes and couples of inhibitory KIR genes in a cohort of 270 patients affected by multiple sclerosis (MS), stratified into a group of 81 patients with primary progressive multiple sclerosis (PPMS) and a group of 189 patients with relapsing remitting multiple sclerosis (RRMS). The HLA, KIR and total entropies for each group of patients were compared to the respective entropies of a group of 619 healthy controls. In the box and whisker plots, the whiskers represent the extremities of the interquartile ranges (IQR), i.e. the lower and upper quartiles, while the median of each sample is marked as a bold line. The three boxplots clearly show that the median of the HLA and total entropy is higher in patients affected by RRMS in comparison to healthy controls and patients affected by PPMS.
(TIFF)
