# Supplementary material for: Entropy of human leukocyte antigen and killer-cell immunoglobulin-like receptor systems in immune-mediated disorders: A pilot study on multiple sclerosis
Source: PLoS One. 2019 Dec 17;14(12):e0226615. doi: 10.1371/journal.pone.0226615 (PMC6917289; doi:10.1371/journal.pone.0226615)
Supplement: S1 File — (PDF) [file pone.0226615.s001.pdf]

S1 File. Shannon's entropy: how it works in detail

Theoretical framework

If $f_1, f_2, \dots, f_n$ are the probabilities of a set of n events, Shannon's entropy is defined by

$$S = -k \sum_{i=1}^{n} f_i \log f_i$$

where k is a positive constant depending on the choice of the units of measurement. Shannon's entropy is the only function of $f_1, f_2, \dots, f_n$ satisfying the following properties:

- S is continuous in the f_i;
- if all the f_i are equal ($f_i = 1/n$), S is a monotonic increasing function of n;
- if an event can occur in two successive steps, the total Shannon's entropy S is the weighted sum of the values corresponding to each step.

If there are only two possible events, with probabilities f and $q = 1 - f$, Shannon's entropy becomes

$$S = -k\,[f \log f + (1 - f) \log(1 - f)]\,.$$

This form of S is plotted in the Figure below as a function of f (with $k = 100$).

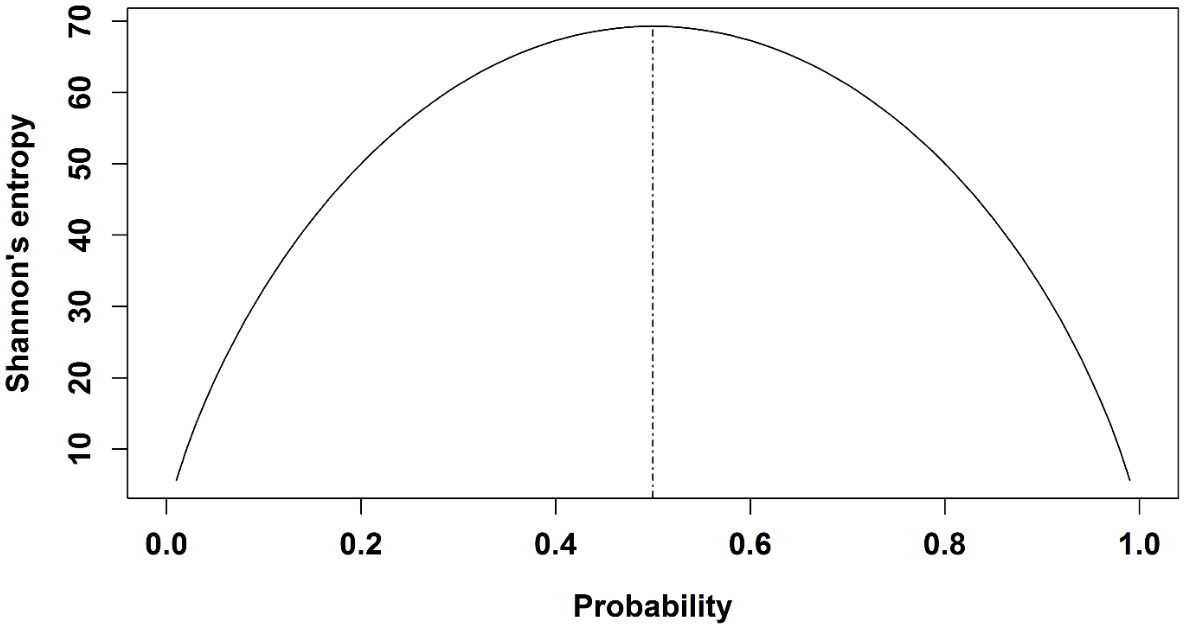

The maximum of Shannon's entropy, obtained for $f = \frac{1}{2}$, is $S_{max} = 69.3$ if $k = 100$.

The scheme below represents the HLA genotype of a subject:

HLA-A		HLA-B		HLA-C		HLA-DR	
a_1	a_2	b_1	b_2	c_1	c_2	d_1	d_2

More specifically, the parameters a_h , b_j , c_l , d_m (with $h, j, l, m = 1$ or 2) are defined by:

$a_h =$ HLA-A*x_h, $b_j =$ HLA-B*x_j, $c_l =$ HLA-Cw*x_l, $d_m =$ HLA-DR*x_m, where the x's are integers indicating specific HLA alleles (for instance, A*30, B*18, C*05, DR*03).

The sixteen possible HLA haplotypes $a_h b_j c_l d_m$ for each subject are:

$a_1b_1c_1d_1$, $a_1b_1c_1d_2$, $a_1b_1c_2d_1$, $a_1b_1c_2d_2$, $a_1b_2c_1d_1$, $a_1b_2c_1d_2$, $a_1b_2c_2d_1$, $a_1b_2c_2d_2$,

$a_2b_1c_1d_1$, $a_2b_1c_1d_2$, $a_2b_1c_2d_1$, $a_2b_1c_2d_2$, $a_2b_2c_1d_1$, $a_2b_2c_1d_2$, $a_2b_2c_2d_1$, $a_2b_2c_2d_2$.

For each subject, the Shannon's entropy associated to the ith HLA haplotype with probability f_i (where $i = 1, 2, \ldots, 16$) is:

$$S_i = -k[f_i \log f_i + (1 - f_i) \log(1 - f_i)].$$

The HLA entropy of each subject is the mean of the entropies associated to all the sixteen possible HLA haplotypes:

$$S_{HLA} = -\frac{k}{N}\sum_{i=1}^{N}[f_i \log f_i + (1 - f_i) \log(1 - f_i)] \text{, with } N = 16.$$

No theoretical model exists to derive the values of the HLA haplotype probabilities; for this reason, the parameters f_i appearing in Shannon's formula for HLA entropy were obtained experimentally from the control group.

The scheme below represents the fourteen KIR genes:

2DL1	***2DL2***	***2DL3***	***2DL4***	***2DL5***	***3DL1***	***3DL2***	***3DL3***	***2DS1***	***2DS2***	***2DS3***	***2DS4***	***2DS5***	***3DS1***
k_1	k_2	k_3	k_4	k_5	k_6	k_7	k_8	k_9	k_{10}	k_{11}	k_{12}	k_{13}	k_{14}

where the presence or absence of the ith KIR gene (with $i = 1, 2, \ldots, 14$) is indicated by the values of the parameter k_i ($k_i = 0$ indicates that the ith KIR gene is absent, while $k_i = 1$ indicates that it

is present). We only considered couples of inhibitory KIRgenes: k_h , $k_j$with $h, j \in \{2, 3, 5, 6\}$, i.e. k_h , $k_j \in \{2DL2, 2DL3, 2DL5, 3DL1\}$.

The number of possible KIR gene couples from a set of four inhibitory KIR genes is $\binom{4}{2} = 6$. For each subject the KIR entropy is the mean of the entropies associated to these six possible KIR gene couples:

$$S_{KIR} = -\frac{k}{N}\sum_{i=1}^{N}[f_i \log f_i + (1 - f_i)\log(1 - f_i)]\text{ , with } N = 6.$$

The probabilities f_i (with $i = 1, 2, \cdots, 6$) of each KIR gene couple is obtained experimentally from the control cohort.

The choice of considering KIR gene couples from a specific set of four inhibitory KIR genes was based on the response of a logistic regression model exploited to select the KIR genes which were more influent in determining significant differences between the KIR entropy of RRMS patients and controls.

From HLA and KIR genotype to entropy: a detailed example

In this Section, we provide a detailed example of the method used to evaluate entropy starting from the HLA and KIR genotype of a patient. The value obtained with this risk test for patient entropy indicates the probability of the patient to contract RRMS.

**HLA-A**		**HLA-B**		**HLA-C**		**HLA-DR**	
2	32	18	58	7	7	11	16

S3 Table lists all the combinations of HLA four-loci haplotypes present in the control group, with the corresponding frequencies and entropies.

The frequenciesf_i and entropies $S_{HLA;\,i}$ of the 16 combinations of HLA four-loci haplotypes present in the patient are given below:

**Index (i)**	**HLA haplotypes**		**Frequency (f_i)**	**Entropy ($S_{HLA;\,i}$)**
1	$a_1b_1c_1d_1$	A*02, B*18, C*07, DR*11	0.00342	2.28
2	$a_1b_1c_1d_2$	A*02, B*18, C*07, DR*16	0.00434	2.79
3	$a_1b_1c_2d_1$	A*02, B*18, C*07, DR*11	0.00342	2.28
4	$a_1b_1c_2d_2$	A*02, B*18, C*07, DR*16	0.00434	2.79
5	$a_1b_2c_1d_1$	A*02, B*58, C*07, DR*11	0.00223	1.58
6	$a_1b_2c_1d_2$	A*02, B*58, C*07, DR*16	0.01077	5.95
7	$a_1b_2c_2d_1$	A*02, B*58, C*07, DR*11	0.00223	1.58
8	$a_1b_2c_2d_2$	A*02, B*58, C*07, DR*16	0.01077	5.95
9	$a_2b_1c_1d_1$	A*32, B*18, C*07, DR*11	0.00066	0.55
10	$a_2b_1c_1d_2$	A*32, B*18, C*07, DR*16	0.00289	1.98
11	$a_2b_1c_2d_1$	A*32, B*18, C*07, DR*11	0.00066	0.55
12	$a_2b_1c_2d_2$	A*32, B*18, C*07, DR*16	0.00289	1.98
13	$a_2b_2c_1d_1$	A*32, B*58, C*07, DR*11	0.00013	0.13
14	$a_2b_2c_1d_2$	A*32, B*58, C*07, DR*16	0.00184	1.34
15	$a_2b_2c_2d_1$,	A*32, B*58, C*07, DR*11	0.00013	0.13
16	$a_2b_2c_2d_2$	A*32, B*58, C*07, DR*16	0.00184	1.34

The mean of the entropies of the previous 16 combinations of the patient's HLA haplotypes is:

$$S_{HLA} = -\frac{k}{N}\sum_{i=1}^{N}[f_i \log f_i + (1 - f_i) \log(1 - f_i)] = \frac{1}{16}\sum_{i=1}^{16} S_{HLA;\,i} = 2.08,$$

with $N = 16$ and $k = 100$.

The mean HLA entropy of the control group is $S_{HLA;\,ctr} = 1.106$, hence the patient HLA entropy ratio is

$$R_{HLA} = \frac{S_{HLA}}{S_{HLA;\,ctr}} = 1.88\,.$$

Let the KIR genotype of the patient be

2DL1	***2DL2***	***2DL3***	***2DL4***	***2DL5***	***3DL1***	***3DL2***	***3DL3***	***2DS1***	***2DS2***	***2DS3***	***2DS4***	***2DS5***	***3DS1***
1	0	1	1	0	1	1	1	0	0	0	1	0	0

The inhibitory KIR genes (excluding those with percentages higher than 90%) are

(*2DL2*, *2DL3*, *2DL5*, *3DL1*) = (0, 1, 0, 1).

S3 Table lists the frequencies of the 24 couples of inhibitory KIR genes observed in the group of controls and the corresponding KIR entropies.

The frequencies f_i and entropies $S_{KIR;\,i}$ of the 6 couples of inhibitory KIR genes present in the patient are given below:

Index (i)	KIR gene couples		Frequency (f_i)	Entropy ($S_{KIR;\,i}$)
1	(*2DL2*, *2DL3*)	(0, 1)	0.42165	68.08
2	(*2DL2*, *2DL5*)	(0, 0)	0.29402	60.57
3	(*2DL2*, *3DL1*)	(0, 1)	0.40872	67.64
4	(*2DL3*, *2DL5*)	(1, 0)	0.42649	68.23
5	(*2DL3*, *3DL1*)	(1, 1)	0.82553	46.29
6	(*2DL5*, *3DL1*)	(0, 1)	0.44426	68.69

The mean of the entropies of the previous 6 KIR gene couples is:

$$S_{KIR} = -\frac{k}{N}\sum_{i=1}^{N}[f_i \log f_i + (1 - f_i)\log(1 - f_i)] = \frac{1}{6}\sum_{i=1}^{6} S_{KIR;\,i} = 63.25,$$

with $N = 6$ and $k = 100$.

The mean KIR entropy of the control group is $S_{KIR;\,ctr} = 60.15$, hence the patient KIR entropy ratio is

$$R_{KIR} = \frac{S_{KIR}}{S_{KIR;\,ctr}} = 1.05.$$

The total entropy ratio is

$$R_{tot} = \frac{1}{2}(R_{HLA} + R_{KIR}) = 1.47.$$

Being $R_{tot} = 1.47$ greater than the cutoff $R_{tot} > 1.10$ of the high entropy ratio interval, the patient has a high risk of contracting RRMS.

The HLA and KIR entropy ratios $R_{HLA} = 1.88$ and $R_{KIR} = 1.05$ are also greater than the cutoffs $R_{HLA} > 1.18$ and $R_{KIR} > 1.00$ of the corresponding high entropy ratio intervals, confirming that the patient is exposed to a high risk of contracting RRMS.
