# Supplementary material for: Entropy of human leukocyte antigen and killer-cell immunoglobulin-like receptor systems in immune-mediated disorders: A pilot study on multiple sclerosis
Source: PLoS One. 2019 Dec 17;14(12):e0226615. doi: 10.1371/journal.pone.0226615 (PMC6917289; doi:10.1371/journal.pone.0226615)
Supplement: S2 File — (PDF) [file pone.0226615.s002.pdf]

S2 File. Entropy ratio cutoffs

The cutoffs which delimit the entropy ratio intervals corresponding to the three degrees of risk (low, medium and high) were chosen in order to comply with the following criteria:

1. Maximize the absolute value of the differences between the percentages of patients and controls whose entropy ratios were included in the intervals corresponding to the *low* or *high* degrees of risk.
2. minimize the difference between the percentages of patients and controls with entropy ratios included in the interval corresponding to the *medium* degree of risk.
3. guarantee a number of observations in each of the three entropy ratio intervals (corresponding to the *three risk degrees*) sufficiently large to satisfy the Cochran's rule (more than 5 expected frequencies in patient-control comparisons).

The absolute value of the differences between the percentages of RRMS patients and healthy controls with total entropy ratios (R_{tot}) included in the intervals associated to the low and high degrees of risk turned out to be maximum for $R_{tot} < 1.07$ and $R_{tot} > 1.11$, respectively. Although these total entropy ratio cutoffs were those which better satisfied the first two aforementioned criteria, they did not match the third criterium since the observations within the total entropy ratio interval associated to medium risk ($1.07 \leq R_{tot} \leq 1.11$) were not sufficiently numerous.

On the contrary, the cutoffs corresponding to the upper endpoint of the 95% CI of the control total entropy ratio and to the lower endpoint of the 95% CI of the RRMS patient total entropy ratio, i.e. $R_{tot} < 1.05$ and $R_{tot} > 1.10$, respectively, also satisfied the third criterium with observations within the medium interval $1.05 \leq R_{tot} \leq 1.10$ that were sufficiently numerous. These cutoffs determined differences between RRMS patients and controls that were very close to those obtained for the previously considered total entropy ratio cutoffs. For this reason the choice of these 95% CI endpoints as cutoffs of the total entropy ratio intervals for the three risk degrees was considered to be the most appropriate. An analogous conclusion was drawn for the HLA and KIR entropy ratio cutoffs.

The Figure below represents the total entropy ratio intervals exploited in the risk test for RRMS. The upper endpoint of the control 95% CI (0.95 – **1.05**) and the lower endpoint of the RRMS patient 95% CI (**1.10** – 1.35) were chosen as cutoffs for the total entropy ratio intervals corresponding to the three degrees of risk of contracting RRMS.

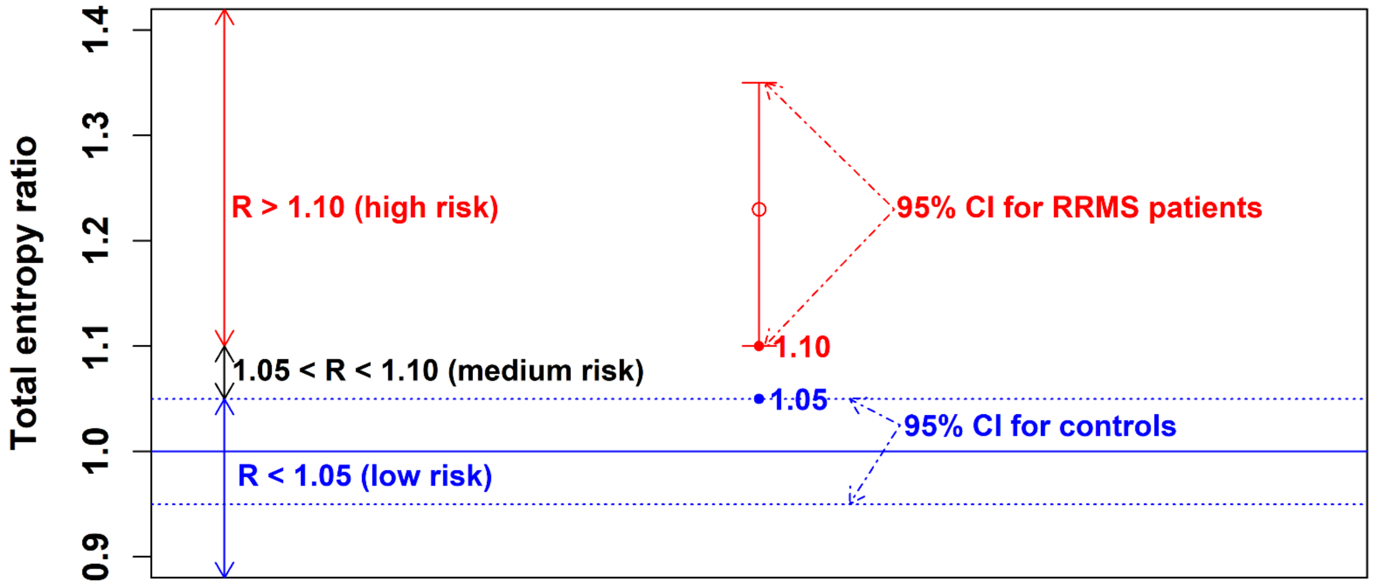

Analogous figures can be pictured for the intervals of HLA and KIR entropy corresponding to the three degrees of risk of developing MS or RRMS.
