# Supplementary material for: Entropy of human leukocyte antigen and killer-cell immunoglobulin-like receptor systems in immune-mediated disorders: A pilot study on multiple sclerosis
Source: PLoS One. 2019 Dec 17;14(12):e0226615. doi: 10.1371/journal.pone.0226615 (PMC6917289; doi:10.1371/journal.pone.0226615)

S3 File. Sample size evaluation

Before performing a pilot study, a power calculation can be carried out to determine the minimum sample size needed to identify statistically significant differences. However, such calculations are based on the results of previous studies in the scientific literature. Our study is the first to investigate HLA and KIR entropy in patients and controls and therefore sample size evaluation only allowed us to establish the minimum difference that our investigation could detect in the case and control groups.

The minimum statistically significant difference detectable between the means μ_1 and μ_2 of a given variable in two groups with sizes n_1 and n_2 is given by the following *sample size formula*[1]:

$$(\mu_1 - \mu_2)^2 = K\left(\frac{{\sigma_1}^2}{n_1} + \frac{{\sigma_2}^2}{n_2}\right)$$

where$\sigma_1$and $\sigma_2$are the standard deviations of that variable in the two groups and K is a constant depending on significance level and power of the investigation, as shown in the Table below:

**Study power**	**Significance level**		
	**5%**	**1%**	**0.01%**
**80%**	7.8	11.7	17.1
**90%**	10.5	14.9	20.9
**95%**	13.0	17.8	24.3
**99%**	18.4	24.1	31.6

The study power is often set at 90% and the significance level at 5%, therefore K = 10.5.

The present study comprises n_1 =189 RRMS patients and n_2 =619 healthy controls. According to the aforesaid sample size formula, the expected minimum statistically significant difference $|R_1 - R_2|$ between the means of the entropy ratios in the two groups is close to the results observed in our investigation for the HLA, KIR and total entropy ratios, as shown in the following Table.

[1] Bland JM. An Introduction to Medical Statistics (4th edition). Oxford University Press (Oxford, 2015).

	Entropy ratio ($R \pm \sigma$)		$\|R_1 - R_2\|$	
	Cases ($n_1 = 189$)	Controls ($n_2 = 619$)	Observed	Expected
**HLA**	1.43 ± 1.75	1.00 ± 1.26	0.43	0.44
**KIR**	1.02 ± 0.10	1.00 ± 0.12	0.02	0.03
**Total**	1.23 ± 0.87	1.00 ± 0.63	0.23	0.22

Power of the investigation

From the sample size formula, we can derive the power of the study by considering the means of the entropy ratios R_1 and R_2 obtained in our investigation and the respective standard deviations $\sigma_1$and σ_2:

$$K = (R_1 - R_2)^2 / \left(\frac{{\sigma_1}^2}{n_1} + \frac{{\sigma_2}^2}{n_2} \right)$$

where n_1 and n_2 are the sizes of the case and control groups.

The constants K's for total, HLA and KIR entropy ratios turned out to be, respectively, $K = 11.4$, $K = 9.9$ and $K = 5.3$. By comparing these results with the values of the constant K in the first Table, one finds that the power of the study is around 90% for the total and HLA entropy ratio (the mean of the corresponding K's is $K = 10.7$), while it is lower for the KIR entropy ratio (less than 80%).

Case:control ratio

Frequently, in case-control studies one has only a limited number of cases available, whereas the controls are readily available. However, as the number of controls per case increases, there is a rapidly diminishing return. Hence, it is rarely worth having more than three controls per case; five or more would seem futile[2].

By increasing the number of controls, the power of the study increases: this can be verified by computing the minimum statistically significant difference between the means of the entropy ratios in the case and control groups and checking that it becomes smaller. The increase in the power of

[2] Taylor JMG. Choosing the number of controls in a matched case-control study, some sample size, power and efficiency considerations. *Statistics in Medicin*e 1986; 5: 29-36.

the study becomes negligible when the size of the control group is three or four times greater than the number of cases.

In our study on HLA and KIR entropy, the size of the control group was three times greater than the size of the RRMS patient cohort (619:189 = 3.3).

If the size of the second sample is h times greater than the size n_1 of the first group, we can substitute $n_2 = h \cdot n_1$ in the previous formula, obtaining: $|R_1 - R_2| = \sqrt{\frac{K}{n_1} \cdot \left({\sigma_1}^2 + \frac{{\sigma_2}^2}{h}\right)}$.

The following plot shows the minimum significant difference detectable through our study against the ratio h between the size of controls and cases (h from 1 to 6). As we can see, the minimum difference does not decrease (and hence the power of the study does not increase significantly) when the control:case ratio becomes greater than 3. As a consequence, it is not worth increasing the number of controls above this threshold.

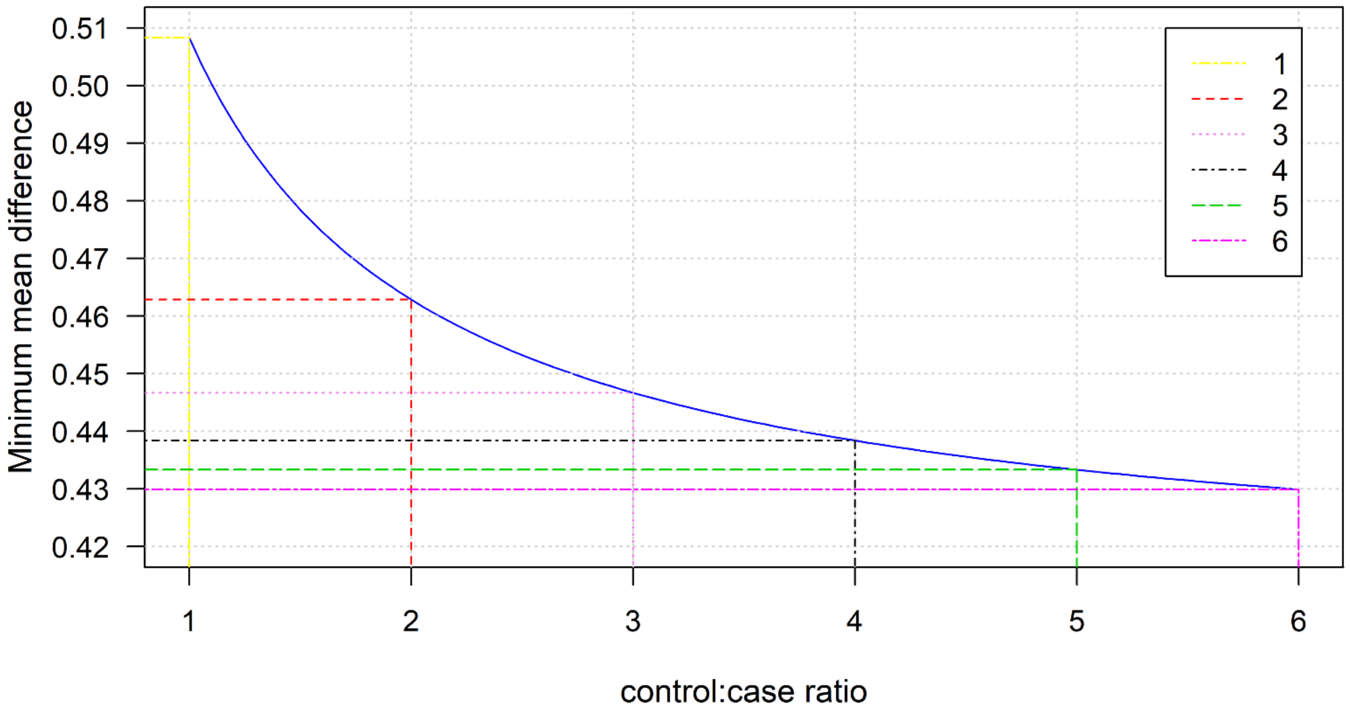

Supplement: S3 File — (PDF) [file pone.0226615.s003.pdf]
