# Supplementary material for: Entropy of human leukocyte antigen and killer-cell immunoglobulin-like receptor systems in immune-mediated disorders: A pilot study on multiple sclerosis
Source: PLoS One. 2019 Dec 17;14(12):e0226615. doi: 10.1371/journal.pone.0226615 (PMC6917289; doi:10.1371/journal.pone.0226615)
Supplement: S4 File — (PDF) [file pone.0226615.s004.pdf]

S4 File. Normality and skewness of entropy data

With large samples, normality and skewness of the data can be checked visually using a histogram. A more accurate method is provided by quantile-quantile or q-q plots, which represent the distribution of the observed frequencies against the corresponding quantiles of the theoretical normal distribution: if the points lie close to the line of equality, then the data can be assumed as normally distributed.

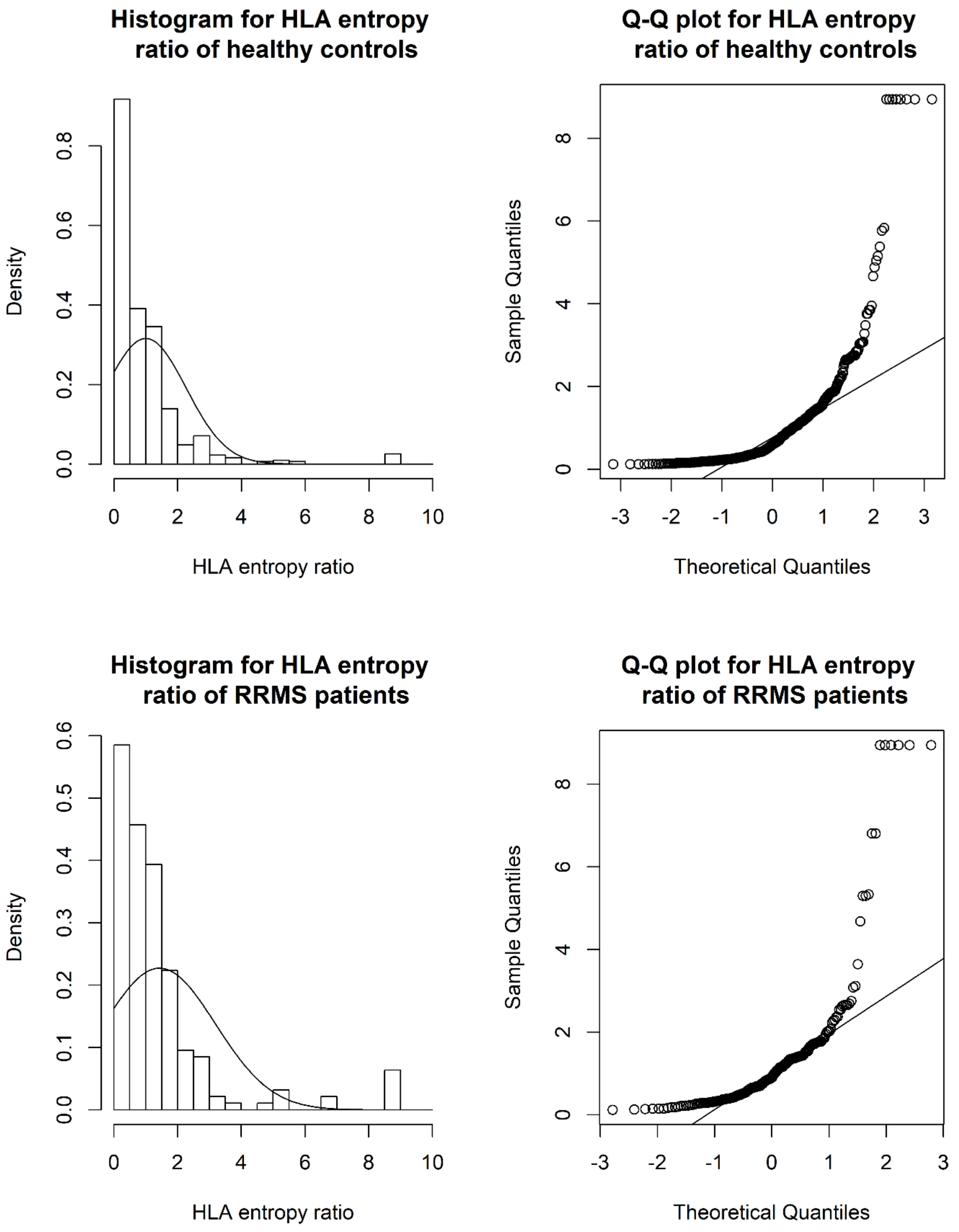

The previous Figures indicate that the distribution of the HLA entropy ratio for controls and RRMS patients is not close to a normal distribution, as confirmed by the Shapiro-Wilk test which provides a P value much less than 5% for both groups of subjects. The histograms also show that the skewness of the HLA entropy ratio distribution is positive, i.e. the data are skewed to the right.

When the data in the cohorts under investigation do not follow a normal distribution, one should use a non-parametric test to compare patients and controls, such as the Mann-Whitney U test (also known as the Wilcoxon rank-sum test), instead of the Student's t test, which is only suitable for normally distributed data in the compared groups. However, the size of our control and case groups was large enough (more than 50 observations in each compared group) to allow for the use of the Student's t test and the Fisher's exact test. The same conclusion can be drawn for the comparison of KIR entropy ratio and total entropy ratio between patients and controls.

The following Table compares the P values obtained with the Student's t test (columns t) to those provided by the non-parametric Mann-Whitney U test (columns U) for the HLA, KIR and total entropy ratios (R_{HLA}, R_{KIR} and R_{tot}).

As shown in the Table, the presence or absence of statistical significance is confirmed by both the parametric Student's t test and the non-parametric Mann-Whitney U test for non-normally distributed data.

**Samples**	**Size**	R_{HLA}	**P value**		R_{KIR}	**P value**		R_{tot}	**P value**	
			**t**	**U**		**t**	**U**		**t**	**U**
**Controls**	619	1.00			1.00			1.00		
**MS**	270	1.41	**0.001**	**$3.7 \cdot 10^{-5}$**	1.01	0.177	0.131	1.21	**$4.4 \cdot 10^{-4}$**	**$2.0 \cdot 10^{-5}$**
**RRMS**	189	1.43	**0.002**	**$6.6 \cdot 10^{-9}$**	1.02	**0.043**	**0.029**	1.23	**0.001**	**$2.3 \cdot 10^{-6}$**
**PPMS**	81	1.35	0.097	0.275	0.99	0.716	0.637	1.17	0.098	0.314

The Table below reports some statistical parameters for the HLA and KIR entropy and for the HLA, KIR and total entropy ratio of healthy controls (619), RRMS patients (189), PPMS patients (81) and MS patients (270). More specifically, the Table lists mean, standard deviation (SD), median, interquartile range (IQR) and variance in each group and for each tested variable.

HLA entropy variance, as well as HLA and total entropy ratio variance, is significantly greater in the patient groups compared to the control group, as confirmed by the F test which provided P values much lower than 5%. On the contrary, KIR entropy variance and KIR entropy ratio variance in the patient groups are similar or slightly lower than the variances in the control group.

	**Healthy controls (619)**			**RRMS patients (189)**			**PPMS patients (81)**			**MS patients (270)**		
	**HLA**	**KIR**	**Total**	**HLA**	**KIR**	**Total**	**HLA**	**KIR**	**Total**	**HLA**	**KIR**	**Total**
**Entropy**												
Mean ± SD	1.11 ± 1.40	60.15 ± 7.06		1.59 ± 1.94	61.22 ± 6.04		1.49 ± 2.01	59.84 ± 7.19		1.56 ± 1.96	60.81 ± 6.42	
Median (IQR)	0.66 (1.06)	63.25 (5.35)		0.99 (1.36)	63.25 (4.47)		0.69 (1.13)	63.25 (3.59)		0.92 (1.29)	63.25 (5.35)	
Variance	1.95	49.86		3.77	36.48		4.03	51.64		3.83	41.25	
**Entropy ratio**												
Mean ± SD	1.00 ± 1.26	1.00 ± 0.12	1.00 ± 0.63	1.44 ± 1.75	1.02 ± 0.10	1.23 ± 0.87	1.35 ± 1.82	0.99 ± 0.12	1.17 ± 0.90	1.41 ± 1.77	1.01 ± 0.11	1.21 ± 0.88
Median (IQR)	0.60 (0.96)	1.05 (0.09)	0.80 (0.47)	0.90 (1.23)	1.05 (0.07)	0.96 (0.62)	0.62 (1.02)	1.05 (0.06)	0.84 (0.50)	0.83 (1.17)	1.05 (0.09)	0.93 (0.60)
Variance	1.59	0.014	0.40	3.08	0.010	0.76	3.30	0.014	0.81	3.13	0.011	0.77
