# Supplementary material for: Entropy of human leukocyte antigen and killer-cell immunoglobulin-like receptor systems in immune-mediated disorders: A pilot study on multiple sclerosis
Source: PLoS One. 2019 Dec 17;14(12):e0226615. doi: 10.1371/journal.pone.0226615 (PMC6917289; doi:10.1371/journal.pone.0226615)
Supplement: S5 File — (PDF) [file pone.0226615.s005.pdf]

S5 File. Cochran's rule

The expected frequency e_{ij} for each ij-cell of a contingency table can be computed through the formula $e_{ij} = \frac{\text{row}^{(i)} \times \text{col}^{(j)}}{\text{grand total}}$,where $\text{row}^{(i)}$ is the marginal total of the elements on the i-th row, $\text{col}^{(j)}$ is the marginal total of the elements on the j-th column while the "grand total" is the sum of all row (or column) marginal totals.

Cochran's rule[1] claims that for contingency tables with more than one degree of freedom the use of χ^2 distribution as an adequate approximation to Pearson's X^2 statistics is possible when all the expected frequencies are greater than 1 and at least 80% of them are greater than 5. Cochran's rule is often quoted in a more conservative version[2]: "For tables with more than a single degree of freedom, a minimum expected frequency of 5 can be regarded as adequate".

In our study, the number of distinct HLA four-loci haplotypes observed in both the groups of healthy controls and RRMS patients was 792. In order to reduce the number of HLA haplotypes reported in a Table, we applied the condition required by Cochran's rule and only considered the HLA haplotypes with expected frequencies greater than 5.

If $N_{i;\,ctr}$ and $N_{i;\,RR}$ are the number of times the ith HLA haplotype is present in the group of controls and RRMS patients, respectively, $N_{ctr} = 619$ is the number of healthy controls, $N_{RR} = 189$ is the number of RRMS patients, $N_{tot} = N_{ctr} + N_{RR} = 808$ and $N_i = N_{i;\,ctr} + N_{i;\,RR}$, we obtain the results shown in the following table.

	**HLA haplotype**		
	**Present**	**Absent**	
**Healthy controls**	$N_{i;\,ctr}$	$N_{ctr} - N_{i;\,ctr}$	N_{ctr}
**RRMS patients**	$N_{i;\,RR}$	$N_{RR} - N_{i;\,RR}$	N_{RR}
	N_i	$N_{tot} - N_i$	N_{tot}

Expected → **frequencies**

	**HLA haplotype**	
	**Present**	**Absent**
**Healthy controls**	$\frac{N_{ctr} \cdot N_i}{N_{tot}}$	$\frac{N_{ctr} \cdot (N_{tot} - N_{i;ctr})}{N_{tot}}$
**RRMS patients**	$\frac{N_{RR} \cdot N_i}{N_{tot}}$	$\frac{N_{RR} \cdot (N_{tot} - N_{i;RR})}{N_{tot}}$

[1]Cochran, W. G. (1952), "The χ^2 Test of Goodness of Fit," Annals of Mathematical Statistics, 23, 315–345.Cochran, W. G. (1954), "Some Methods for Strengthening the Common χ^2 Tests," Biometrics, 10, 417–451.

[2] Hays, W. L. (1973), Statistics for the Social Sciences, New York: Holt, Rinehart and Winston: p. 736.

According to the data in our study, the minimum expected frequency was $\frac{N_{RR} \cdot N_i}{N_{tot}}$, associated to the number $N_{i;\,RR}$ of RRMS patients with the ith HLA haplotype.

By applying the Cochran's rule, we finally obtained: $N_i > 5 \cdot \frac{N_{tot}}{N_{RR}} = 21.4$, i.e. only the HLA haplotypes present in at least 22 subjects, either RRMS patients or healthy controls, were considered.

An analogous conclusion can be drawn for the KIR haplotypes: only 9 of the 38 KIR haplotypes in either group of healthy controls or RRMS patients satisfied the Cochran's requirement.
