# Supplementary material for: Entropy of human leukocyte antigen and killer-cell immunoglobulin-like receptor systems in immune-mediated disorders: A pilot study on multiple sclerosis
Source: PLoS One. 2019 Dec 17;14(12):e0226615. doi: 10.1371/journal.pone.0226615 (PMC6917289; doi:10.1371/journal.pone.0226615)
Supplement: S1 Table — (PDF) [file pone.0226615.s006.pdf]

S1 Table. HLA alleles and haplotypes in patient and control cohorts

The HLA allele frequencies in a cohort of 619 Sardinian healthy controls were compared to those observed in a group of 270 patients affected by multiple sclerosis (MS), stratified into a group of 81 patients with primary progressive multiple sclerosis (PPMS) and a group of 189 patients with relapsing remitting multiple sclerosis (RRMS).

For multiple comparisons between healthy controls and each group of patients, the corrected P values (P_c) were obtained by multiplying the P values calculated according to the two-tailed Fisher's exact test by the number of tested alleles (17 for HLA-A, 27 for HLA-B, 13 for HLA-C and 12 for HLA-DR).

The only alleles with $P_c < 0.05$, corresponding to statistically significant comparisons, were:

A*30 ($P_c = 6.1 \cdot 10^{-4}$ for RRMS patients and $P_c = 5.3 \cdot 10^{-4}$ for MS patients);

B*18 ($P_c = 7.3 \cdot 10^{-4}$ for RRMS patients and $P_c = 4.9 \cdot 10^{-4}$ for MS patients);

B*58 ($P_c = 0.019$ for MS patients);

C*05 ($P_c = 0.002$ for RRMS patients and $P_c = 0.003$ for MS patients);

C*06 ($P_c = 0.039$ for RRMS patients);

DR*03 ($P_c = 6.2 \cdot 10^{-4}$ for RRMS patients and $P_c = 9.2 \cdot 10^{-4}$ for MS patients);

DR*04 ($P_c = 0.044$ for MS patients);

DR*16 ($P_c = 0.006$ for RRMS patients and $P_c = 0.003$ for MS patients).

HLA-A locus	Controls (1238 alleles)		RRMS patients (378 alleles)		PPMS patients (162 alleles)		MS patients (540 alleles)	
	n	f (%)	n	f (%)	n	f (%)	n	f (%)
**A*01**	102	8.24	24	6.35	6	3.70	30	5.56
**A*02**	363	29.32	94	24.87	37	22.84	131	24.26
**A*03**	65	5.25	14	3.70	12	7.41	26	4.81
**A*11**	81	6.54	27	7.14	15	9.26	42	7.78
**A*23**	20	1.62	5	1.32	5	3.09	10	1.85
**A*24**	119	9.61	26	6.88	10	6.17	36	6.67
**A*25**	2	0.16	2	0.53	-	-	2	0.37
**A*26**	28	2.26	9	2.38	4	2.47	13	2.41
**A*29**	26	2.10	8	2.12	2	1.23	10	1.85
**A*30**	230	18.58	109	28.84	40	24.69	149	27.59
**A*31**	14	1.13	8	2.12	5	3.09	13	2.41
**A*32**	108	8.72	33	8.73	21	12.96	54	10.00
**A*33**	45	3.63	9	2.38	3	1.85	12	2.22

**A*66**	1	0.08	1	0.26	-	-	1	0.19
**A*68**	19	1.53	4	1.06	1	0.62	5	0.93
**A*69**	10	0.81	2	0.53	1	0.62	3	0.56
**A*74**	5	0.40	1	0.26	-	-	1	0.19

**HLA-B locus**	**Controls (1238 alleles)**		**RRMS patients (378 alleles)**		**PPMS patients (162 alleles)**		**MS patients (540 alleles)**	
	**n**	**f (%)**	**n**	**f (%)**	**n**	**f (%)**	**n**	**f (%)**
**B*07**	37	2.99	6	1.59	8	4.94	14	2.59
**B*08**	30	2.42	10	2.65	3	1.85	13	2.41
**B*13**	23	1.86	9	2.38	-	-	9	1.67
**B*14**	74	5.98	13	3.44	4	2.47	17	3.15
**B*15**	23	1.86	3	0.79	1	0.62	4	0.74
**B*18**	315	25.44	139	36.77	53	32.72	192	35.56
**B*27**	24	1.94	8	2.12	6	3.70	14	2.59
**B*35**	153	12.36	42	11.11	20	12.35	62	11.48
**B*37**	14	1.13	11	2.91	5	3.09	16	2.96
**B*38**	16	1.29	-	-	1	0.62	1	0.19
**B*39**	22	1.78	9	2.38	5	3.09	14	2.59
**B*40**	11	0.89	2	0.53	3	1.85	5	0.93
**B*41**	18	1.45	2	0.53	-	-	2	0.37
**B*44**	58	4.68	10	2.65	9	5.56	19	3.52
**B*45**	18	1.45	11	2.91	2	1.23	13	2.41
**B*47**	2	0.16	1	0.26	-	-	1	0.19
**B*49**	75	6.06	24	6.35	11	6.79	35	6.48
**B*50**	15	1.21	13	3.44	4	2.47	17	3.15
**B*51**	79	6.38	22	5.82	6	3.70	28	5.19
**B*52**	21	1.70	3	0.79	1	0.62	4	0.74
**B*53**	5	0.40	2	0.53	2	1.23	4	0.74
**B*55**	40	3.23	10	2.65	5	3.09	15	2.78
**B*56**	3	0.24	-	-	1	0.62	1	0.19
**B*57**	9	0.73	-	-	2	1.23	2	0.37
**B*58**	141	11.39	24	6.35	10	6.17	34	6.3
**B*73**	8	0.65	2	0.53	-	-	2	0.37
**B*78**	4	0.32	2	0.53	-	-	2	0.37

**HLA-C locus**	**Controls (1238 alleles)**		**RRMS patients (378 alleles)**		**PPMS patients (162 alleles)**		**MS patients (540 alleles)**	
	**n**	**f (%)**	**n**	**f (%)**	**n**	**f (%)**	**n**	**f (%)**
**C*01**	26	2.10	6	1.59	7	4.32	13	2.41
**C*02**	73	5.90	18	4.76	14	8.64	32	5.93
**C*03**	48	3.88	14	3.70	6	3.70	20	3.70
**C*04**	139	11.23	44	11.64	17	10.49	61	11.30
**C*05**	243	19.63	110	29.10	39	24.07	149	27.59
**C*06**	76	6.14	41	10.85	11	6.79	52	9.63
**C*07**	369	29.81	88	23.28	38	23.46	126	23.33
**C*08**	73	5.90	17	4.50	5	3.09	22	4.07
**C*12**	77	6.22	16	4.23	14	8.64	30	5.56
**C*14**	10	0.81	6	1.59	2	1.23	8	1.48
**C*15**	54	4.36	11	2.91	6	3.70	17	3.15
**C*16**	34	2.75	5	1.32	3	1.85	8	1.48
**C*17**	16	1.29	2	0.53	-	-	2	0.37

HLA-DR locus	Controls (1238 alleles)		RRMS patients (378 alleles)		PPMS patients (162 alleles)		MS patients (540 alleles)	
	n	f (%)	n	f (%)	n	f (%)	n	f (%)
**DR*01**	104	8.40	23	6.08	10	6.17	33	6.11
**DR*03**	272	21.97	123	32.54	44	27.16	167	30.93
**DR*04**	164	13.25	69	18.25	32	19.75	101	18.70
**DR*07**	68	5.49	20	5.29	10	6.17	30	5.56
**DR*08**	23	1.86	5	1.32	2	1.23	7	1.30
**DR*10**	23	1.86	8	2.12	2	1.23	10	1.85
**DR*11**	195	15.75	47	12.43	24	14.81	71	13.15
**DR*12**	19	1.53	4	1.06	4	2.47	8	1.48
**DR*13**	51	4.12	12	3.17	4	2.47	16	2.96
**DR*14**	34	2.75	6	1.59	1	0.62	7	1.30
**DR*15**	43	3.47	17	4.50	6	3.70	23	4.26
**DR*16**	240	19.39	44	11.64	23	14.20	67	12.41

The distinct HLA four-loci haplotypes common to both groups of RRMS patients and healthy controls were 792. Among these, the HLA haplotypes satisfying the Cochran's rule (with expected frequencies greater than 5) were 30 and are listed in the Table below.

HLA haplotypes				Controls (1238 haplotypes)		RRMS patients (378 haplotypes)	
				n	f (%)	n	f (%)
A*30	B*18	C*05	DR*03	154	12.44	78	20.63
A*02	B*58	C*07	DR*16	82	6.62	10	2.65
A*02	B*18	C*05	DR*03	69	5.57	32	8.47
A*30	B*18	C*07	DR*03	42	3.39	21	5.56
A*30	B*18	C*05	DR*16	36	2.91	10	2.65
A*30	B*18	C*05	DR*11	34	2.75	12	3.17
A*02	B*18	C*07	DR*16	33	2.67	8	2.12
A*02	B*18	C*07	DR*03	30	2.42	10	2.65
A*02	B*49	C*07	DR*04	30	2.42	9	2.38
A*02	B*58	C*07	DR*03	30	2.42	8	2.12
A*02	B*18	C*07	DR*11	26	2.10	3	0.79
A*02	B*18	C*05	DR*16	24	1.94	6	1.59
A*02	B*58	C*07	DR*04	24	1.94	3	0.79
A*02	B*18	C*07	DR*04	22	1.78	10	2.65
A*32	B*18	C*07	DR*16	22	1.78	7	1.85
A*01	B*49	C*07	DR*04	21	1.70	14	3.70
A*30	B*35	C*05	DR*03	21	1.70	6	1.59
A*32	B*18	C*05	DR*03	21	1.70	11	2.91
A*02	B*35	C*04	DR*16	20	1.62	5	1.32
A*24	B*18	C*05	DR*03	20	1.62	11	2.91
A*30	B*18	C*07	DR*16	20	1.62	7	1.85
A*33	B*14	C*08	DR*01	20	1.62	3	0.79
A*01	B*08	C*07	DR*03	19	1.53	3	0.79
A*02	B*35	C*04	DR*11	18	1.45	7	1.85
A*30	B*18	C*04	DR*03	18	1.45	10	2.65
A*30	B*18	C*05	DR*04	18	1.45	19	5.03

A*30	B*58	C*07	DR*16	18	1.45	7	1.85
A*02	B*14	C*08	DR*01	17	1.37	6	1.59
A*01	B*18	C*05	DR*03	16	1.29	6	1.59
A*30	B*18	C*07	DR*04	12	0.97	11	2.91

For multiple comparisons between the two groups, the corrected P values (P_c) were computed by multiplying the P values obtained according to the two-tailed Fisher's exact test by the number of tested HLA four-loci haplotypes. P_c values smaller than 0.05 were only obtained for the extended haplotype HLA-A*30, B*18, C*05, DR*03 (P_c = 0.004), which is the most frequent haplotype in the Sardinian population, and for the haplotype HLA-A*30, B*18, C*05, DR*04 (P_c = 0.006).

An analogous comparison between healthy controls and MS patients yielded statistically significant differences only for the aforementioned extended haplotype (P_c = 0.023).
