# Supplementary material for: Entropy of human leukocyte antigen and killer-cell immunoglobulin-like receptor systems in immune-mediated disorders: A pilot study on multiple sclerosis
Source: PLoS One. 2019 Dec 17;14(12):e0226615. doi: 10.1371/journal.pone.0226615 (PMC6917289; doi:10.1371/journal.pone.0226615)
Supplement: S2 Table — (PDF) [file pone.0226615.s007.pdf]

S2 Table. KIR genes and haplotypes in patient and control cohorts

The frequencies of activating and inhibitory KIR genes in a cohort of 619 Sardinian healthy controls were compared to those found in a group of 270 patients affected by multiple sclerosis (MS), the latter stratified into a group of 81 patients with primary progressive multiple sclerosis (PPMS) and a group of 189 patients with relapsing remitting multiple sclerosis (RRMS). All the P values, calculated according to the two-tailed Fisher's exact test, were greater than 0.05, therefore no comparison was statistically significant.

KIR genes	Controls (619)		RRMS patients (189)		PPMS patients (81)		MS patients (270)	
	n	f (%)	n	f (%)	n	f (%)	n	f (%)
KIR2DL1	594	96.0	179	94.7	76	93.8	255	94.4
KIR2DL2	358	57.8	113	59.8	41	50.6	154	57.0
KIR2DL3	533	86.1	172	91.0	70	86.4	242	89.6
KIR2DL4	619	100.0	189	100.0	81	100.0	270	100.0
KIR2DL5	344	55.6	112	59.3	44	54.3	156	57.8
KIR3DL1	582	94.0	182	96.3	74	91.4	256	94.8
KIR3DL2	619	100.0	189	100.0	81	100.0	270	100.0
KIR3DL3	619	100.0	189	100.0	81	100.0	270	100.0
KIR2DS1	238	38.4	79	41.8	37	45.7	116	43.0
KIR2DS2	359	58.0	114	60.3	42	51.9	156	57.8
KIR2DS3	224	36.2	66	34.9	23	28.4	89	33.0
KIR2DS4	582	94.0	182	96.3	74	91.4	256	94.8
KIR2DS5	196	31.7	60	31.7	32	39.5	92	34.1
KIR3DS1	230	37.2	76	40.2	33	40.7	109	40.4

The presence or absence (1/0) of KIR genes in either group of RRMS patients or healthy controls generated 38 distinct KIR haplotypes. Among these, the KIR haplotypes satisfying the Cochran's rule (with expected frequencies greater than 5) were only 9 and are listed in the Table below. Comparisons of the KIR haplotypes in RRMS patients and controls were performed using the two-tailed Fisher's exact test. All P values were greater than 0.05. Therefore, no comparison was statistically significant.

**KIR gene haplotypes**	***2DL1***	***2DL2***	***2DL3***	***2DL4***	***2DL5***	***3DL1***	***3DL1***	***3DL1***	***2DS1***	***2DS2***	***2DS3***	***2DS4***	***2DS5***	***3DS1***	**Controls (619)**		**RRMS patients (189)**	
															**n**	**f (%)**	**n**	**f (%)**
1	1	0	1	1	0	1	1	1	0	0	0	1	0	0	175	28.27	47	24.87
2	1	1	1	1	0	1	1	1	0	1	0	1	0	0	81	13.09	23	12.17
3	1	1	1	1	1	1	1	1	0	1	1	1	0	0	69	11.15	27	14.29
4	1	0	1	1	1	1	1	1	1	0	0	1	1	1	50	8.08	17	9.00
5	1	1	1	1	1	1	1	1	1	1	1	1	1	1	37	5.98	11	5.82
6	1	1	1	1	1	1	1	1	1	1	0	1	1	1	35	5.65	16	8.47
7	1	1	0	1	1	1	1	1	0	1	1	1	0	0	28	4.52	3	1.59
8	1	0	1	1	1	1	1	1	1	0	1	1	0	1	18	2.91	6	3.18
9	1	1	1	1	1	1	1	1	1	1	1	1	0	1	15	2.42	8	4.23

The next Table reports the frequencies of the 24 distinct couples of inhibitory KIR genes *KIR2DL2, KIR2DL3, KIR2DL5, KIR3DL1* (excluding those with percentages higher than 90%) in the groups of RRMS patients and healthy controls. The corrected P values (P_c) were obtained by multiplying the P values, computed using the two-tailed Fisher's exact test, by the number of tested KIR gene couples. All P_c values were greater than 0.05; therefore, no comparison was statistically significant.

**KIR gene couples**	**Presence or absence**	**Controls**	**RRMS patients**

				n	f (%)	n	f (%)
2DL2	*2DL3*	0	0	0	0	0	0
2DL2	*2DL3*	0	1	261	42.16	76	40.21
2DL2	*2DL3*	1	0	86	13.89	17	9.00
2DL2	*2DL3*	1	1	272	43.94	96	50.79
2DL2	*2DL5*	0	0	182	29.40	50	26.46
2DL2	*2DL5*	0	1	79	12.76	26	13.76
2DL2	*2DL5*	1	0	93	15.02	27	14.29
2DL2	*2DL5*	1	1	265	42.81	86	45.50
2DL2	*3DL1*	0	0	8	1.29	2	1.06
2DL2	*3DL1*	0	1	253	40.87	74	39.15
2DL2	*3DL1*	1	0	29	4.68	5	2.65
2DL2	*3DL1*	1	1	329	53.15	108	57.14
2DL3	*2DL5*	0	0	11	1.78	4	2.12
2DL3	*2DL5*	0	1	75	12.12	13	6.88
2DL3	*2DL5*	1	0	264	42.65	73	38.62
2DL3	*2DL5*	1	1	269	43.46	99	52.38
2DL3	*3DL1*	0	0	15	2.42	2	1.06
2DL3	*3DL1*	0	1	71	11.47	15	7.94
2DL3	*3DL1*	1	0	22	3.55	5	2.65
2DL3	*3DL1*	1	1	511	82.55	167	88.36
2DL5	*3DL1*	0	0	0	0	0	0
2DL5	*3DL1*	0	1	275	44.43	77	40.74
2DL5	*3DL1*	1	0	37	5.98	7	3.70
2DL5	*3DL1*	1	1	307	49.60	105	55.56
