# Supplementary material for: Entropy of human leukocyte antigen and killer-cell immunoglobulin-like receptor systems in immune-mediated disorders: A pilot study on multiple sclerosis
Source: PLoS One. 2019 Dec 17;14(12):e0226615. doi: 10.1371/journal.pone.0226615 (PMC6917289; doi:10.1371/journal.pone.0226615)
Supplement: S3 Table — (PDF) [file pone.0226615.s008.pdf]

S3 Table. Frequencies and entropies of KIR gene couples and HLA haplotypes

The following Tables provide the frequencies and entropies of each couple of inhibitory KIR genes and each HLA four-loci haplotype observed in the control group. These data were used for the evaluation of HLA and KIR entropy in each patient, according to the procedure discussed in the **S1 File**.

The Table on this page lists the frequencies and the corresponding KIR entropies of the 24 distinct couples of inhibitory KIR genes *KIR2DL2, KIR2DL3, KIR2DL5, KIR3DL1* (excluding those with percentages higher than 90%) in the group of 619 controls.

KIR gene couples		Frequency	Entropy
(*2DL2*, *2DL3*)	(0, 0)	0	0
(*2DL2*, *2DL3*)	(0, 1)	0.4216	68.08
(*2DL2*, *2DL3*)	(1, 0)	0.1389	40.3
(*2DL2*, *2DL3*)	(1, 1)	0.4394	68.58
(*2DL2*, *2DL5*)	(0, 0)	0.294	60.57
(*2DL2*, *2DL5*)	(0, 1)	0.1276	38.19
(*2DL2*, *2DL5*)	(1, 0)	0.1502	42.31
(*2DL2*, *2DL5*)	(1, 1)	0.4281	68.28
(*2DL2*, *3DL1*)	(0, 0)	0.0129	6.9
(*2DL2*, *3DL1*)	(0, 1)	0.4087	67.64
(*2DL2*, *3DL1*)	(1, 0)	0.0468	18.91
(*2DL2*, *3DL1*)	(1, 1)	0.5315	69.12
(*2DL3*, *2DL5*)	(0, 0)	0.0178	8.92
(*2DL3*, *2DL5*)	(0, 1)	0.1212	36.92
(*2DL3*, *2DL5*)	(1, 0)	0.4265	68.23
(*2DL3*, *2DL5*)	(1, 1)	0.4346	68.46
(*2DL3*, *3DL1*)	(0, 0)	0.0242	11.41
(*2DL3*, *3DL1*)	(0, 1)	0.1147	35.62
(*2DL3*, *3DL1*)	(1, 0)	0.0355	15.35
(*2DL3*, *3DL1*)	(1, 1)	0.8255	46.29
(*2DL5*, *3DL1*)	(0, 0)	0	0
(*2DL5*, *3DL1*)	(0, 1)	0.4443	68.69
(*2DL5*, *3DL1*)	(1, 0)	0.0598	22.63
(*2DL5*, *3DL1*)	(1, 1)	0.496	69.31

The total number of different HLA four-loci haplotypes in the group of 619 controls was 3895. The Table on the following pages lists the frequencies f calculated on the total number of 7612 HLA haplotypes present in the control group and the corresponding HLA entropies S.

A	B	C	DR	*f*	*S*
1	7	7	1	0.00013	0.129
1	7	7	4	0.00013	0.129
1	7	7	7	0.00026	0.241
1	7	7	8	0.00013	0.129
1	7	7	15	0.00039	0.345
1	7	7	16	0.00026	0.241
1	7	12	4	0.00013	0.129
1	7	12	8	0.00013	0.129
1	7	12	16	0.00026	0.241
1	7	16	1	0.00013	0.129
1	7	16	15	0.00013	0.129
1	8	3	3	0.00026	0.241
1	8	3	4	0.00013	0.129
1	8	3	11	0.00013	0.129
1	8	3	14	0.00026	0.241
1	8	4	3	0.00013	0.129
1	8	4	7	0.00013	0.129
1	8	4	16	0.00013	0.129
1	8	5	3	0.00066	0.549
1	8	5	8	0.00013	0.129
1	8	5	11	0.00013	0.129
1	8	5	16	0.00013	0.129
1	8	6	3	0.00013	0.129
1	8	6	7	0.00013	0.129
1	8	7	1	0.00013	0.129
1	8	7	3	0.0025	1.748
1	8	7	4	0.00039	0.345
1	8	7	7	0.00026	0.241
1	8	7	8	0.00013	0.129
1	8	7	11	0.00026	0.241
1	8	7	13	0.00013	0.129
1	8	7	14	0.00026	0.241
1	8	7	16	0.00079	0.643
1	8	12	1	0.00013	0.129
1	8	12	3	0.00026	0.241
1	8	12	16	0.00013	0.129
1	8	15	3	0.00026	0.241
1	8	15	4	0.00013	0.129
1	8	15	16	0.00013	0.129
1	13	4	11	0.00013	0.129
1	13	4	15	0.00013	0.129
1	13	6	3	0.00026	0.241
1	13	6	4	0.00026	0.241
1	13	6	7	0.00053	0.453
1	13	7	3	0.00026	0.241
1	13	7	4	0.00026	0.241
1	13	7	7	0.00053	0.453
1	14	3	1	0.00013	0.129
1	14	3	14	0.00013	0.129
1	14	4	1	0.00013	0.129
1	14	4	4	0.00013	0.129
1	14	6	1	0.00013	0.129
1	14	6	3	0.00013	0.129
1	14	7	1	0.00013	0.129
1	14	7	3	0.00013	0.129
1	14	7	4	0.00013	0.129
1	14	7	13	0.00013	0.129
1	14	8	1	0.00079	0.643
1	14	8	3	0.00039	0.345
1	14	8	4	0.00026	0.241
1	14	8	13	0.00026	0.241
1	14	8	14	0.00026	0.241
1	14	8	15	0.00013	0.129
1	14	12	1	0.00013	0.129
1	14	12	3	0.00013	0.129
1	14	12	14	0.00013	0.129
1	14	12	15	0.00013	0.129
1	14	17	1	0.00013	0.129
1	14	17	13	0.00013	0.129
1	15	3	3	0.00026	0.241
1	15	3	11	0.00026	0.241
1	15	3	13	0.00013	0.129
1	15	3	16	0.00026	0.241
1	15	4	1	0.00013	0.129
1	15	4	4	0.00013	0.129
1	15	5	3	0.00026	0.241
1	15	5	13	0.00013	0.129
1	15	5	16	0.00013	0.129
1	15	7	1	0.00013	0.129
1	15	7	3	0.00039	0.345
1	15	7	7	0.00013	0.129
1	15	7	11	0.00026	0.241
1	15	7	13	0.00053	0.453
1	15	7	16	0.00013	0.129
1	15	8	1	0.00026	0.241
1	15	8	4	0.00013	0.129
1	15	8	13	0.00013	0.129
1	15	12	7	0.00013	0.129
1	15	12	11	0.00026	0.241
1	18	3	3	0.00013	0.129
1	18	3	11	0.00013	0.129
1	18	3	16	0.00013	0.129
1	18	4	7	0.00013	0.129
1	18	4	10	0.00013	0.129
1	18	4	11	0.00013	0.129
1	18	4	16	0.00013	0.129
1	18	5	3	0.0021	1.505
1	18	5	4	0.00053	0.453
1	18	5	8	0.00013	0.129
1	18	5	11	0.00026	0.241
1	18	5	13	0.00013	0.129
1	18	5	16	0.00066	0.549
1	18	6	3	0.00013	0.129
1	18	6	16	0.00013	0.129
1	18	7	1	0.00026	0.241
1	18	7	3	0.00197	1.424
1	18	7	4	0.00066	0.549
1	18	7	7	0.00013	0.129
1	18	7	8	0.00013	0.129
1	18	7	10	0.00013	0.129
1	18	7	11	0.00053	0.453
1	18	7	12	0.00026	0.241
1	18	7	13	0.00026	0.241
1	18	7	15	0.00026	0.241
1	18	7	16	0.00066	0.549
1	18	12	4	0.00013	0.129
1	18	12	7	0.00026	0.241
1	18	12	11	0.00053	0.453
1	18	12	12	0.00013	0.129
1	18	12	15	0.00026	0.241
1	18	12	16	0.00013	0.129
1	18	15	1	0.00013	0.129
1	18	15	3	0.00013	0.129
1	18	16	1	0.00013	0.129
1	18	16	15	0.00013	0.129
1	27	1	12	0.00013	0.129
1	27	1	15	0.00013	0.129
1	27	12	12	0.00013	0.129
1	27	12	15	0.00013	0.129
1	35	1	1	0.00013	0.129
1	35	1	11	0.00013	0.129
1	35	4	1	0.00066	0.549
1	35	4	3	0.00053	0.453
1	35	4	4	0.00039	0.345
1	35	4	7	0.00026	0.241
1	35	4	8	0.00013	0.129
1	35	4	10	0.00026	0.241
1	35	4	11	0.00092	0.735
1	35	4	12	0.00013	0.129
1	35	4	13	0.00013	0.129
1	35	4	14	0.00053	0.453
1	35	4	15	0.00039	0.345
1	35	4	16	0.00066	0.549
1	35	5	1	0.00013	0.129
1	35	5	13	0.00013	0.129
1	35	6	10	0.00013	0.129
1	35	6	11	0.00013	0.129
1	35	6	14	0.00026	0.241
1	35	7	1	0.00026	0.241
1	35	7	3	0.00039	0.345
1	35	7	4	0.00039	0.345
1	35	7	7	0.00013	0.129
1	35	7	8	0.00013	0.129
1	35	7	10	0.00013	0.129
1	35	7	11	0.00026	0.241
1	35	7	12	0.00013	0.129
1	35	7	14	0.00013	0.129
1	35	7	15	0.00013	0.129
1	35	7	16	0.00066	0.549
1	35	12	3	0.00013	0.129
1	35	12	4	0.00013	0.129
1	35	12	7	0.00013	0.129
1	35	12	11	0.00026	0.241
1	35	12	14	0.00013	0.129
1	35	12	15	0.00013	0.129
1	35	12	16	0.00013	0.129
1	35	16	4	0.00013	0.129
1	35	16	16	0.00013	0.129
1	37	4	10	0.00013	0.129
1	37	4	14	0.00013	0.129
1	37	5	3	0.00013	0.129
1	37	5	16	0.00013	0.129
1	37	6	3	0.00013	0.129
1	37	6	10	0.00026	0.241
1	37	6	14	0.00013	0.129
1	37	6	15	0.00013	0.129
1	37	6	16	0.00013	0.129
1	37	12	10	0.00013	0.129
1	37	12	15	0.00013	0.129
1	39	4	11	0.00013	0.129
1	39	4	15	0.00013	0.129
1	39	7	1	0.00013	0.129
1	39	7	3	0.00039	0.345
1	39	7	8	0.00013	0.129
1	39	7	11	0.00013	0.129
1	39	7	13	0.00013	0.129
1	39	7	15	0.00013	0.129
1	39	7	16	0.00026	0.241
1	39	12	1	0.00013	0.129
1	39	12	3	0.00026	0.241
1	39	12	8	0.00013	0.129
1	39	12	16	0.00026	0.241
1	41	7	13	0.00013	0.129
1	41	7	16	0.00026	0.241
1	41	8	1	0.00013	0.129
1	41	8	13	0.00013	0.129
1	41	17	1	0.00013	0.129
1	41	17	13	0.00026	0.241
1	41	17	16	0.00026	0.241
1	44	2	14	0.00013	0.129
1	44	2	16	0.00013	0.129
1	44	4	1	0.00013	0.129
1	44	4	13	0.00013	0.129
1	44	5	1	0.00013	0.129
1	44	5	4	0.00026	0.241
1	44	5	12	0.00013	0.129
1	44	5	13	0.00013	0.129
1	44	5	16	0.00013	0.129
1	44	6	4	0.00013	0.129
1	44	6	7	0.00013	0.129
1	44	6	16	0.00013	0.129
1	44	7	4	0.00013	0.129
1	44	7	12	0.00013	0.129
1	44	7	14	0.00013	0.129
1	44	7	16	0.00013	0.129
1	44	16	7	0.00013	0.129
1	45	6	4	0.00013	0.129
1	45	6	15	0.00013	0.129
1	45	7	4	0.00013	0.129
1	45	7	15	0.00013	0.129
1	47	6	4	0.00013	0.129
1	47	6	13	0.00013	0.129
1	47	7	4	0.00013	0.129
1	47	7	13	0.00013	0.129
1	49	1	11	0.00013	0.129
1	49	4	3	0.00013	0.129
1	49	4	4	0.00026	0.241
1	49	4	12	0.00013	0.129
1	49	4	14	0.00013	0.129
1	49	4	16	0.00013	0.129
1	49	5	3	0.00039	0.345
1	49	5	4	0.00053	0.453
1	49	5	12	0.00013	0.129
1	49	6	4	0.00066	0.549
1	49	6	7	0.00026	0.241
1	49	6	13	0.00013	0.129
1	49	6	15	0.00013	0.129
1	49	7	3	0.00105	0.825
1	49	7	4	0.00276	1.902
1	49	7	7	0.00039	0.345
1	49	7	11	0.00039	0.345
1	49	7	12	0.00026	0.241
1	49	7	13	0.00013	0.129
1	49	7	14	0.00013	0.129
1	49	7	15	0.00026	0.241
1	49	7	16	0.00092	0.735
1	49	8	3	0.00013	0.129
1	49	8	4	0.00013	0.129
1	49	12	4	0.00013	0.129
1	49	12	11	0.00013	0.129
1	49	16	4	0.00013	0.129
1	49	16	16	0.00013	0.129
1	50	6	4	0.00013	0.129
1	50	7	4	0.00013	0.129
1	51	1	1	0.00026	0.241
1	51	1	11	0.00026	0.241
1	51	2	1	0.00013	0.129
1	51	2	11	0.00013	0.129
1	51	4	1	0.00013	0.129
1	51	4	11	0.00013	0.129
1	51	7	1	0.00013	0.129
1	51	7	3	0.00026	0.241
1	51	7	11	0.00013	0.129
1	51	7	16	0.00026	0.241
1	51	15	1	0.00013	0.129
1	51	15	3	0.00026	0.241
1	51	15	16	0.00013	0.129
1	52	1	12	0.00013	0.129
1	52	1	15	0.00013	0.129

1	52	3	14	0.00013	0.129
1	52	3	15	0.00013	0.129
1	52	4	3	0.00013	0.129
1	52	4	4	0.00013	0.129
1	52	4	11	0.00026	0.241
1	52	4	14	0.00013	0.129
1	52	4	15	0.00013	0.129
1	52	6	10	0.00013	0.129
1	52	6	15	0.00013	0.129
1	52	7	4	0.00013	0.129
1	52	7	12	0.00013	0.129
1	52	7	15	0.00013	0.129
1	52	7	16	0.00013	0.129
1	52	8	1	0.00013	0.129
1	52	8	3	0.00013	0.129
1	52	8	14	0.00013	0.129
1	52	8	15	0.00013	0.129
1	52	12	1	0.00013	0.129
1	52	12	3	0.00026	0.241
1	52	12	4	0.00026	0.241
1	52	12	10	0.00013	0.129
1	52	12	11	0.00039	0.345
1	52	12	12	0.00026	0.241
1	52	12	14	0.00039	0.345
1	52	12	15	0.00092	0.735
1	52	12	16	0.00013	0.129
1	55	3	1	0.00013	0.129
1	55	3	3	0.00013	0.129
1	55	3	4	0.00013	0.129
1	55	3	13	0.00013	0.129
1	55	3	14	0.00053	0.453
1	55	3	15	0.00013	0.129
1	55	3	16	0.00013	0.129
1	55	7	3	0.00013	0.129
1	55	7	4	0.00013	0.129
1	55	7	13	0.00013	0.129
1	55	7	14	0.00026	0.241
1	55	7	16	0.00013	0.129
1	55	8	1	0.00013	0.129
1	55	8	14	0.00013	0.129
1	55	12	14	0.00013	0.129
1	55	12	15	0.00013	0.129
1	56	1	11	0.00013	0.129
1	56	7	11	0.00013	0.129
1	57	4	11	0.00013	0.129
1	57	4	14	0.00013	0.129
1	57	5	4	0.00013	0.129
1	57	5	16	0.00013	0.129

1	57	6	1	0.00013	0.129
1	57	6	3	0.00013	0.129
1	57	6	4	0.00013	0.129
1	57	6	7	0.00013	0.129
1	57	6	11	0.00013	0.129
1	57	6	14	0.00013	0.129
1	57	6	16	0.00013	0.129
1	57	8	1	0.00013	0.129
1	57	8	3	0.00013	0.129
1	57	16	7	0.00013	0.129
1	58	2	14	0.00013	0.129
1	58	2	16	0.00013	0.129
1	58	4	1	0.00026	0.241
1	58	4	3	0.00013	0.129
1	58	4	8	0.00013	0.129
1	58	4	16	0.00026	0.241
1	58	5	3	0.00039	0.345
1	58	5	4	0.00013	0.129
1	58	5	16	0.00026	0.241
1	58	6	3	0.00013	0.129
1	58	6	7	0.00013	0.129
1	58	7	1	0.00026	0.241
1	58	7	3	0.00131	1.000
1	58	7	4	0.00092	0.735
1	58	7	7	0.00026	0.241
1	58	7	8	0.00013	0.129
1	58	7	12	0.00013	0.129
1	58	7	13	0.00013	0.129
1	58	7	14	0.00013	0.129
1	58	7	15	0.00013	0.129
1	58	7	16	0.00184	1.343
1	58	17	13	0.00013	0.129
1	58	17	16	0.00026	0.241
1	73	7	3	0.00013	0.129
1	73	7	4	0.00013	0.129
1	73	15	3	0.00013	0.129
1	73	15	4	0.00013	0.129
2	7	1	4	0.00013	0.129
2	7	1	11	0.00013	0.129
2	7	5	3	0.00026	0.241
2	7	5	4	0.00013	0.129
2	7	5	10	0.00013	0.129
2	7	7	1	0.00026	0.241
2	7	7	3	0.00013	0.129
2	7	7	4	0.00066	0.549
2	7	7	11	0.00026	0.241
2	7	7	14	0.00013	0.129
2	7	7	15	0.00039	0.345

2	7	7	16	0.00053	0.453
2	7	8	1	0.00013	0.129
2	7	8	3	0.00013	0.129
2	7	8	11	0.00013	0.129
2	7	8	15	0.00013	0.129
2	7	12	4	0.00013	0.129
2	7	12	16	0.00013	0.129
2	7	15	3	0.00039	0.345
2	7	15	4	0.00053	0.453
2	7	15	10	0.00013	0.129
2	7	15	11	0.00039	0.345
2	7	15	14	0.00013	0.129
2	7	15	15	0.00013	0.129
2	7	15	16	0.00013	0.129
2	8	2	3	0.00013	0.129
2	8	2	8	0.00026	0.241
2	8	2	11	0.00013	0.129
2	8	5	3	0.00013	0.129
2	8	5	8	0.00013	0.129
2	8	6	3	0.00013	0.129
2	8	6	4	0.00013	0.129
2	8	7	1	0.00026	0.241
2	8	7	3	0.00118	0.914
2	8	7	4	0.00026	0.241
2	8	7	8	0.00066	0.549
2	8	7	10	0.00013	0.129
2	8	7	11	0.00026	0.241
2	8	7	13	0.00026	0.241
2	8	7	15	0.00013	0.129
2	8	7	16	0.00039	0.345
2	8	8	1	0.00013	0.129
2	8	8	10	0.00013	0.129
2	8	12	1	0.00013	0.129
2	8	12	3	0.00013	0.129
2	8	15	3	0.00013	0.129
2	8	15	4	0.00013	0.129
2	8	15	16	0.00013	0.129
2	13	4	3	0.00013	0.129
2	13	4	7	0.00013	0.129
2	13	4	11	0.00013	0.129
2	13	4	15	0.00013	0.129
2	13	5	3	0.00026	0.241
2	13	5	7	0.00013	0.129
2	13	5	15	0.00013	0.129
2	13	6	1	0.00013	0.129
2	13	6	3	0.00026	0.241
2	13	6	4	0.00026	0.241
2	13	6	7	0.00053	0.453

2	13	6	11	0.00013	0.129
2	13	6	15	0.00013	0.129
2	13	6	16	0.00039	0.345
2	13	7	3	0.00026	0.241
2	13	7	4	0.00026	0.241
2	13	7	7	0.00039	0.345
2	13	7	16	0.00039	0.345
2	13	8	1	0.00013	0.129
2	13	8	15	0.00013	0.129
2	14	2	1	0.00066	0.549
2	14	2	3	0.00026	0.241
2	14	2	4	0.00013	0.129
2	14	2	7	0.00026	0.241
2	14	2	11	0.00039	0.345
2	14	2	16	0.00013	0.129
2	14	4	1	0.00013	0.129
2	14	4	4	0.00013	0.129
2	14	4	11	0.00013	0.129
2	14	4	15	0.00013	0.129
2	14	5	1	0.00026	0.241
2	14	5	3	0.00026	0.241
2	14	5	7	0.00013	0.129
2	14	5	16	0.00013	0.129
2	14	6	1	0.00026	0.241
2	14	6	7	0.00013	0.129
2	14	6	15	0.00013	0.129
2	14	7	1	0.00066	0.549
2	14	7	3	0.00013	0.129
2	14	7	4	0.00026	0.241
2	14	7	7	0.00026	0.241
2	14	7	10	0.00013	0.129
2	14	7	11	0.00013	0.129
2	14	7	15	0.00013	0.129
2	14	7	16	0.00039	0.345
2	14	8	1	0.00223	1.584
2	14	8	3	0.00079	0.643
2	14	8	4	0.00066	0.549
2	14	8	7	0.00092	0.735
2	14	8	10	0.00013	0.129
2	14	8	11	0.00118	0.914
2	14	8	13	0.00013	0.129
2	14	8	14	0.00013	0.129
2	14	8	15	0.00039	0.345
2	14	8	16	0.00079	0.643
2	14	12	1	0.00013	0.129
2	14	12	14	0.00013	0.129
2	14	14	1	0.00013	0.129
2	14	14	11	0.00013	0.129

2	14	14	13	0.00013	0.129
2	14	14	16	0.00013	0.129
2	14	15	3	0.00013	0.129
2	14	15	4	0.00013	0.129
2	14	15	11	0.00026	0.241
2	14	16	7	0.00013	0.129
2	14	16	11	0.00013	0.129
2	15	2	4	0.00026	0.241
2	15	2	11	0.00013	0.129
2	15	2	16	0.00013	0.129
2	15	3	4	0.00013	0.129
2	15	3	11	0.00013	0.129
2	15	4	4	0.00013	0.129
2	15	4	16	0.00013	0.129
2	15	5	3	0.00026	0.241
2	15	5	11	0.00013	0.129
2	15	5	13	0.00013	0.129
2	15	7	3	0.00026	0.241
2	15	7	4	0.00026	0.241
2	15	7	11	0.00026	0.241
2	15	7	13	0.00026	0.241
2	15	12	3	0.00013	0.129
2	15	12	4	0.00013	0.129
2	15	12	10	0.00013	0.129
2	15	12	11	0.00013	0.129
2	15	16	4	0.00013	0.129
2	15	16	10	0.00013	0.129
2	18	1	3	0.00013	0.129
2	18	1	4	0.00013	0.129
2	18	1	11	0.00039	0.345
2	18	1	13	0.00013	0.129
2	18	1	16	0.00013	0.129
2	18	2	1	0.00013	0.129
2	18	2	3	0.00079	0.643
2	18	2	7	0.00013	0.129
2	18	2	11	0.00039	0.345
2	18	2	13	0.00013	0.129
2	18	2	16	0.00092	0.735
2	18	3	3	0.00013	0.129
2	18	3	4	0.00013	0.129
2	18	3	13	0.00013	0.129
2	18	3	14	0.00013	0.129
2	18	3	16	0.00013	0.129
2	18	4	3	0.00105	0.825
2	18	4	4	0.00013	0.129
2	18	4	8	0.00013	0.129
2	18	4	11	0.00039	0.345
2	18	4	15	0.00026	0.241

2	18	4	16	0.00039	0.345
2	18	5	1	0.00053	0.453
2	18	5	3	0.00906	5.164
2	18	5	4	0.00145	1.093
2	18	5	7	0.00053	0.453
2	18	5	8	0.00079	0.643
2	18	5	10	0.00026	0.241
2	18	5	11	0.00197	1.424
2	18	5	12	0.00013	0.129
2	18	5	13	0.00131	1.000
2	18	5	15	0.00026	0.241
2	18	5	16	0.00315	2.129
2	18	6	3	0.00066	0.549
2	18	6	4	0.00039	0.345
2	18	6	11	0.00039	0.345
2	18	6	16	0.00026	0.241
2	18	7	1	0.00105	0.825
2	18	7	3	0.00394	2.575
2	18	7	4	0.00289	1.978
2	18	7	7	0.00053	0.453
2	18	7	8	0.00013	0.129
2	18	7	10	0.00013	0.129
2	18	7	11	0.00342	2.283
2	18	7	12	0.00039	0.345
2	18	7	13	0.00079	0.643
2	18	7	14	0.00013	0.129
2	18	7	15	0.00026	0.241
2	18	7	16	0.00434	2.794
2	18	8	1	0.00039	0.345
2	18	8	3	0.00026	0.241
2	18	8	4	0.00026	0.241
2	18	8	7	0.00039	0.345
2	18	8	11	0.00013	0.129
2	18	8	16	0.00013	0.129
2	18	12	1	0.00039	0.345
2	18	12	3	0.00066	0.549
2	18	12	4	0.00039	0.345
2	18	12	11	0.00145	1.093
2	18	12	12	0.00013	0.129
2	18	12	13	0.00013	0.129
2	18	12	15	0.00013	0.129
2	18	12	16	0.00066	0.549
2	18	14	3	0.00026	0.241
2	18	14	4	0.00039	0.345
2	18	14	11	0.00039	0.345
2	18	15	1	0.00039	0.345
2	18	15	3	0.00079	0.643
2	18	15	4	0.00066	0.549

2	18	15	10	0.00013	0.129
2	18	15	11	0.00066	0.549
2	18	15	16	0.00026	0.241
2	18	16	3	0.00039	0.345
2	18	16	11	0.00039	0.345
2	18	16	16	0.00013	0.129
2	18	17	3	0.00013	0.129
2	18	17	8	0.00026	0.241
2	18	17	13	0.00039	0.345
2	27	1	3	0.00013	0.129
2	27	1	12	0.00013	0.129
2	27	1	13	0.00013	0.129
2	27	1	16	0.00013	0.129
2	27	2	1	0.00053	0.453
2	27	2	3	0.00066	0.549
2	27	2	4	0.00026	0.241
2	27	2	8	0.00013	0.129
2	27	2	11	0.00013	0.129
2	27	2	15	0.00013	0.129
2	27	2	16	0.00053	0.453
2	27	4	1	0.00013	0.129
2	27	4	15	0.00013	0.129
2	27	5	3	0.00053	0.453
2	27	5	4	0.00013	0.129
2	27	5	11	0.00013	0.129
2	27	5	13	0.00013	0.129
2	27	5	16	0.00013	0.129
2	27	7	1	0.00026	0.241
2	27	7	3	0.00013	0.129
2	27	7	4	0.00026	0.241
2	27	7	8	0.00013	0.129
2	27	7	12	0.00013	0.129
2	27	7	16	0.00066	0.549
2	27	8	1	0.00013	0.129
2	27	8	3	0.00013	0.129
2	27	12	3	0.00013	0.129
2	27	12	11	0.00013	0.129
2	27	15	3	0.00013	0.129
2	27	15	11	0.00013	0.129
2	35	2	1	0.00026	0.241
2	35	2	15	0.00026	0.241
2	35	2	16	0.00013	0.129
2	35	3	1	0.00013	0.129
2	35	3	7	0.00013	0.129
2	35	3	8	0.00026	0.241
2	35	3	10	0.00013	0.129
2	35	3	11	0.00026	0.241
2	35	3	14	0.00026	0.241

2	35	3	16	0.00013	0.129
2	35	4	1	0.00092	0.735
2	35	4	3	0.00118	0.914
2	35	4	4	0.00079	0.643
2	35	4	7	0.00039	0.345
2	35	4	8	0.00039	0.345
2	35	4	11	0.00236	1.663
2	35	4	13	0.00039	0.345
2	35	4	14	0.00039	0.345
2	35	4	15	0.00079	0.643
2	35	4	16	0.00263	1.825
2	35	5	1	0.00013	0.129
2	35	5	3	0.00131	1.000
2	35	5	4	0.00013	0.129
2	35	5	8	0.00013	0.129
2	35	5	11	0.00039	0.345
2	35	5	13	0.00013	0.129
2	35	5	15	0.00013	0.129
2	35	5	16	0.00053	0.453
2	35	6	7	0.00013	0.129
2	35	6	11	0.00026	0.241
2	35	6	12	0.00013	0.129
2	35	6	14	0.00013	0.129
2	35	6	16	0.00013	0.129
2	35	7	1	0.00026	0.241
2	35	7	3	0.00013	0.129
2	35	7	4	0.00026	0.241
2	35	7	7	0.00039	0.345
2	35	7	10	0.00013	0.129
2	35	7	11	0.00066	0.549
2	35	7	12	0.00013	0.129
2	35	7	13	0.00026	0.241
2	35	7	16	0.00223	1.584
2	35	8	1	0.00013	0.129
2	35	8	4	0.00013	0.129
2	35	8	7	0.00013	0.129
2	35	8	11	0.00026	0.241
2	35	8	15	0.00013	0.129
2	35	12	11	0.00039	0.345
2	35	12	14	0.00013	0.129
2	35	12	15	0.00013	0.129
2	35	12	16	0.00039	0.345
2	35	14	11	0.00026	0.241
2	35	14	16	0.00026	0.241
2	35	15	1	0.00013	0.129
2	35	15	4	0.00013	0.129
2	35	15	11	0.00013	0.129
2	35	15	16	0.00053	0.453

2	35	16	3	0.00026	0.241
2	35	16	4	0.00013	0.129
2	35	16	7	0.00013	0.129
2	35	16	11	0.00039	0.345
2	35	16	16	0.00066	0.549
2	35	17	4	0.00013	0.129
2	35	17	11	0.00013	0.129
2	35	17	15	0.00013	0.129
2	35	17	16	0.00013	0.129
2	37	5	3	0.00026	0.241
2	37	5	11	0.00013	0.129
2	37	5	16	0.00026	0.241
2	37	6	3	0.00026	0.241
2	37	6	10	0.00013	0.129
2	37	6	11	0.00026	0.241
2	37	6	12	0.00026	0.241
2	37	6	16	0.00053	0.453
2	37	7	10	0.00013	0.129
2	37	7	11	0.00013	0.129
2	37	7	12	0.00026	0.241
2	37	7	16	0.00026	0.241
2	38	2	13	0.00013	0.129
2	38	2	16	0.00013	0.129
2	38	7	3	0.00013	0.129
2	38	7	4	0.00013	0.129
2	38	12	3	0.00013	0.129
2	38	12	4	0.00026	0.241
2	38	12	7	0.00013	0.129
2	38	12	11	0.00013	0.129
2	38	12	13	0.00013	0.129
2	38	12	15	0.00013	0.129
2	38	12	16	0.00013	0.129
2	38	16	4	0.00013	0.129
2	38	16	11	0.00013	0.129
2	38	17	7	0.00013	0.129
2	38	17	15	0.00013	0.129
2	39	2	1	0.00026	0.241
2	39	2	3	0.00013	0.129
2	39	2	11	0.00013	0.129
2	39	2	16	0.00013	0.129
2	39	5	3	0.00013	0.129
2	39	5	13	0.00013	0.129
2	39	7	1	0.00066	0.549
2	39	7	3	0.00053	0.453
2	39	7	7	0.00013	0.129
2	39	7	8	0.00013	0.129
2	39	7	11	0.00026	0.241
2	39	7	13	0.00053	0.453

2	39	7	16	0.00053	0.453
2	39	8	3	0.00013	0.129
2	39	8	16	0.00013	0.129
2	39	12	1	0.00026	0.241
2	39	12	3	0.00026	0.241
2	39	12	11	0.00013	0.129
2	39	12	16	0.00013	0.129
2	40	2	3	0.00013	0.129
2	40	2	15	0.00013	0.129
2	40	2	16	0.00013	0.129
2	40	3	3	0.00013	0.129
2	40	3	4	0.00026	0.241
2	40	3	11	0.00013	0.129
2	40	3	13	0.00013	0.129
2	40	4	15	0.00013	0.129
2	40	5	3	0.00026	0.241
2	40	5	13	0.00013	0.129
2	40	5	16	0.00013	0.129
2	40	7	4	0.00026	0.241
2	40	7	11	0.00013	0.129
2	41	2	3	0.00013	0.129
2	41	2	11	0.00013	0.129
2	41	4	4	0.00013	0.129
2	41	4	11	0.00013	0.129
2	41	4	15	0.00013	0.129
2	41	4	16	0.00013	0.129
2	41	5	3	0.00013	0.129
2	41	5	8	0.00026	0.241
2	41	5	13	0.00039	0.345
2	41	7	3	0.00026	0.241
2	41	7	4	0.00013	0.129
2	41	7	7	0.00013	0.129
2	41	7	13	0.00013	0.129
2	41	7	16	0.00053	0.453
2	41	12	7	0.00013	0.129
2	41	12	15	0.00013	0.129
2	41	17	3	0.00039	0.345
2	41	17	4	0.00026	0.241
2	41	17	7	0.00026	0.241
2	41	17	8	0.00026	0.241
2	41	17	11	0.00026	0.241
2	41	17	13	0.00053	0.453
2	41	17	15	0.00026	0.241
2	41	17	16	0.00053	0.453
2	44	1	4	0.00013	0.129
2	44	1	11	0.00013	0.129
2	44	2	1	0.00026	0.241
2	44	2	3	0.00013	0.129

2	44	2	4	0.00026	0.241
2	44	2	7	0.00013	0.129
2	44	2	11	0.00013	0.129
2	44	2	15	0.00013	0.129
2	44	2	16	0.00026	0.241
2	44	3	14	0.00013	0.129
2	44	3	16	0.00013	0.129
2	44	4	1	0.00013	0.129
2	44	4	3	0.00013	0.129
2	44	4	4	0.00013	0.129
2	44	4	11	0.00013	0.129
2	44	4	13	0.00026	0.241
2	44	4	15	0.00013	0.129
2	44	4	16	0.00013	0.129
2	44	5	1	0.00026	0.241
2	44	5	3	0.00131	1.000
2	44	5	4	0.00039	0.345
2	44	5	7	0.00013	0.129
2	44	5	8	0.00013	0.129
2	44	5	11	0.00039	0.345
2	44	5	12	0.00013	0.129
2	44	5	13	0.00039	0.345
2	44	5	14	0.00013	0.129
2	44	5	16	0.00118	0.914
2	44	6	7	0.00026	0.241
2	44	6	16	0.00013	0.129
2	44	7	3	0.00039	0.345
2	44	7	4	0.00039	0.345
2	44	7	7	0.00013	0.129
2	44	7	8	0.00013	0.129
2	44	7	11	0.00039	0.345
2	44	7	12	0.00013	0.129
2	44	7	13	0.00026	0.241
2	44	7	16	0.00079	0.643
2	44	8	1	0.00013	0.129
2	44	8	16	0.00013	0.129
2	44	12	4	0.00013	0.129
2	44	12	11	0.00013	0.129
2	44	14	4	0.00026	0.241
2	44	14	11	0.00026	0.241
2	44	16	3	0.00013	0.129
2	44	16	4	0.00013	0.129
2	44	16	7	0.00013	0.129
2	44	16	11	0.00026	0.241
2	45	6	3	0.00013	0.129
2	45	6	4	0.00013	0.129
2	45	6	11	0.00013	0.129
2	45	7	3	0.00013	0.129

2	45	7	4	0.00013	0.129
2	45	7	11	0.00013	0.129
2	47	6	11	0.00013	0.129
2	47	6	15	0.00013	0.129
2	47	15	11	0.00013	0.129
2	47	15	15	0.00013	0.129
2	49	1	4	0.00013	0.129
2	49	1	11	0.00026	0.241
2	49	2	4	0.00039	0.345
2	49	2	7	0.00039	0.345
2	49	2	11	0.00026	0.241
2	49	2	16	0.00013	0.129
2	49	3	4	0.00013	0.129
2	49	3	11	0.00013	0.129
2	49	4	1	0.00013	0.129
2	49	4	3	0.00013	0.129
2	49	4	11	0.00026	0.241
2	49	4	13	0.00026	0.241
2	49	4	16	0.00026	0.241
2	49	5	3	0.00066	0.549
2	49	5	4	0.00053	0.453
2	49	5	11	0.00013	0.129
2	49	6	3	0.00026	0.241
2	49	6	4	0.00039	0.345
2	49	7	1	0.00013	0.129
2	49	7	3	0.00171	1.26
2	49	7	4	0.00394	2.575
2	49	7	7	0.00053	0.453
2	49	7	10	0.00026	0.241
2	49	7	11	0.0021	1.505
2	49	7	13	0.00053	0.453
2	49	7	14	0.00013	0.129
2	49	7	16	0.00171	1.26
2	49	12	4	0.00026	0.241
2	49	12	11	0.00026	0.241
2	49	16	4	0.00013	0.129
2	49	16	11	0.00013	0.129
2	49	16	14	0.00013	0.129
2	49	16	16	0.00013	0.129
2	49	17	4	0.00013	0.129
2	49	17	7	0.00013	0.129
2	50	5	3	0.00013	0.129
2	50	5	13	0.00013	0.129
2	50	6	3	0.00013	0.129
2	50	6	4	0.00039	0.345
2	50	6	7	0.00026	0.241
2	50	6	11	0.00013	0.129
2	50	6	16	0.00026	0.241

2	50	7	4	0.00026	0.241
2	50	7	7	0.00026	0.241
2	50	7	11	0.00013	0.129
2	50	7	16	0.00026	0.241
2	50	15	3	0.00013	0.129
2	50	15	4	0.00013	0.129
2	51	1	1	0.00013	0.129
2	51	1	4	0.00039	0.345
2	51	1	11	0.00066	0.549
2	51	1	16	0.00013	0.129
2	51	2	1	0.00079	0.643
2	51	2	3	0.00066	0.549
2	51	2	4	0.00158	1.177
2	51	2	7	0.00079	0.643
2	51	2	8	0.00013	0.129
2	51	2	10	0.00013	0.129
2	51	2	11	0.0021	1.505
2	51	2	13	0.00013	0.129
2	51	2	16	0.00118	0.914
2	51	3	4	0.00013	0.129
2	51	3	16	0.00013	0.129
2	51	4	4	0.00039	0.345
2	51	4	11	0.00039	0.345
2	51	4	12	0.00013	0.129
2	51	4	13	0.00013	0.129
2	51	4	16	0.00053	0.453
2	51	5	1	0.00026	0.241
2	51	5	3	0.00079	0.643
2	51	5	4	0.00013	0.129
2	51	5	7	0.00013	0.129
2	51	5	11	0.00026	0.241
2	51	6	3	0.00013	0.129
2	51	6	4	0.00013	0.129
2	51	6	11	0.00013	0.129
2	51	6	15	0.00013	0.129
2	51	7	1	0.00053	0.453
2	51	7	3	0.00039	0.345
2	51	7	4	0.00092	0.735
2	51	7	7	0.00039	0.345
2	51	7	8	0.00013	0.129
2	51	7	11	0.00158	1.177
2	51	7	12	0.00013	0.129
2	51	7	14	0.00013	0.129
2	51	7	16	0.00145	1.093
2	51	8	1	0.00066	0.549
2	51	8	3	0.00013	0.129
2	51	8	4	0.00026	0.241
2	51	8	7	0.00026	0.241
2	51	8	11	0.00066	0.549
2	51	8	13	0.00013	0.129
2	51	8	16	0.00026	0.241
2	51	12	1	0.00013	0.129
2	51	12	3	0.00013	0.129
2	51	12	4	0.00013	0.129
2	51	12	11	0.00013	0.129
2	51	14	1	0.00013	0.129
2	51	14	3	0.00026	0.241
2	51	14	4	0.00026	0.241
2	51	14	11	0.00066	0.549
2	51	14	13	0.00013	0.129
2	51	14	16	0.00039	0.345
2	51	15	1	0.00053	0.453
2	51	15	3	0.00053	0.453
2	51	15	4	0.00026	0.241
2	51	15	11	0.00039	0.345
2	51	15	15	0.00013	0.129
2	51	15	16	0.00026	0.241
2	51	16	4	0.00066	0.549
2	51	16	10	0.00013	0.129
2	51	16	11	0.00053	0.453
2	51	16	14	0.00013	0.129
2	51	16	16	0.00013	0.129
2	51	17	3	0.00013	0.129
2	51	17	11	0.00013	0.129
2	52	4	11	0.00013	0.129
2	52	4	15	0.00013	0.129
2	52	7	4	0.00026	0.241
2	52	7	12	0.00013	0.129
2	52	7	15	0.00026	0.241
2	52	7	16	0.00013	0.129
2	52	8	1	0.00013	0.129
2	52	8	14	0.00013	0.129
2	52	12	1	0.00013	0.129
2	52	12	4	0.00026	0.241
2	52	12	11	0.00013	0.129
2	52	12	12	0.00013	0.129
2	52	12	14	0.00013	0.129
2	52	12	15	0.00039	0.345
2	52	12	16	0.00013	0.129
2	53	2	1	0.00013	0.129
2	53	2	4	0.00013	0.129
2	53	2	13	0.00013	0.129
2	53	2	15	0.00013	0.129
2	53	4	1	0.00026	0.241
2	53	4	4	0.00013	0.129
2	53	4	13	0.00026	0.241
2	53	4	15	0.00013	0.129
2	53	7	1	0.00013	0.129
2	53	7	13	0.00013	0.129
2	55	1	3	0.00013	0.129
2	55	1	11	0.00039	0.345
2	55	1	16	0.00013	0.129
2	55	3	1	0.00026	0.241
2	55	3	3	0.00026	0.241
2	55	3	4	0.00013	0.129
2	55	3	8	0.00013	0.129
2	55	3	11	0.00039	0.345
2	55	3	14	0.00053	0.453
2	55	3	16	0.00079	0.643
2	55	4	1	0.00013	0.129
2	55	4	8	0.00013	0.129
2	55	4	11	0.00013	0.129
2	55	4	14	0.00013	0.129
2	55	5	14	0.00013	0.129
2	55	5	16	0.00013	0.129
2	55	7	1	0.00013	0.129
2	55	7	3	0.00013	0.129
2	55	7	11	0.00039	0.345
2	55	7	14	0.00026	0.241
2	55	7	15	0.00013	0.129
2	55	7	16	0.00053	0.453
2	55	12	14	0.00013	0.129
2	55	12	16	0.00013	0.129
2	55	15	14	0.00013	0.129
2	55	15	15	0.00013	0.129
2	55	16	4	0.00013	0.129
2	55	16	16	0.00013	0.129
2	56	1	11	0.00013	0.129
2	56	7	11	0.00013	0.129
2	57	2	7	0.00013	0.129
2	57	2	16	0.00013	0.129
2	57	4	7	0.00013	0.129
2	57	4	11	0.00013	0.129
2	57	4	14	0.00013	0.129
2	57	4	16	0.00013	0.129
2	57	6	1	0.00013	0.129
2	57	6	7	0.00039	0.345
2	57	6	11	0.00013	0.129
2	57	6	14	0.00013	0.129
2	57	6	16	0.00013	0.129
2	57	7	7	0.00013	0.129
2	57	7	16	0.00013	0.129
2	57	8	1	0.00013	0.129
2	57	8	7	0.00013	0.129
2	57	16	7	0.00013	0.129
2	58	1	3	0.00013	0.129
2	58	1	11	0.00026	0.241
2	58	1	12	0.00013	0.129
2	58	1	16	0.00026	0.241
2	58	2	3	0.00013	0.129
2	58	2	4	0.00039	0.345
2	58	2	11	0.00026	0.241
2	58	2	16	0.00079	0.643
2	58	3	1	0.00013	0.129
2	58	3	3	0.00026	0.241
2	58	3	4	0.00013	0.129
2	58	3	7	0.00013	0.129
2	58	3	8	0.00013	0.129
2	58	3	10	0.00013	0.129
2	58	3	11	0.00053	0.453
2	58	3	16	0.00026	0.241
2	58	4	1	0.00026	0.241
2	58	4	4	0.00013	0.129
2	58	4	7	0.00013	0.129
2	58	4	8	0.00013	0.129
2	58	4	11	0.00039	0.345
2	58	4	12	0.00013	0.129
2	58	4	13	0.00026	0.241
2	58	4	16	0.00131	1.000
2	58	5	3	0.00145	1.093
2	58	5	4	0.00026	0.241
2	58	5	8	0.00013	0.129
2	58	5	10	0.00026	0.241
2	58	5	11	0.00013	0.129
2	58	5	16	0.00171	1.26
2	58	6	3	0.00026	0.241
2	58	6	4	0.00026	0.241
2	58	6	7	0.00066	0.549
2	58	6	10	0.00013	0.129
2	58	6	11	0.00013	0.129
2	58	6	12	0.00013	0.129
2	58	6	16	0.00079	0.643
2	58	7	1	0.00105	0.825
2	58	7	3	0.00394	2.575
2	58	7	4	0.00315	2.129
2	58	7	7	0.00092	0.735
2	58	7	8	0.00026	0.241
2	58	7	10	0.00079	0.643
2	58	7	11	0.00223	1.584
2	58	7	12	0.00053	0.453
2	58	7	13	0.00053	0.453
2	58	7	15	0.00026	0.241
2	58	7	16	0.01077	5.951
2	58	8	1	0.00013	0.129
2	58	8	16	0.00013	0.129
2	58	12	3	0.00013	0.129
2	58	12	4	0.00026	0.241
2	58	12	15	0.00013	0.129
2	58	12	16	0.00026	0.241
2	58	15	1	0.00013	0.129
2	58	15	4	0.00026	0.241
2	58	15	16	0.00053	0.453
2	58	16	16	0.00039	0.345
2	58	17	3	0.00013	0.129
2	58	17	13	0.00013	0.129
2	58	17	16	0.00039	0.345
2	73	5	3	0.00013	0.129
2	73	5	4	0.00013	0.129
2	73	7	4	0.00039	0.345
2	73	7	16	0.00026	0.241
2	73	15	3	0.00013	0.129
2	73	15	4	0.00053	0.453
2	73	15	16	0.00026	0.241
2	78	2	4	0.00013	0.129
2	78	2	10	0.00013	0.129
2	78	12	4	0.00013	0.129
2	78	12	10	0.00013	0.129
2	78	16	4	0.00026	0.241
2	78	16	10	0.00026	0.241
3	7	3	1	0.00013	0.129
3	7	3	13	0.00013	0.129
3	7	4	4	0.00013	0.129
3	7	4	11	0.00013	0.129
3	7	5	3	0.00053	0.453
3	7	5	4	0.00026	0.241
3	7	5	11	0.00013	0.129
3	7	5	15	0.00013	0.129
3	7	6	4	0.00013	0.129
3	7	6	7	0.00013	0.129
3	7	7	1	0.00039	0.345
3	7	7	3	0.00039	0.345
3	7	7	7	0.00026	0.241
3	7	7	9	0.00013	0.129
3	7	7	11	0.00039	0.345
3	7	7	13	0.00013	0.129
3	7	7	15	0.00079	0.643
3	7	7	16	0.00026	0.241
3	7	8	1	0.00013	0.129
3	7	8	3	0.00013	0.129
3	7	8	11	0.00013	0.129
3	7	8	15	0.00013	0.129
3	7	15	3	0.00026	0.241
3	7	15	4	0.00053	0.453
3	7	15	7	0.00026	0.241
3	7	15	11	0.00013	0.129
3	7	15	16	0.00013	0.129
3	7	16	1	0.00013	0.129
3	7	16	7	0.00013	0.129
3	7	16	9	0.00013	0.129
3	7	16	15	0.00013	0.129
3	8	4	7	0.00013	0.129
3	8	7	7	0.00013	0.129
3	13	6	1	0.00013	0.129
3	13	6	11	0.00013	0.129
3	13	6	13	0.00013	0.129
3	13	6	15	0.00013	0.129
3	13	8	1	0.00013	0.129
3	13	8	15	0.00013	0.129
3	13	12	11	0.00013	0.129
3	13	12	13	0.00013	0.129
3	14	4	1	0.00013	0.129
3	14	4	4	0.00013	0.129
3	14	5	1	0.00013	0.129
3	14	5	15	0.00013	0.129
3	14	6	1	0.00013	0.129
3	14	6	15	0.00013	0.129
3	14	7	1	0.00026	0.241
3	14	7	3	0.00013	0.129
3	14	7	4	0.00013	0.129
3	14	7	11	0.00013	0.129
3	14	7	15	0.00013	0.129
3	14	8	1	0.00053	0.453
3	14	8	3	0.00013	0.129
3	14	8	4	0.00026	0.241
3	14	8	11	0.00013	0.129
3	14	8	15	0.00026	0.241
3	14	12	1	0.00013	0.129
3	14	12	15	0.00013	0.129
3	15	3	4	0.00013	0.129
3	15	3	11	0.00013	0.129
3	15	7	4	0.00013	0.129
3	15	7	11	0.00013	0.129
3	15	7	15	0.00013	0.129
3	15	7	16	0.00013	0.129
3	18	3	4	0.00013	0.129
3	18	4	1	0.00026	0.241
3	18	4	4	0.00013	0.129
3	18	4	11	0.00013	0.129

3	18	4	16	0.00026	0.241
3	18	5	1	0.00013	0.129
3	18	5	3	0.00131	1.000
3	18	5	4	0.00039	0.345
3	18	5	11	0.00092	0.735
3	18	5	13	0.00013	0.129
3	18	5	15	0.00013	0.129
3	18	6	3	0.00013	0.129
3	18	6	11	0.00013	0.129
3	18	7	1	0.00039	0.345
3	18	7	3	0.00079	0.643
3	18	7	4	0.00053	0.453
3	18	7	11	0.00092	0.735
3	18	7	13	0.00039	0.345
3	18	7	15	0.00026	0.241
3	18	7	16	0.00039	0.345
3	18	8	1	0.00013	0.129
3	18	8	4	0.00013	0.129
3	18	12	3	0.00013	0.129
3	18	12	11	0.00053	0.453
3	18	12	13	0.00013	0.129
3	18	12	16	0.00013	0.129
3	18	15	3	0.00053	0.453
3	18	15	4	0.00026	0.241
3	18	15	11	0.00026	0.241
3	18	16	1	0.00013	0.129
3	18	16	15	0.00013	0.129
3	27	6	3	0.00013	0.129
3	27	6	11	0.00013	0.129
3	27	12	3	0.00013	0.129
3	27	12	11	0.00013	0.129
3	27	15	3	0.00026	0.241
3	27	15	11	0.00026	0.241
3	35	3	1	0.00026	0.241
3	35	3	4	0.00013	0.129
3	35	3	8	0.00013	0.129
3	35	3	14	0.00013	0.129
3	35	3	16	0.00013	0.129
3	35	4	1	0.00105	0.825
3	35	4	3	0.00026	0.241
3	35	4	4	0.00079	0.643
3	35	4	7	0.00026	0.241
3	35	4	8	0.00013	0.129
3	35	4	10	0.00013	0.129
3	35	4	11	0.00079	0.643
3	35	4	13	0.00026	0.241
3	35	4	14	0.00039	0.345
3	35	4	15	0.00013	0.129

3	35	4	16	0.00066	0.549
3	35	5	1	0.00013	0.129
3	35	5	3	0.00013	0.129
3	35	5	4	0.00013	0.129
3	35	5	7	0.00013	0.129
3	35	5	11	0.00013	0.129
3	35	5	13	0.00013	0.129
3	35	6	3	0.00013	0.129
3	35	6	4	0.00013	0.129
3	35	6	10	0.00013	0.129
3	35	6	11	0.00013	0.129
3	35	7	1	0.00026	0.241
3	35	7	4	0.00039	0.345
3	35	7	7	0.00013	0.129
3	35	7	10	0.00026	0.241
3	35	7	11	0.00013	0.129
3	35	7	13	0.00013	0.129
3	35	7	14	0.00013	0.129
3	35	7	16	0.00039	0.345
3	35	8	1	0.00013	0.129
3	35	8	4	0.00013	0.129
3	35	12	1	0.00013	0.129
3	35	12	3	0.00013	0.129
3	35	12	11	0.00013	0.129
3	35	12	14	0.00013	0.129
3	35	12	15	0.00013	0.129
3	35	15	3	0.00013	0.129
3	35	15	4	0.00013	0.129
3	35	15	7	0.00013	0.129
3	35	15	11	0.00026	0.241
3	35	15	16	0.00013	0.129
3	35	16	11	0.00013	0.129
3	37	5	3	0.00013	0.129
3	37	5	11	0.00013	0.129
3	37	6	3	0.00013	0.129
3	37	6	11	0.00013	0.129
3	38	3	7	0.00013	0.129
3	38	3	8	0.00013	0.129
3	38	4	1	0.00013	0.129
3	38	4	11	0.00013	0.129
3	38	5	1	0.00013	0.129
3	38	5	15	0.00013	0.129
3	38	6	11	0.00013	0.129
3	38	6	13	0.00013	0.129
3	38	12	1	0.00026	0.241
3	38	12	7	0.00013	0.129
3	38	12	8	0.00013	0.129
3	38	12	11	0.00026	0.241

3	38	12	13	0.00013	0.129
3	38	12	15	0.00013	0.129
3	39	7	1	0.00013	0.129
3	39	7	11	0.00013	0.129
3	39	15	1	0.00013	0.129
3	39	15	11	0.00013	0.129
3	40	3	4	0.00026	0.241
3	40	3	11	0.00013	0.129
3	40	7	4	0.00026	0.241
3	40	7	11	0.00013	0.129
3	41	6	4	0.00013	0.129
3	41	6	10	0.00013	0.129
3	41	7	4	0.00013	0.129
3	41	7	10	0.00013	0.129
3	44	1	4	0.00013	0.129
3	44	1	11	0.00013	0.129
3	44	3	4	0.00013	0.129
3	44	3	14	0.00013	0.129
3	44	4	3	0.00013	0.129
3	44	4	13	0.00013	0.129
3	44	5	3	0.00013	0.129
3	44	5	11	0.00013	0.129
3	44	5	13	0.00013	0.129
3	44	7	7	0.00013	0.129
3	44	7	9	0.00013	0.129
3	44	12	11	0.00013	0.129
3	44	16	4	0.00026	0.241
3	44	16	7	0.00013	0.129
3	44	16	9	0.00013	0.129
3	44	16	11	0.00013	0.129
3	44	16	14	0.00013	0.129
3	45	6	4	0.00013	0.129
3	45	6	7	0.00013	0.129
3	45	15	4	0.00013	0.129
3	45	15	7	0.00013	0.129
3	47	6	4	0.00013	0.129
3	47	6	11	0.00013	0.129
3	47	6	13	0.00013	0.129
3	47	6	15	0.00013	0.129
3	47	7	4	0.00013	0.129
3	47	7	13	0.00013	0.129
3	47	15	11	0.00013	0.129
3	47	15	15	0.00013	0.129
3	49	2	4	0.00013	0.129
3	49	2	16	0.00013	0.129
3	49	4	4	0.00013	0.129
3	49	4	11	0.00013	0.129
3	49	4	13	0.00013	0.129

3	49	4	14	0.00013	0.129
3	49	5	3	0.00053	0.453
3	49	5	4	0.00013	0.129
3	49	5	11	0.00026	0.241
3	49	5	13	0.00013	0.129
3	49	6	4	0.00013	0.129
3	49	6	13	0.00013	0.129
3	49	7	3	0.00053	0.453
3	49	7	4	0.00066	0.549
3	49	7	7	0.00013	0.129
3	49	7	11	0.00092	0.735
3	49	7	13	0.00066	0.549
3	49	7	14	0.00013	0.129
3	49	7	15	0.00013	0.129
3	49	7	16	0.00053	0.453
3	49	12	11	0.00026	0.241
3	49	12	13	0.00013	0.129
3	49	12	16	0.00013	0.129
3	51	1	4	0.00013	0.129
3	51	1	11	0.00013	0.129
3	51	2	4	0.00013	0.129
3	51	2	16	0.00013	0.129
3	51	4	4	0.00013	0.129
3	51	4	7	0.00013	0.129
3	51	4	11	0.00013	0.129
3	51	5	3	0.00013	0.129
3	51	5	4	0.00013	0.129
3	51	5	7	0.00013	0.129
3	51	5	11	0.00013	0.129
3	51	6	11	0.00013	0.129
3	51	6	15	0.00013	0.129
3	51	7	1	0.00013	0.129
3	51	7	4	0.00013	0.129
3	51	7	11	0.00013	0.129
3	51	7	16	0.00013	0.129
3	51	15	1	0.00013	0.129
3	51	15	3	0.00013	0.129
3	51	15	11	0.00039	0.345
3	51	15	15	0.00013	0.129
3	51	16	4	0.00013	0.129
3	51	16	11	0.00026	0.241
3	52	4	3	0.00013	0.129
3	52	4	14	0.00013	0.129
3	52	4	15	0.00013	0.129
3	52	12	3	0.00013	0.129
3	52	12	14	0.00013	0.129
3	52	12	15	0.00013	0.129
3	55	3	1	0.00026	0.241

3	55	3	4	0.00026	0.241
3	55	3	7	0.00013	0.129
3	55	3	8	0.00026	0.241
3	55	3	13	0.00013	0.129
3	55	3	14	0.00026	0.241
3	55	4	1	0.00013	0.129
3	55	4	4	0.00013	0.129
3	55	4	8	0.00013	0.129
3	55	4	14	0.00013	0.129
3	55	7	1	0.00013	0.129
3	55	7	13	0.00013	0.129
3	55	12	7	0.00013	0.129
3	55	12	8	0.00013	0.129
3	55	16	4	0.00013	0.129
3	55	16	14	0.00013	0.129
3	58	3	1	0.00013	0.129
3	58	3	16	0.00013	0.129
3	58	4	1	0.00026	0.241
3	58	4	10	0.00013	0.129
3	58	4	16	0.00026	0.241
3	58	7	1	0.00013	0.129
3	58	7	4	0.00013	0.129
3	58	7	10	0.00013	0.129
3	58	7	11	0.00013	0.129
3	58	7	15	0.00013	0.129
3	58	7	16	0.00053	0.453
11	7	1	4	0.00013	0.129
11	7	1	11	0.00013	0.129
11	7	3	1	0.00013	0.129
11	7	3	13	0.00013	0.129
11	7	4	4	0.00013	0.129
11	7	4	7	0.00013	0.129
11	7	7	1	0.00013	0.129
11	7	7	3	0.00013	0.129
11	7	7	13	0.00026	0.241
11	7	15	3	0.00013	0.129
11	7	15	4	0.00026	0.241
11	7	15	7	0.00013	0.129
11	7	15	11	0.00013	0.129
11	7	15	13	0.00013	0.129
11	8	3	3	0.00013	0.129
11	8	3	4	0.00013	0.129
11	8	3	14	0.00026	0.241
11	8	4	3	0.00013	0.129
11	8	4	16	0.00013	0.129
11	8	7	3	0.00026	0.241
11	8	7	4	0.00013	0.129
11	8	7	14	0.00026	0.241

11	8	7	16	0.00013	0.129
11	13	2	11	0.00013	0.129
11	13	2	16	0.00013	0.129
11	13	5	3	0.00013	0.129
11	13	5	4	0.00013	0.129
11	13	5	7	0.00013	0.129
11	13	5	11	0.00013	0.129
11	13	6	1	0.00013	0.129
11	13	6	3	0.00013	0.129
11	13	6	7	0.00039	0.345
11	13	6	11	0.00013	0.129
11	13	6	16	0.00013	0.129
11	13	7	4	0.00013	0.129
11	13	7	11	0.00013	0.129
11	13	15	1	0.00013	0.129
11	13	15	7	0.00013	0.129
11	14	4	1	0.00013	0.129
11	14	4	16	0.00013	0.129
11	14	7	3	0.00013	0.129
11	14	7	16	0.00013	0.129
11	14	8	1	0.00026	0.241
11	14	8	3	0.00013	0.129
11	14	8	16	0.00039	0.345
11	14	12	1	0.00013	0.129
11	14	12	16	0.00013	0.129
11	15	2	10	0.00026	0.241
11	15	2	11	0.00013	0.129
11	15	2	14	0.00013	0.129
11	15	3	3	0.00013	0.129
11	15	3	16	0.00013	0.129
11	15	4	10	0.00026	0.241
11	15	4	11	0.00013	0.129
11	15	4	14	0.00013	0.129
11	15	5	3	0.00013	0.129
11	15	5	16	0.00013	0.129
11	15	7	3	0.00013	0.129
11	15	7	13	0.00013	0.129
11	15	15	3	0.00013	0.129
11	15	15	13	0.00013	0.129
11	18	1	3	0.00026	0.241
11	18	1	4	0.00013	0.129
11	18	1	11	0.00026	0.241
11	18	1	13	0.00013	0.129
11	18	1	16	0.00026	0.241
11	18	2	3	0.00013	0.129
11	18	2	11	0.00013	0.129
11	18	2	14	0.00013	0.129
11	18	2	16	0.00013	0.129

11	18	3	3	0.00013	0.129
11	18	3	14	0.00013	0.129
11	18	3	16	0.00026	0.241
11	18	4	1	0.00013	0.129
11	18	4	3	0.00053	0.453
11	18	4	11	0.00013	0.129
11	18	4	13	0.00013	0.129
11	18	4	14	0.00013	0.129
11	18	4	16	0.00026	0.241
11	18	5	1	0.00026	0.241
11	18	5	3	0.00184	1.343
11	18	5	4	0.00026	0.241
11	18	5	7	0.00013	0.129
11	18	5	11	0.00026	0.241
11	18	5	12	0.00013	0.129
11	18	5	13	0.00026	0.241
11	18	5	14	0.00026	0.241
11	18	5	16	0.00066	0.549
11	18	6	1	0.00013	0.129
11	18	6	3	0.00039	0.345
11	18	6	4	0.00013	0.129
11	18	6	7	0.00013	0.129
11	18	6	11	0.00013	0.129
11	18	6	16	0.00026	0.241
11	18	7	3	0.00026	0.241
11	18	7	4	0.00026	0.241
11	18	7	7	0.00013	0.129
11	18	7	11	0.00026	0.241
11	18	7	13	0.00013	0.129
11	18	7	14	0.00013	0.129
11	18	7	16	0.00039	0.345
11	18	12	4	0.00013	0.129
11	18	12	11	0.00013	0.129
11	18	14	4	0.00013	0.129
11	18	14	11	0.00013	0.129
11	18	15	4	0.00013	0.129
11	18	15	11	0.00013	0.129
11	27	1	3	0.00013	0.129
11	27	1	13	0.00013	0.129
11	27	2	1	0.00013	0.129
11	27	2	15	0.00013	0.129
11	27	4	1	0.00013	0.129
11	27	4	15	0.00013	0.129
11	27	5	3	0.00013	0.129
11	27	5	13	0.00013	0.129
11	27	7	4	0.00013	0.129
11	27	7	16	0.00013	0.129
11	35	2	1	0.00026	0.241

11	35	2	10	0.00026	0.241
11	35	2	11	0.00013	0.129
11	35	2	14	0.00013	0.129
11	35	2	15	0.00026	0.241
11	35	2	16	0.00013	0.129
11	35	3	4	0.00013	0.129
11	35	3	7	0.00013	0.129
11	35	3	10	0.00013	0.129
11	35	3	12	0.00013	0.129
11	35	3	14	0.00026	0.241
11	35	4	1	0.00158	1.177
11	35	4	3	0.00066	0.549
11	35	4	4	0.00105	0.825
11	35	4	7	0.00039	0.345
11	35	4	10	0.00039	0.345
11	35	4	11	0.00105	0.825
11	35	4	12	0.00039	0.345
11	35	4	13	0.00026	0.241
11	35	4	14	0.00118	0.914
11	35	4	15	0.00039	0.345
11	35	4	16	0.00105	0.825
11	35	5	1	0.00026	0.241
11	35	5	3	0.00053	0.453
11	35	5	4	0.00013	0.129
11	35	5	11	0.00013	0.129
11	35	5	13	0.00013	0.129
11	35	5	14	0.00026	0.241
11	35	5	16	0.00039	0.345
11	35	6	7	0.00013	0.129
11	35	6	10	0.00013	0.129
11	35	6	11	0.00026	0.241
11	35	6	14	0.00013	0.129
11	35	7	1	0.00013	0.129
11	35	7	3	0.00013	0.129
11	35	7	4	0.00039	0.345
11	35	7	7	0.00013	0.129
11	35	7	10	0.00013	0.129
11	35	7	12	0.00013	0.129
11	35	7	13	0.00013	0.129
11	35	7	14	0.00026	0.241
11	35	7	16	0.00039	0.345
11	35	8	1	0.00013	0.129
11	35	8	16	0.00013	0.129
11	35	12	1	0.00039	0.345
11	35	12	4	0.00026	0.241
11	35	12	11	0.00026	0.241
11	35	12	12	0.00013	0.129
11	35	12	14	0.00013	0.129

11	35	12	15	0.00013	0.129
11	35	14	11	0.00013	0.129
11	35	14	16	0.00013	0.129
11	35	15	1	0.00013	0.129
11	35	15	4	0.00013	0.129
11	35	15	7	0.00013	0.129
11	35	16	1	0.00013	0.129
11	35	16	7	0.00013	0.129
11	37	4	10	0.00013	0.129
11	37	4	11	0.00013	0.129
11	37	4	14	0.00013	0.129
11	37	5	1	0.00013	0.129
11	37	5	3	0.00026	0.241
11	37	5	4	0.00013	0.129
11	37	5	16	0.00013	0.129
11	37	6	1	0.00013	0.129
11	37	6	3	0.00026	0.241
11	37	6	4	0.00026	0.241
11	37	6	10	0.00053	0.453
11	37	6	11	0.00013	0.129
11	37	6	14	0.00013	0.129
11	37	6	15	0.00013	0.129
11	37	6	16	0.00026	0.241
11	37	7	10	0.00013	0.129
11	37	7	16	0.00013	0.129
11	37	12	10	0.00013	0.129
11	37	12	15	0.00013	0.129
11	37	15	4	0.00013	0.129
11	37	15	10	0.00013	0.129
11	38	4	1	0.00013	0.129
11	38	4	4	0.00013	0.129
11	38	4	11	0.00026	0.241
11	38	4	12	0.00013	0.129
11	38	4	14	0.00013	0.129
11	38	12	1	0.00013	0.129
11	38	12	4	0.00013	0.129
11	38	12	11	0.00026	0.241
11	38	12	12	0.00013	0.129
11	38	12	14	0.00013	0.129
11	39	7	3	0.00013	0.129
11	39	7	7	0.00013	0.129
11	39	7	13	0.00013	0.129
11	39	7	16	0.00013	0.129
11	39	8	3	0.00013	0.129
11	39	8	16	0.00013	0.129
11	40	1	11	0.00013	0.129
11	40	1	13	0.00013	0.129
11	40	2	11	0.00013	0.129

11	40	2	13	0.00013	0.129
11	40	2	15	0.00013	0.129
11	40	4	15	0.00013	0.129
11	44	2	1	0.00013	0.129
11	44	2	3	0.00013	0.129
11	44	2	14	0.00026	0.241
11	44	2	16	0.00026	0.241
11	44	3	4	0.00013	0.129
11	44	3	14	0.00026	0.241
11	44	3	16	0.00013	0.129
11	44	4	1	0.00026	0.241
11	44	4	4	0.00013	0.129
11	44	4	7	0.00013	0.129
11	44	4	11	0.00013	0.129
11	44	4	14	0.00013	0.129
11	44	5	1	0.00013	0.129
11	44	5	3	0.00026	0.241
11	44	5	4	0.00026	0.241
11	44	5	12	0.00026	0.241
11	44	5	14	0.00039	0.345
11	44	5	16	0.00026	0.241
11	44	7	4	0.00013	0.129
11	44	7	12	0.00013	0.129
11	44	7	14	0.00013	0.129
11	44	7	16	0.00013	0.129
11	44	12	4	0.00013	0.129
11	44	12	11	0.00013	0.129
11	44	14	4	0.00013	0.129
11	44	14	11	0.00013	0.129
11	44	16	1	0.00013	0.129
11	44	16	4	0.00013	0.129
11	44	16	7	0.00013	0.129
11	44	16	14	0.00013	0.129
11	45	3	4	0.00013	0.129
11	45	6	4	0.00013	0.129
11	49	4	4	0.00039	0.345
11	49	4	12	0.00013	0.129
11	49	4	14	0.00026	0.241
11	49	5	3	0.00013	0.129
11	49	5	4	0.00026	0.241
11	49	5	11	0.00026	0.241
11	49	5	12	0.00013	0.129
11	49	7	3	0.00013	0.129
11	49	7	4	0.00066	0.549
11	49	7	11	0.00039	0.345
11	49	7	12	0.00026	0.241
11	49	7	14	0.00026	0.241
11	49	7	16	0.00013	0.129

11	50	4	7	0.00013	0.129
11	50	4	11	0.00013	0.129
11	50	6	3	0.00013	0.129
11	50	6	4	0.00013	0.129
11	50	6	7	0.00026	0.241
11	50	6	11	0.00013	0.129
11	50	15	3	0.00013	0.129
11	50	15	4	0.00013	0.129
11	51	1	3	0.00013	0.129
11	51	1	11	0.00026	0.241
11	51	1	13	0.00013	0.129
11	51	1	16	0.00026	0.241
11	51	2	11	0.00013	0.129
11	51	2	13	0.00013	0.129
11	51	4	1	0.00013	0.129
11	51	4	11	0.00013	0.129
11	51	4	12	0.00013	0.129
11	51	4	16	0.00026	0.241
11	51	5	3	0.00013	0.129
11	51	5	16	0.00013	0.129
11	51	6	1	0.00013	0.129
11	51	6	3	0.00013	0.129
11	51	6	4	0.00013	0.129
11	51	6	7	0.00013	0.129
11	51	7	11	0.00026	0.241
11	51	7	12	0.00013	0.129
11	51	7	16	0.00039	0.345
11	51	14	11	0.00013	0.129
11	51	14	16	0.00013	0.129
11	51	15	1	0.00026	0.241
11	51	15	3	0.00013	0.129
11	51	15	4	0.00013	0.129
11	51	15	7	0.00013	0.129
11	52	3	14	0.00013	0.129
11	52	3	15	0.00013	0.129
11	52	4	1	0.00013	0.129
11	52	4	15	0.00013	0.129
11	52	6	10	0.00013	0.129
11	52	6	15	0.00013	0.129
11	52	7	4	0.00013	0.129
11	52	7	15	0.00013	0.129
11	52	8	1	0.00013	0.129
11	52	8	16	0.00013	0.129
11	52	12	1	0.00026	0.241
11	52	12	4	0.00013	0.129
11	52	12	10	0.00013	0.129
11	52	12	14	0.00013	0.129
11	52	12	15	0.00053	0.453

11	52	12	16	0.00013	0.129
11	55	3	1	0.00039	0.345
11	55	3	3	0.00026	0.241
11	55	3	4	0.00053	0.453
11	55	3	12	0.00013	0.129
11	55	3	13	0.00013	0.129
11	55	3	14	0.00105	0.825
11	55	3	15	0.00013	0.129
11	55	3	16	0.00039	0.345
11	55	4	4	0.00013	0.129
11	55	4	12	0.00013	0.129
11	55	4	14	0.00026	0.241
11	55	5	14	0.00013	0.129
11	55	5	16	0.00013	0.129
11	55	6	4	0.00013	0.129
11	55	7	1	0.00039	0.345
11	55	7	3	0.00026	0.241
11	55	7	4	0.00013	0.129
11	55	7	13	0.00013	0.129
11	55	7	14	0.00039	0.345
11	55	7	16	0.00026	0.241
11	55	12	14	0.00013	0.129
11	55	12	15	0.00013	0.129
11	55	16	4	0.00013	0.129
11	55	16	14	0.00013	0.129
11	58	2	14	0.00013	0.129
11	58	2	16	0.00013	0.129
11	58	3	1	0.00026	0.241
11	58	3	3	0.00013	0.129
11	58	3	7	0.00013	0.129
11	58	3	10	0.00013	0.129
11	58	3	16	0.00013	0.129
11	58	4	1	0.00013	0.129
11	58	4	12	0.00013	0.129
11	58	4	13	0.00013	0.129
11	58	4	16	0.00039	0.345
11	58	6	10	0.00013	0.129
11	58	6	16	0.00013	0.129
11	58	7	1	0.00039	0.345
11	58	7	3	0.00013	0.129
11	58	7	4	0.00013	0.129
11	58	7	7	0.00013	0.129
11	58	7	10	0.00026	0.241
11	58	7	12	0.00013	0.129
11	58	7	13	0.00013	0.129
11	58	7	14	0.00013	0.129
11	58	7	15	0.00013	0.129
11	58	7	16	0.00092	0.735

11	58	12	4	0.00013	0.129
11	58	12	15	0.00013	0.129
11	73	6	4	0.00013	0.129
11	73	6	10	0.00013	0.129
11	73	15	4	0.00013	0.129
11	73	15	10	0.00013	0.129
23	8	7	11	0.00013	0.129
23	8	7	15	0.00013	0.129
23	13	6	4	0.00013	0.129
23	13	6	7	0.00013	0.129
23	13	7	4	0.00013	0.129
23	13	7	7	0.00013	0.129
23	14	2	1	0.00013	0.129
23	14	2	16	0.00013	0.129
23	14	3	1	0.00026	0.241
23	14	3	14	0.00013	0.129
23	14	3	16	0.00013	0.129
23	14	4	1	0.00013	0.129
23	14	4	4	0.00013	0.129
23	14	8	1	0.00053	0.453
23	14	8	3	0.00013	0.129
23	14	8	4	0.00013	0.129
23	14	8	13	0.00013	0.129
23	14	8	14	0.00013	0.129
23	14	8	16	0.00026	0.241
23	14	12	3	0.00013	0.129
23	14	12	13	0.00013	0.129
23	18	2	3	0.00013	0.129
23	18	2	4	0.00013	0.129
23	18	2	16	0.00026	0.241
23	18	5	3	0.00053	0.453
23	18	5	4	0.00013	0.129
23	18	5	16	0.00039	0.345
23	18	7	3	0.00039	0.345
23	18	7	4	0.00026	0.241
23	18	7	11	0.00013	0.129
23	18	7	15	0.00013	0.129
23	18	7	16	0.00039	0.345
23	27	2	1	0.00013	0.129
23	27	2	3	0.00013	0.129
23	27	2	4	0.00013	0.129
23	27	2	16	0.00039	0.345
23	27	5	3	0.00013	0.129
23	27	5	16	0.00013	0.129
23	27	7	4	0.00013	0.129
23	27	7	16	0.00013	0.129
23	27	8	1	0.00013	0.129
23	27	8	16	0.00013	0.129

23	35	3	11	0.00013	0.129
23	35	3	14	0.00013	0.129
23	35	4	1	0.00013	0.129
23	35	4	11	0.00039	0.345
23	35	4	14	0.00013	0.129
23	35	6	11	0.00013	0.129
23	38	8	3	0.00013	0.129
23	38	8	13	0.00013	0.129
23	38	12	3	0.00013	0.129
23	38	12	13	0.00013	0.129
23	41	7	3	0.00013	0.129
23	41	7	16	0.00013	0.129
23	44	2	4	0.00013	0.129
23	44	2	11	0.00013	0.129
23	44	4	1	0.00026	0.241
23	44	4	3	0.00013	0.129
23	44	4	4	0.00026	0.241
23	44	4	8	0.00013	0.129
23	44	4	11	0.00039	0.345
23	44	6	11	0.00013	0.129
23	44	7	3	0.00013	0.129
23	44	7	8	0.00013	0.129
23	44	8	1	0.00013	0.129
23	44	8	4	0.00013	0.129
23	49	2	7	0.00013	0.129
23	49	2	11	0.00013	0.129
23	49	3	4	0.00013	0.129
23	49	3	11	0.00013	0.129
23	49	5	3	0.00013	0.129
23	49	5	4	0.00013	0.129
23	49	6	4	0.00013	0.129
23	49	6	7	0.00013	0.129
23	49	7	3	0.00013	0.129
23	49	7	4	0.00039	0.345
23	49	7	7	0.00026	0.241
23	49	7	11	0.00026	0.241
23	51	2	4	0.00013	0.129
23	51	2	7	0.00013	0.129
23	51	2	11	0.00026	0.241
23	51	4	4	0.00013	0.129
23	51	4	11	0.00013	0.129
23	51	7	7	0.00013	0.129
23	51	7	11	0.00013	0.129
23	55	3	1	0.00026	0.241
23	55	3	11	0.00013	0.129
23	55	3	14	0.00026	0.241
23	55	3	16	0.00013	0.129
23	55	4	11	0.00013	0.129

23	55	4	14	0.00013	0.129
23	55	8	1	0.00026	0.241
23	55	8	14	0.00013	0.129
23	55	8	16	0.00013	0.129
23	58	3	4	0.00013	0.129
23	58	3	11	0.00013	0.129
23	58	4	3	0.00013	0.129
23	58	4	8	0.00013	0.129
23	58	5	3	0.00026	0.241
23	58	5	16	0.00026	0.241
23	58	7	3	0.00053	0.453
23	58	7	4	0.00013	0.129
23	58	7	8	0.00013	0.129
23	58	7	11	0.00013	0.129
23	58	7	16	0.00039	0.345
24	7	2	1	0.00013	0.129
24	7	2	16	0.00013	0.129
24	7	4	4	0.00013	0.129
24	7	4	7	0.00013	0.129
24	7	5	1	0.00026	0.241
24	7	5	3	0.00039	0.345
24	7	5	4	0.00013	0.129
24	7	7	1	0.00026	0.241
24	7	7	3	0.00053	0.453
24	7	7	4	0.00026	0.241
24	7	7	11	0.00026	0.241
24	7	7	13	0.00013	0.129
24	7	7	14	0.00013	0.129
24	7	7	15	0.00026	0.241
24	7	7	16	0.00013	0.129
24	7	15	1	0.00013	0.129
24	7	15	3	0.00013	0.129
24	7	15	4	0.00013	0.129
24	7	15	7	0.00013	0.129
24	7	15	13	0.00013	0.129
24	7	15	14	0.00013	0.129
24	7	15	15	0.00013	0.129
24	7	15	16	0.00013	0.129
24	8	3	3	0.00013	0.129
24	8	3	16	0.00013	0.129
24	8	7	1	0.00013	0.129
24	8	7	3	0.00013	0.129
24	8	7	10	0.00013	0.129
24	8	7	16	0.00013	0.129
24	8	8	1	0.00013	0.129
24	8	8	10	0.00013	0.129
24	13	4	3	0.00013	0.129
24	13	4	4	0.00013	0.129

24	13	4	11	0.00039	0.345
24	13	4	15	0.00026	0.241
24	13	4	16	0.00013	0.129
24	13	5	3	0.00013	0.129
24	13	5	15	0.00013	0.129
24	13	6	4	0.00013	0.129
24	13	6	11	0.00026	0.241
24	13	6	16	0.00013	0.129
24	14	2	1	0.00026	0.241
24	14	2	11	0.00013	0.129
24	14	2	16	0.00013	0.129
24	14	4	1	0.00039	0.345
24	14	4	4	0.00026	0.241
24	14	4	7	0.00013	0.129
24	14	4	11	0.00013	0.129
24	14	4	16	0.00013	0.129
24	14	5	1	0.00026	0.241
24	14	5	3	0.00026	0.241
24	14	6	1	0.00013	0.129
24	14	6	13	0.00013	0.129
24	14	7	1	0.00026	0.241
24	14	7	10	0.00013	0.129
24	14	7	16	0.00013	0.129
24	14	8	1	0.00171	1.26
24	14	8	3	0.00026	0.241
24	14	8	4	0.00039	0.345
24	14	8	7	0.00026	0.241
24	14	8	10	0.00013	0.129
24	14	8	11	0.00066	0.549
24	14	8	13	0.00013	0.129
24	14	8	16	0.00039	0.345
24	14	12	1	0.00013	0.129
24	14	12	11	0.00013	0.129
24	14	14	1	0.00013	0.129
24	14	14	11	0.00013	0.129
24	14	15	4	0.00013	0.129
24	14	15	11	0.00013	0.129
24	15	1	3	0.00013	0.129
24	15	1	4	0.00013	0.129
24	15	2	10	0.00013	0.129
24	15	2	16	0.00013	0.129
24	15	3	1	0.00013	0.129
24	15	3	11	0.00026	0.241
24	15	3	13	0.00013	0.129
24	15	3	16	0.00013	0.129
24	15	5	4	0.00013	0.129
24	15	5	13	0.00013	0.129
24	15	7	1	0.00013	0.129

24	15	7	3	0.00026	0.241
24	15	7	4	0.00026	0.241
24	15	7	10	0.00013	0.129
24	15	7	11	0.00013	0.129
24	15	7	13	0.00039	0.345
24	15	7	15	0.00013	0.129
24	15	7	16	0.00039	0.345
24	15	12	11	0.00013	0.129
24	15	15	3	0.00013	0.129
24	15	15	13	0.00013	0.129
24	18	2	3	0.00026	0.241
24	18	2	10	0.00013	0.129
24	18	2	11	0.00013	0.129
24	18	2	16	0.00026	0.241
24	18	3	3	0.00026	0.241
24	18	3	4	0.00013	0.129
24	18	3	11	0.00013	0.129
24	18	3	13	0.00013	0.129
24	18	3	14	0.00039	0.345
24	18	3	16	0.00013	0.129
24	18	4	3	0.00053	0.453
24	18	4	4	0.00013	0.129
24	18	4	7	0.00013	0.129
24	18	4	8	0.00013	0.129
24	18	4	11	0.00039	0.345
24	18	4	13	0.00026	0.241
24	18	4	15	0.00013	0.129
24	18	4	16	0.00026	0.241
24	18	5	1	0.00053	0.453
24	18	5	3	0.00263	1.825
24	18	5	4	0.00066	0.549
24	18	5	8	0.00013	0.129
24	18	5	11	0.00053	0.453
24	18	5	12	0.00013	0.129
24	18	5	13	0.00039	0.345
24	18	5	14	0.00039	0.345
24	18	5	15	0.00026	0.241
24	18	5	16	0.00053	0.453
24	18	6	3	0.00013	0.129
24	18	6	4	0.00026	0.241
24	18	6	11	0.00013	0.129
24	18	7	1	0.00026	0.241
24	18	7	3	0.00079	0.643
24	18	7	4	0.00079	0.643
24	18	7	11	0.00131	1.000
24	18	7	12	0.00013	0.129
24	18	7	13	0.00039	0.345
24	18	7	14	0.00013	0.129

24	18	7	15	0.00013	0.129
24	18	7	16	0.00079	0.643
24	18	8	1	0.00039	0.345
24	18	8	3	0.00026	0.241
24	18	8	11	0.00013	0.129
24	18	12	1	0.00013	0.129
24	18	12	3	0.00026	0.241
24	18	12	4	0.00013	0.129
24	18	12	7	0.00013	0.129
24	18	12	10	0.00013	0.129
24	18	12	11	0.00145	1.093
24	18	12	13	0.00039	0.345
24	18	12	14	0.00013	0.129
24	18	12	15	0.00013	0.129
24	18	12	16	0.00039	0.345
24	18	16	3	0.00013	0.129
24	18	16	13	0.00013	0.129
24	27	1	1	0.00013	0.129
24	27	1	11	0.00013	0.129
24	27	2	1	0.00013	0.129
24	27	2	3	0.00013	0.129
24	27	2	4	0.00026	0.241
24	27	2	8	0.00013	0.129
24	27	2	10	0.00013	0.129
24	27	2	11	0.00013	0.129
24	27	2	16	0.00026	0.241
24	27	4	1	0.00013	0.129
24	27	4	11	0.00013	0.129
24	27	5	3	0.00013	0.129
24	27	5	4	0.00013	0.129
24	27	7	4	0.00013	0.129
24	27	7	8	0.00013	0.129
24	27	7	10	0.00013	0.129
24	27	8	1	0.00013	0.129
24	27	8	16	0.00013	0.129
24	27	15	11	0.00013	0.129
24	27	15	16	0.00013	0.129
24	35	1	1	0.00013	0.129
24	35	1	11	0.00013	0.129
24	35	2	10	0.00013	0.129
24	35	2	11	0.00026	0.241
24	35	2	16	0.00013	0.129
24	35	3	12	0.00013	0.129
24	35	3	14	0.00013	0.129
24	35	4	1	0.00053	0.453
24	35	4	3	0.00026	0.241
24	35	4	4	0.00079	0.643
24	35	4	7	0.00053	0.453

24	35	4	10	0.00013	0.129
24	35	4	11	0.00197	1.424
24	35	4	12	0.00026	0.241
24	35	4	13	0.00013	0.129
24	35	4	14	0.00013	0.129
24	35	4	15	0.00053	0.453
24	35	4	16	0.00105	0.825
24	35	5	3	0.00039	0.345
24	35	5	4	0.00026	0.241
24	35	5	7	0.00013	0.129
24	35	5	13	0.00013	0.129
24	35	5	14	0.00013	0.129
24	35	6	4	0.00013	0.129
24	35	6	11	0.00026	0.241
24	35	6	16	0.00013	0.129
24	35	7	1	0.00013	0.129
24	35	7	4	0.00013	0.129
24	35	7	10	0.00013	0.129
24	35	7	11	0.00039	0.345
24	35	7	15	0.00013	0.129
24	35	7	16	0.00066	0.549
24	35	8	1	0.00013	0.129
24	35	8	7	0.00013	0.129
24	35	8	11	0.00013	0.129
24	35	8	16	0.00013	0.129
24	35	12	1	0.00013	0.129
24	35	12	3	0.00013	0.129
24	35	12	4	0.00013	0.129
24	35	12	7	0.00013	0.129
24	35	12	11	0.00079	0.643
24	35	12	14	0.00013	0.129
24	35	12	15	0.00026	0.241
24	35	12	16	0.00026	0.241
24	35	15	4	0.00013	0.129
24	35	15	7	0.00013	0.129
24	35	15	10	0.00013	0.129
24	35	15	11	0.00026	0.241
24	35	15	16	0.00013	0.129
24	35	16	11	0.00013	0.129
24	35	16	12	0.00013	0.129
24	35	16	16	0.00026	0.241
24	37	2	3	0.00013	0.129
24	37	2	16	0.00013	0.129
24	37	6	3	0.00013	0.129
24	37	6	16	0.00013	0.129
24	38	4	11	0.00013	0.129
24	38	7	12	0.00013	0.129
24	38	7	13	0.00013	0.129

24	38	12	11	0.00013	0.129
24	38	12	12	0.00013	0.129
24	38	12	13	0.00013	0.129
24	39	4	11	0.00013	0.129
24	39	4	15	0.00013	0.129
24	39	7	3	0.00013	0.129
24	39	7	7	0.00013	0.129
24	39	7	11	0.00013	0.129
24	39	7	15	0.00013	0.129
24	39	7	16	0.00053	0.453
24	39	15	3	0.00013	0.129
24	39	15	16	0.00013	0.129
24	39	16	7	0.00013	0.129
24	39	16	16	0.00013	0.129
24	40	1	11	0.00013	0.129
24	40	1	13	0.00013	0.129
24	40	2	3	0.00039	0.345
24	40	2	11	0.00026	0.241
24	40	2	13	0.00013	0.129
24	40	2	15	0.00013	0.129
24	40	2	16	0.00039	0.345
24	40	5	3	0.00026	0.241
24	40	5	11	0.00013	0.129
24	40	5	16	0.00013	0.129
24	40	6	3	0.00013	0.129
24	40	6	16	0.00013	0.129
24	40	7	15	0.00013	0.129
24	40	7	16	0.00013	0.129
24	41	7	3	0.00013	0.129
24	41	7	4	0.00013	0.129
24	41	16	1	0.00013	0.129
24	41	16	3	0.00013	0.129
24	41	17	1	0.00013	0.129
24	41	17	3	0.00026	0.241
24	41	17	4	0.00013	0.129
24	44	2	1	0.00013	0.129
24	44	2	3	0.00013	0.129
24	44	2	4	0.00013	0.129
24	44	2	10	0.00013	0.129
24	44	2	16	0.00026	0.241
24	44	4	1	0.00013	0.129
24	44	4	3	0.00013	0.129
24	44	4	4	0.00013	0.129
24	44	4	8	0.00013	0.129
24	44	5	3	0.00026	0.241
24	44	5	4	0.00013	0.129
24	44	5	11	0.00013	0.129
24	44	5	12	0.00013	0.129

24	44	5	16	0.00026	0.241
24	44	7	3	0.00026	0.241
24	44	7	7	0.00013	0.129
24	44	7	8	0.00013	0.129
24	44	7	11	0.00013	0.129
24	44	7	12	0.00013	0.129
24	44	7	16	0.00039	0.345
24	44	8	1	0.00013	0.129
24	44	8	4	0.00013	0.129
24	44	12	10	0.00013	0.129
24	44	12	16	0.00013	0.129
24	44	15	1	0.00013	0.129
24	44	15	16	0.00013	0.129
24	44	16	7	0.00013	0.129
24	44	16	16	0.00013	0.129
24	45	3	11	0.00013	0.129
24	45	3	16	0.00013	0.129
24	45	4	11	0.00013	0.129
24	45	4	12	0.00013	0.129
24	45	6	1	0.00013	0.129
24	45	6	13	0.00013	0.129
24	45	7	11	0.00013	0.129
24	45	7	16	0.00013	0.129
24	45	8	1	0.00013	0.129
24	45	8	13	0.00013	0.129
24	45	16	11	0.00013	0.129
24	45	16	12	0.00013	0.129
24	49	1	4	0.00013	0.129
24	49	1	11	0.00013	0.129
24	49	2	4	0.00013	0.129
24	49	7	3	0.00026	0.241
24	49	7	4	0.00066	0.549
24	49	7	11	0.00079	0.643
24	49	7	12	0.00013	0.129
24	49	7	13	0.00039	0.345
24	49	7	14	0.00013	0.129
24	49	12	4	0.00013	0.129
24	49	12	11	0.00026	0.241
24	49	12	12	0.00013	0.129
24	49	12	13	0.00026	0.241
24	49	16	11	0.00013	0.129
24	49	16	14	0.00013	0.129
24	49	17	3	0.00013	0.129
24	49	17	4	0.00013	0.129
24	50	4	1	0.00013	0.129
24	50	4	4	0.00013	0.129
24	50	5	3	0.00013	0.129
24	50	5	4	0.00013	0.129

24	50	6	3	0.00013	0.129
24	50	6	4	0.00026	0.241
24	50	6	7	0.00013	0.129
24	50	6	11	0.00013	0.129
24	50	6	16	0.00013	0.129
24	50	7	4	0.00013	0.129
24	50	7	7	0.00013	0.129
24	50	7	11	0.00013	0.129
24	50	7	16	0.00013	0.129
24	50	8	1	0.00013	0.129
24	50	8	4	0.00013	0.129
24	51	1	4	0.00013	0.129
24	51	1	11	0.00026	0.241
24	51	1	13	0.00013	0.129
24	51	2	1	0.00013	0.129
24	51	2	11	0.00026	0.241
24	51	2	13	0.00013	0.129
24	51	4	4	0.00013	0.129
24	51	4	7	0.00013	0.129
24	51	5	3	0.00026	0.241
24	51	5	4	0.00013	0.129
24	51	5	7	0.00013	0.129
24	51	5	11	0.00013	0.129
24	51	5	13	0.00013	0.129
24	51	7	3	0.00013	0.129
24	51	7	4	0.00013	0.129
24	51	7	11	0.00026	0.241
24	51	7	14	0.00013	0.129
24	51	7	16	0.00013	0.129
24	51	8	1	0.00026	0.241
24	51	8	4	0.00013	0.129
24	51	8	11	0.00039	0.345
24	51	12	3	0.00013	0.129
24	51	12	11	0.00013	0.129
24	51	14	1	0.00013	0.129
24	51	14	11	0.00013	0.129
24	51	15	3	0.00013	0.129
24	51	15	4	0.00013	0.129
24	51	15	11	0.00013	0.129
24	51	15	16	0.00013	0.129
24	51	16	1	0.00013	0.129
24	51	16	3	0.00026	0.241
24	51	16	11	0.00013	0.129
24	51	16	13	0.00013	0.129
24	51	16	14	0.00013	0.129
24	51	17	1	0.00013	0.129
24	51	17	3	0.00013	0.129
24	52	4	1	0.00013	0.129

24	52	4	4	0.00013	0.129
24	52	4	11	0.00039	0.345
24	52	4	15	0.00026	0.241
24	52	12	1	0.00013	0.129
24	52	12	4	0.00013	0.129
24	52	12	11	0.00053	0.453
24	52	12	15	0.00039	0.345
24	53	4	11	0.00013	0.129
24	53	4	13	0.00013	0.129
24	53	12	11	0.00013	0.129
24	53	12	13	0.00013	0.129
24	55	3	1	0.00026	0.241
24	55	3	3	0.00039	0.345
24	55	3	4	0.00013	0.129
24	55	3	11	0.00026	0.241
24	55	3	12	0.00013	0.129
24	55	3	13	0.00026	0.241
24	55	3	14	0.00053	0.453
24	55	3	16	0.00066	0.549
24	55	4	12	0.00013	0.129
24	55	4	14	0.00013	0.129
24	55	5	3	0.00026	0.241
24	55	5	14	0.00026	0.241
24	55	7	1	0.00026	0.241
24	55	7	3	0.00013	0.129
24	55	7	4	0.00013	0.129
24	55	7	11	0.00026	0.241
24	55	7	13	0.00026	0.241
24	55	7	14	0.00026	0.241
24	55	7	15	0.00013	0.129
24	55	7	16	0.00066	0.549
24	55	15	14	0.00013	0.129
24	55	15	15	0.00013	0.129
24	56	1	3	0.00013	0.129
24	56	1	4	0.00013	0.129
24	56	7	3	0.00013	0.129
24	56	7	4	0.00013	0.129
24	57	6	7	0.00013	0.129
24	57	6	10	0.00013	0.129
24	57	16	7	0.00013	0.129
24	57	16	10	0.00013	0.129
24	58	2	8	0.00013	0.129
24	58	2	10	0.00026	0.241
24	58	2	15	0.00013	0.129
24	58	2	16	0.00026	0.241
24	58	3	1	0.00013	0.129
24	58	3	16	0.00013	0.129
24	58	4	1	0.00013	0.129

24	58	4	3	0.00026	0.241
24	58	4	4	0.00013	0.129
24	58	4	8	0.00026	0.241
24	58	4	10	0.00013	0.129
24	58	4	11	0.00026	0.241
24	58	4	16	0.00039	0.345
24	58	5	3	0.00066	0.549
24	58	5	4	0.00013	0.129
24	58	5	8	0.00013	0.129
24	58	5	10	0.00013	0.129
24	58	5	11	0.00013	0.129
24	58	5	15	0.00013	0.129
24	58	5	16	0.00053	0.453
24	58	6	7	0.00013	0.129
24	58	6	16	0.00013	0.129
24	58	7	1	0.00039	0.345
24	58	7	3	0.00079	0.643
24	58	7	4	0.00026	0.241
24	58	7	7	0.00013	0.129
24	58	7	8	0.00026	0.241
24	58	7	10	0.00053	0.453
24	58	7	11	0.00066	0.549
24	58	7	13	0.00013	0.129
24	58	7	15	0.00026	0.241
24	58	7	16	0.00223	1.584
24	58	8	1	0.00013	0.129
24	58	8	16	0.00013	0.129
24	58	16	16	0.00026	0.241
24	78	6	7	0.00013	0.129
24	78	6	10	0.00013	0.129
24	78	16	7	0.00013	0.129
24	78	16	10	0.00013	0.129
25	35	4	1	0.00013	0.129
25	35	4	13	0.00013	0.129
25	35	5	1	0.00013	0.129
25	35	5	13	0.00013	0.129
25	44	4	1	0.00013	0.129
25	44	4	13	0.00013	0.129
25	44	5	1	0.00013	0.129
25	44	5	13	0.00013	0.129
25	44	7	11	0.00013	0.129
25	44	7	16	0.00013	0.129
25	58	7	11	0.00013	0.129
25	58	7	16	0.00013	0.129
26	7	4	4	0.00013	0.129
26	7	4	11	0.00013	0.129
26	7	6	4	0.00013	0.129
26	7	6	7	0.00013	0.129

26	7	15	4	0.00026	0.241
26	7	15	7	0.00013	0.129
26	7	15	11	0.00013	0.129
26	14	2	3	0.00013	0.129
26	14	2	11	0.00013	0.129
26	14	3	3	0.00013	0.129
26	14	3	11	0.00013	0.129
26	14	8	1	0.00013	0.129
26	14	8	3	0.00026	0.241
26	14	8	4	0.00013	0.129
26	14	8	11	0.00026	0.241
26	14	12	1	0.00013	0.129
26	14	12	4	0.00013	0.129
26	15	1	3	0.00013	0.129
26	15	1	4	0.00013	0.129
26	15	7	3	0.00013	0.129
26	15	7	4	0.00013	0.129
26	18	1	11	0.00013	0.129
26	18	2	3	0.00013	0.129
26	18	2	16	0.00013	0.129
26	18	3	4	0.00013	0.129
26	18	3	14	0.00013	0.129
26	18	4	4	0.00013	0.129
26	18	4	11	0.00013	0.129
26	18	4	16	0.00026	0.241
26	18	5	3	0.00053	0.453
26	18	5	11	0.00026	0.241
26	18	5	16	0.00026	0.241
26	18	7	3	0.00013	0.129
26	18	7	4	0.00039	0.345
26	18	7	11	0.00013	0.129
26	18	7	14	0.00013	0.129
26	18	7	16	0.00013	0.129
26	18	12	3	0.00013	0.129
26	18	12	11	0.00026	0.241
26	18	12	16	0.00026	0.241
26	18	16	3	0.00013	0.129
26	18	16	11	0.00013	0.129
26	27	6	3	0.00013	0.129
26	27	6	11	0.00013	0.129
26	27	15	3	0.00013	0.129
26	27	15	11	0.00013	0.129
26	35	1	1	0.00013	0.129
26	35	1	11	0.00013	0.129
26	35	3	14	0.00013	0.129
26	35	3	16	0.00013	0.129
26	35	4	1	0.00013	0.129
26	35	4	4	0.00053	0.453

26	35	4	11	0.00053	0.453
26	35	4	14	0.00013	0.129
26	35	4	16	0.00053	0.453
26	35	6	3	0.00013	0.129
26	35	6	11	0.00013	0.129
26	35	7	11	0.00013	0.129
26	35	7	16	0.00013	0.129
26	35	12	4	0.00026	0.241
26	35	12	11	0.00013	0.129
26	35	12	14	0.00026	0.241
26	35	12	16	0.00039	0.345
26	35	15	3	0.00013	0.129
26	35	15	4	0.00053	0.453
26	35	15	11	0.00026	0.241
26	35	15	16	0.00039	0.345
26	38	4	4	0.00013	0.129
26	38	4	14	0.00013	0.129
26	38	5	3	0.00013	0.129
26	38	5	16	0.00013	0.129
26	38	7	3	0.00013	0.129
26	38	7	4	0.00013	0.129
26	38	8	1	0.00013	0.129
26	38	8	4	0.00013	0.129
26	38	12	1	0.00013	0.129
26	38	12	3	0.00026	0.241
26	38	12	4	0.00066	0.549
26	38	12	7	0.00013	0.129
26	38	12	11	0.00013	0.129
26	38	12	14	0.00013	0.129
26	38	12	15	0.00013	0.129
26	38	12	16	0.00026	0.241
26	38	15	4	0.00013	0.129
26	38	15	16	0.00013	0.129
26	38	16	4	0.00013	0.129
26	38	16	11	0.00013	0.129
26	38	17	7	0.00013	0.129
26	38	17	15	0.00013	0.129
26	41	12	7	0.00013	0.129
26	41	12	15	0.00013	0.129
26	41	17	7	0.00013	0.129
26	41	17	15	0.00013	0.129
26	44	5	3	0.00013	0.129
26	44	5	11	0.00013	0.129
26	44	16	3	0.00013	0.129
26	44	16	11	0.00013	0.129
26	45	6	4	0.00026	0.241
26	45	6	7	0.00026	0.241
26	45	15	4	0.00013	0.129

26	45	15	7	0.00013	0.129
26	49	4	11	0.00013	0.129
26	49	4	16	0.00013	0.129
26	49	7	4	0.00013	0.129
26	49	7	11	0.00013	0.129
26	49	7	16	0.00013	0.129
26	50	6	4	0.00013	0.129
26	50	6	7	0.00013	0.129
26	51	1	1	0.00013	0.129
26	51	1	11	0.00013	0.129
26	51	2	3	0.00026	0.241
26	51	2	11	0.00013	0.129
26	51	2	16	0.00013	0.129
26	51	4	1	0.00013	0.129
26	51	4	4	0.00013	0.129
26	51	4	11	0.00013	0.129
26	51	4	16	0.00013	0.129
26	51	5	3	0.00026	0.241
26	51	5	11	0.00013	0.129
26	51	5	16	0.00013	0.129
26	51	7	3	0.00013	0.129
26	51	7	11	0.00013	0.129
26	51	8	3	0.00013	0.129
26	51	8	11	0.00013	0.129
26	51	12	4	0.00013	0.129
26	51	12	11	0.00013	0.129
26	51	15	4	0.00013	0.129
26	51	15	16	0.00013	0.129
26	51	16	4	0.00013	0.129
26	51	16	11	0.00013	0.129
26	53	4	4	0.00013	0.129
26	53	4	16	0.00013	0.129
26	53	7	4	0.00013	0.129
26	53	7	16	0.00013	0.129
26	55	1	11	0.00013	0.129
26	55	3	3	0.00013	0.129
26	55	3	4	0.00013	0.129
26	55	3	11	0.00013	0.129
26	55	3	14	0.00026	0.241
26	55	3	16	0.00013	0.129
26	55	7	4	0.00013	0.129
26	55	7	14	0.00013	0.129
26	55	8	3	0.00013	0.129
26	55	8	11	0.00013	0.129
26	55	12	11	0.00013	0.129
26	55	12	14	0.00013	0.129
26	55	12	16	0.00013	0.129
26	56	1	3	0.00013	0.129

26	56	1	4	0.00013	0.129
26	56	7	3	0.00013	0.129
26	56	7	4	0.00013	0.129
26	58	7	3	0.00013	0.129
26	58	7	4	0.00013	0.129
26	58	7	11	0.00013	0.129
26	58	7	16	0.00013	0.129
26	58	12	3	0.00013	0.129
26	58	12	4	0.00013	0.129
29	7	5	3	0.00013	0.129
29	7	5	14	0.00013	0.129
29	7	7	1	0.00013	0.129
29	7	7	3	0.00013	0.129
29	7	7	7	0.00026	0.241
29	7	7	9	0.00013	0.129
29	7	7	15	0.00013	0.129
29	7	15	3	0.00013	0.129
29	7	15	14	0.00013	0.129
29	7	16	7	0.00013	0.129
29	7	16	9	0.00013	0.129
29	14	6	1	0.00013	0.129
29	14	6	4	0.00013	0.129
29	14	6	11	0.00013	0.129
29	14	6	13	0.00013	0.129
29	14	8	1	0.00013	0.129
29	14	8	4	0.00026	0.241
29	14	8	7	0.00013	0.129
29	14	8	11	0.00013	0.129
29	14	8	13	0.00013	0.129
29	14	16	4	0.00013	0.129
29	14	16	7	0.00013	0.129
29	18	2	3	0.00013	0.129
29	18	2	11	0.00013	0.129
29	18	4	11	0.00013	0.129
29	18	4	16	0.00013	0.129
29	18	5	3	0.00066	0.549
29	18	5	7	0.00026	0.241
29	18	5	8	0.00013	0.129
29	18	5	11	0.00026	0.241
29	18	5	12	0.00013	0.129
29	18	5	14	0.00013	0.129
29	18	6	3	0.00013	0.129
29	18	6	8	0.00013	0.129
29	18	7	14	0.00013	0.129
29	18	7	16	0.00013	0.129
29	18	12	3	0.00013	0.129
29	18	12	11	0.00026	0.241
29	18	12	16	0.00013	0.129

29	18	15	3	0.00013	0.129
29	18	15	14	0.00013	0.129
29	18	16	3	0.00013	0.129
29	18	16	7	0.00026	0.241
29	18	16	12	0.00013	0.129
29	18	16	14	0.00013	0.129
29	18	16	16	0.00013	0.129
29	27	2	3	0.00013	0.129
29	27	2	11	0.00013	0.129
29	27	5	3	0.00013	0.129
29	27	5	11	0.00013	0.129
29	35	4	1	0.00026	0.241
29	35	4	7	0.00013	0.129
29	35	4	8	0.00013	0.129
29	35	4	11	0.00013	0.129
29	35	4	16	0.00013	0.129
29	35	7	1	0.00013	0.129
29	35	7	8	0.00013	0.129
29	35	12	11	0.00013	0.129
29	35	12	16	0.00013	0.129
29	35	16	1	0.00013	0.129
29	35	16	7	0.00013	0.129
29	39	5	3	0.00013	0.129
29	39	5	11	0.00013	0.129
29	39	7	7	0.00013	0.129
29	39	7	16	0.00013	0.129
29	39	12	3	0.00013	0.129
29	39	12	11	0.00013	0.129
29	39	16	7	0.00013	0.129
29	39	16	16	0.00013	0.129
29	44	4	1	0.00013	0.129
29	44	4	4	0.00013	0.129
29	44	4	7	0.00013	0.129
29	44	4	13	0.00013	0.129
29	44	5	3	0.00013	0.129
29	44	5	7	0.00026	0.241
29	44	5	12	0.00013	0.129
29	44	6	4	0.00013	0.129
29	44	6	13	0.00013	0.129
29	44	7	7	0.00026	0.241
29	44	7	9	0.00013	0.129
29	44	7	14	0.00013	0.129
29	44	7	16	0.00026	0.241
29	44	8	4	0.00013	0.129
29	44	8	7	0.00013	0.129
29	44	16	1	0.00013	0.129
29	44	16	3	0.00013	0.129
29	44	16	4	0.00013	0.129

29	44	16	7	0.00079	0.643
29	44	16	9	0.00013	0.129
29	44	16	12	0.00013	0.129
29	44	16	14	0.00013	0.129
29	44	16	16	0.00026	0.241
29	45	3	4	0.00013	0.129
29	45	4	4	0.00013	0.129
29	45	4	13	0.00013	0.129
29	45	6	1	0.00013	0.129
29	45	6	4	0.00118	0.914
29	45	6	11	0.00013	0.129
29	45	6	13	0.00026	0.241
29	45	6	15	0.00013	0.129
29	45	6	16	0.00013	0.129
29	45	7	4	0.00026	0.241
29	45	7	15	0.00013	0.129
29	45	7	16	0.00013	0.129
29	45	8	1	0.00013	0.129
29	45	8	4	0.00013	0.129
29	45	8	11	0.00013	0.129
29	45	8	13	0.00013	0.129
29	49	6	4	0.00013	0.129
29	49	6	15	0.00013	0.129
29	49	7	4	0.00013	0.129
29	49	7	9	0.00013	0.129
29	49	7	15	0.00026	0.241
29	49	16	9	0.00013	0.129
29	49	16	15	0.00013	0.129
29	50	5	3	0.00013	0.129
29	50	5	8	0.00013	0.129
29	50	6	3	0.00013	0.129
29	50	6	8	0.00013	0.129
29	52	7	9	0.00013	0.129
29	52	7	15	0.00013	0.129
29	52	16	9	0.00013	0.129
29	52	16	15	0.00013	0.129
29	55	3	4	0.00013	0.129
29	55	6	4	0.00013	0.129
29	58	4	1	0.00013	0.129
29	58	4	8	0.00013	0.129
29	58	6	4	0.00013	0.129
29	58	6	16	0.00013	0.129
29	58	7	1	0.00026	0.241
29	58	7	3	0.00013	0.129
29	58	7	4	0.00013	0.129
29	58	7	7	0.00013	0.129
29	58	7	8	0.00013	0.129
29	58	7	15	0.00013	0.129

29	58	7	16	0.00013	0.129
30	7	5	1	0.00026	0.241
30	7	5	3	0.00092	0.735
30	7	5	4	0.00013	0.129
30	7	5	11	0.00013	0.129
30	7	5	14	0.00013	0.129
30	7	5	15	0.00013	0.129
30	7	5	16	0.00013	0.129
30	7	7	1	0.00026	0.241
30	7	7	3	0.00066	0.549
30	7	7	8	0.00013	0.129
30	7	7	11	0.00013	0.129
30	7	7	15	0.00013	0.129
30	7	7	16	0.00026	0.241
30	7	12	8	0.00013	0.129
30	7	12	16	0.00013	0.129
30	7	15	3	0.00026	0.241
30	7	15	4	0.00013	0.129
30	7	15	14	0.00013	0.129
30	8	5	3	0.00053	0.453
30	8	5	11	0.00013	0.129
30	8	5	16	0.00013	0.129
30	8	6	3	0.00013	0.129
30	8	6	7	0.00013	0.129
30	8	7	3	0.00066	0.549
30	8	7	4	0.00013	0.129
30	8	7	7	0.00013	0.129
30	8	7	11	0.00013	0.129
30	8	7	16	0.00013	0.129
30	8	15	4	0.00013	0.129
30	13	4	4	0.00013	0.129
30	13	4	7	0.00013	0.129
30	13	4	11	0.00039	0.345
30	13	4	16	0.00013	0.129
30	13	5	3	0.00053	0.453
30	13	5	7	0.00053	0.453
30	13	6	1	0.00013	0.129
30	13	6	3	0.00053	0.453
30	13	6	4	0.00039	0.345
30	13	6	7	0.00105	0.825
30	13	6	11	0.00053	0.453
30	13	6	13	0.00013	0.129
30	13	6	16	0.00039	0.345
30	13	7	3	0.00013	0.129
30	13	7	4	0.00026	0.241
30	13	7	7	0.00039	0.345
30	13	7	16	0.00026	0.241
30	13	12	11	0.00013	0.129
30	13	12	13	0.00013	0.129
30	13	15	1	0.00013	0.129
30	13	15	7	0.00013	0.129
30	14	4	1	0.00013	0.129
30	14	4	4	0.00026	0.241
30	14	4	11	0.00013	0.129
30	14	5	1	0.00079	0.643
30	14	5	3	0.00105	0.825
30	14	5	7	0.00039	0.345
30	14	5	8	0.00013	0.129
30	14	5	15	0.00013	0.129
30	14	6	1	0.00013	0.129
30	14	6	4	0.00013	0.129
30	14	7	7	0.00013	0.129
30	14	7	16	0.00013	0.129
30	14	8	1	0.00092	0.735
30	14	8	3	0.00105	0.825
30	14	8	4	0.00039	0.345
30	14	8	7	0.00053	0.453
30	14	8	8	0.00013	0.129
30	14	8	11	0.00013	0.129
30	14	8	16	0.00013	0.129
30	14	12	1	0.00013	0.129
30	14	12	15	0.00013	0.129
30	15	5	3	0.00026	0.241
30	15	5	4	0.00013	0.129
30	15	5	11	0.00026	0.241
30	15	5	13	0.00013	0.129
30	15	7	3	0.00013	0.129
30	15	7	4	0.00013	0.129
30	15	7	11	0.00013	0.129
30	15	7	13	0.00013	0.129
30	15	12	3	0.00013	0.129
30	15	12	4	0.00013	0.129
30	15	12	10	0.00013	0.129
30	15	12	11	0.00013	0.129
30	15	16	4	0.00013	0.129
30	15	16	10	0.00013	0.129
30	18	1	3	0.00039	0.345
30	18	1	11	0.00013	0.129
30	18	1	16	0.00026	0.241
30	18	2	3	0.00118	0.914
30	18	2	7	0.00013	0.129
30	18	2	10	0.00013	0.129
30	18	2	11	0.00026	0.241
30	18	2	14	0.00013	0.129
30	18	2	16	0.00079	0.643
30	18	3	3	0.00105	0.825
30	18	3	7	0.00013	0.129
30	18	3	11	0.00013	0.129
30	18	3	13	0.00013	0.129
30	18	3	14	0.00026	0.241
30	18	3	15	0.00013	0.129
30	18	3	16	0.00013	0.129
30	18	4	1	0.00039	0.345
30	18	4	3	0.00236	1.663
30	18	4	4	0.00039	0.345
30	18	4	8	0.00026	0.241
30	18	4	11	0.00105	0.825
30	18	4	13	0.00039	0.345
30	18	4	14	0.00013	0.129
30	18	4	15	0.00013	0.129
30	18	4	16	0.00066	0.549
30	18	5	1	0.00171	1.26
30	18	5	3	0.02023	9.893
30	18	5	4	0.00236	1.663
30	18	5	7	0.00184	1.343
30	18	5	8	0.00118	0.914
30	18	5	10	0.00013	0.129
30	18	5	11	0.00447	2.864
30	18	5	12	0.00039	0.345
30	18	5	13	0.00171	1.26
30	18	5	14	0.00079	0.643
30	18	5	15	0.00092	0.735
30	18	5	16	0.00473	3.004
30	18	6	3	0.00145	1.093
30	18	6	4	0.00026	0.241
30	18	6	7	0.00066	0.549
30	18	6	8	0.00013	0.129
30	18	6	11	0.00026	0.241
30	18	7	1	0.00039	0.345
30	18	7	3	0.00552	3.421
30	18	7	4	0.00158	1.177
30	18	7	10	0.00013	0.129
30	18	7	11	0.00105	0.825
30	18	7	13	0.00039	0.345
30	18	7	15	0.00026	0.241
30	18	7	16	0.00263	1.825
30	18	8	1	0.00066	0.549
30	18	8	3	0.00105	0.825
30	18	8	7	0.00039	0.345
30	18	8	8	0.00013	0.129
30	18	12	1	0.00013	0.129
30	18	12	3	0.00118	0.914
30	18	12	10	0.00013	0.129
30	18	12	11	0.00079	0.643
30	18	12	14	0.00013	0.129
30	18	12	15	0.00026	0.241
30	18	12	16	0.00039	0.345
30	18	14	3	0.00026	0.241
30	18	14	4	0.00013	0.129
30	18	14	11	0.00013	0.129
30	18	15	3	0.00092	0.735
30	18	15	4	0.00053	0.453
30	18	15	11	0.00013	0.129
30	18	15	12	0.00013	0.129
30	18	15	14	0.00013	0.129
30	18	15	16	0.00026	0.241
30	18	16	1	0.00013	0.129
30	18	16	3	0.00053	0.453
30	18	16	7	0.00026	0.241
30	18	16	12	0.00013	0.129
30	18	16	13	0.00013	0.129
30	18	16	16	0.00013	0.129
30	18	17	8	0.00026	0.241
30	18	17	13	0.00026	0.241
30	27	1	3	0.00013	0.129
30	27	1	16	0.00013	0.129
30	27	2	3	0.00026	0.241
30	27	2	16	0.00026	0.241
30	27	5	3	0.00039	0.345
30	27	5	16	0.00039	0.345
30	35	3	1	0.00013	0.129
30	35	3	3	0.00039	0.345
30	35	3	15	0.00013	0.129
30	35	3	16	0.00026	0.241
30	35	4	1	0.00066	0.549
30	35	4	3	0.00197	1.424
30	35	4	4	0.00092	0.735
30	35	4	7	0.00026	0.241
30	35	4	8	0.00013	0.129
30	35	4	11	0.00184	1.343
30	35	4	12	0.00013	0.129
30	35	4	13	0.00026	0.241
30	35	4	14	0.00026	0.241
30	35	4	15	0.00013	0.129
30	35	4	16	0.00118	0.914
30	35	5	1	0.00039	0.345
30	35	5	3	0.00276	1.902
30	35	5	4	0.00039	0.345
30	35	5	8	0.00013	0.129
30	35	5	11	0.00092	0.735
30	35	5	13	0.00026	0.241
30	35	5	14	0.00039	0.345
30	35	5	15	0.00026	0.241
30	35	5	16	0.00092	0.735
30	35	6	4	0.00026	0.241
30	35	6	7	0.00026	0.241
30	35	6	10	0.00013	0.129
30	35	6	11	0.00066	0.549
30	35	6	16	0.00013	0.129
30	35	7	1	0.00013	0.129
30	35	7	4	0.00026	0.241
30	35	7	10	0.00013	0.129
30	35	7	16	0.00026	0.241
30	35	8	4	0.00013	0.129
30	35	8	11	0.00013	0.129
30	35	12	3	0.00013	0.129
30	35	12	14	0.00013	0.129
30	35	15	3	0.00013	0.129
30	35	15	16	0.00013	0.129
30	35	16	3	0.00013	0.129
30	35	16	11	0.00013	0.129
30	35	16	12	0.00013	0.129
30	35	16	16	0.00013	0.129
30	35	17	4	0.00013	0.129
30	35	17	16	0.00013	0.129
30	37	2	3	0.00013	0.129
30	37	2	16	0.00013	0.129
30	37	5	3	0.00039	0.345
30	37	5	4	0.00013	0.129
30	37	5	11	0.00026	0.241
30	37	6	3	0.00053	0.453
30	37	6	4	0.00026	0.241
30	37	6	10	0.00013	0.129
30	37	6	11	0.00026	0.241
30	37	6	16	0.00013	0.129
30	37	15	4	0.00013	0.129
30	37	15	10	0.00013	0.129
30	38	5	1	0.00013	0.129
30	38	5	3	0.00013	0.129
30	38	5	15	0.00013	0.129
30	38	5	16	0.00013	0.129
30	38	6	11	0.00013	0.129
30	38	6	13	0.00013	0.129
30	38	7	12	0.00013	0.129
30	38	7	13	0.00013	0.129
30	38	12	1	0.00013	0.129
30	38	12	3	0.00013	0.129
30	38	12	11	0.00013	0.129
30	38	12	12	0.00013	0.129
30	38	12	13	0.00026	0.241
30	38	12	15	0.00013	0.129
30	38	12	16	0.00013	0.129
30	39	5	3	0.00026	0.241
30	39	5	11	0.00013	0.129
30	39	5	13	0.00013	0.129
30	39	7	3	0.00026	0.241
30	39	7	8	0.00013	0.129
30	39	7	13	0.00013	0.129
30	39	7	16	0.00026	0.241
30	39	12	3	0.00013	0.129
30	39	12	8	0.00013	0.129
30	39	12	11	0.00013	0.129
30	39	12	16	0.00013	0.129
30	39	15	3	0.00013	0.129
30	39	15	16	0.00013	0.129
30	40	2	3	0.00053	0.453
30	40	2	11	0.00013	0.129
30	40	2	15	0.00013	0.129
30	40	2	16	0.00053	0.453
30	40	3	3	0.00013	0.129
30	40	3	13	0.00013	0.129
30	40	5	3	0.00053	0.453
30	40	5	11	0.00013	0.129
30	40	5	13	0.00013	0.129
30	40	5	16	0.00026	0.241
30	40	6	3	0.00013	0.129
30	40	6	16	0.00013	0.129
30	40	7	15	0.00013	0.129
30	40	7	16	0.00013	0.129
30	41	1	4	0.00013	0.129
30	41	1	12	0.00013	0.129
30	41	4	4	0.00013	0.129
30	41	4	16	0.00013	0.129
30	41	5	8	0.00026	0.241
30	41	5	13	0.00026	0.241
30	41	6	4	0.00013	0.129
30	41	6	10	0.00013	0.129
30	41	7	4	0.00026	0.241
30	41	7	7	0.00013	0.129
30	41	7	10	0.00013	0.129
30	41	17	4	0.00039	0.345
30	41	17	7	0.00013	0.129
30	41	17	8	0.00026	0.241
30	41	17	12	0.00013	0.129
30	41	17	13	0.00026	0.241
30	41	17	16	0.00013	0.129
30	44	2	3	0.00013	0.129
30	44	2	7	0.00013	0.129

30	44	2	10	0.00013	0.129
30	44	2	14	0.00013	0.129
30	44	2	16	0.00026	0.241
30	44	4	3	0.00013	0.129
30	44	4	4	0.00013	0.129
30	44	4	11	0.00013	0.129
30	44	4	13	0.00013	0.129
30	44	4	14	0.00013	0.129
30	44	5	3	0.00131	1.000
30	44	5	4	0.00013	0.129
30	44	5	7	0.00039	0.345
30	44	5	11	0.00026	0.241
30	44	5	12	0.00039	0.345
30	44	5	13	0.00026	0.241
30	44	5	14	0.00026	0.241
30	44	5	16	0.00013	0.129
30	44	6	7	0.00013	0.129
30	44	6	11	0.00013	0.129
30	44	6	16	0.00013	0.129
30	44	12	10	0.00013	0.129
30	44	12	16	0.00013	0.129
30	44	15	3	0.00013	0.129
30	44	15	12	0.00013	0.129
30	44	16	3	0.00013	0.129
30	44	16	7	0.00026	0.241
30	44	16	12	0.00013	0.129
30	45	4	11	0.00013	0.129
30	45	4	12	0.00013	0.129
30	45	5	3	0.00013	0.129
30	45	6	1	0.00013	0.129
30	45	6	3	0.00013	0.129
30	45	6	4	0.00039	0.345
30	45	6	7	0.00013	0.129
30	45	6	16	0.00013	0.129
30	45	7	4	0.00013	0.129
30	45	7	16	0.00013	0.129
30	45	8	1	0.00013	0.129
30	45	8	4	0.00013	0.129
30	45	16	11	0.00013	0.129
30	45	16	12	0.00013	0.129
30	49	2	11	0.00013	0.129
30	49	5	3	0.00131	1.000
30	49	5	4	0.00092	0.735
30	49	5	11	0.00026	0.241
30	49	5	13	0.00013	0.129
30	49	6	4	0.00013	0.129
30	49	6	7	0.00013	0.129
30	49	7	3	0.00131	1.000
30	49	7	4	0.00118	0.914
30	49	7	7	0.00026	0.241
30	49	7	11	0.00039	0.345
30	49	7	12	0.00013	0.129
30	49	7	13	0.00026	0.241
30	49	12	12	0.00013	0.129
30	49	12	13	0.00013	0.129
30	49	17	4	0.00013	0.129
30	49	17	7	0.00013	0.129
30	50	4	1	0.00013	0.129
30	50	4	3	0.00013	0.129
30	50	4	4	0.00013	0.129
30	50	4	7	0.00013	0.129
30	50	4	11	0.00026	0.241
30	50	5	3	0.00079	0.643
30	50	5	4	0.00013	0.129
30	50	5	7	0.00026	0.241
30	50	5	8	0.00013	0.129
30	50	5	11	0.00013	0.129
30	50	5	13	0.00013	0.129
30	50	6	3	0.00053	0.453
30	50	6	4	0.00026	0.241
30	50	6	7	0.00053	0.453
30	50	6	8	0.00013	0.129
30	50	6	11	0.00013	0.129
30	50	8	1	0.00013	0.129
30	50	8	4	0.00013	0.129
30	51	1	3	0.00026	0.241
30	51	1	4	0.00013	0.129
30	51	1	11	0.00013	0.129
30	51	1	12	0.00013	0.129
30	51	1	16	0.00013	0.129
30	51	2	3	0.00039	0.345
30	51	2	4	0.00026	0.241
30	51	2	7	0.00013	0.129
30	51	2	10	0.00013	0.129
30	51	2	11	0.00026	0.241
30	51	2	13	0.00013	0.129
30	51	2	16	0.00013	0.129
30	51	4	4	0.00013	0.129
30	51	4	13	0.00013	0.129
30	51	5	1	0.00013	0.129
30	51	5	3	0.00158	1.177
30	51	5	4	0.00013	0.129
30	51	5	7	0.00013	0.129
30	51	5	11	0.00079	0.643
30	51	5	13	0.00013	0.129
30	51	5	16	0.00026	0.241
30	51	6	1	0.00013	0.129
30	51	6	7	0.00013	0.129
30	51	7	3	0.00026	0.241
30	51	7	11	0.00026	0.241
30	51	7	16	0.00013	0.129
30	51	12	1	0.00013	0.129
30	51	12	3	0.00026	0.241
30	51	12	11	0.00013	0.129
30	51	14	3	0.00026	0.241
30	51	14	4	0.00013	0.129
30	51	14	11	0.00013	0.129
30	51	15	1	0.00013	0.129
30	51	15	3	0.00026	0.241
30	51	15	7	0.00013	0.129
30	51	15	11	0.00013	0.129
30	51	15	16	0.00013	0.129
30	51	16	3	0.00013	0.129
30	51	16	4	0.00013	0.129
30	51	16	10	0.00013	0.129
30	51	16	13	0.00013	0.129
30	51	17	4	0.00013	0.129
30	51	17	12	0.00013	0.129
30	52	1	3	0.00013	0.129
30	52	1	11	0.00013	0.129
30	52	5	3	0.00053	0.453
30	52	5	11	0.00013	0.129
30	52	5	15	0.00039	0.345
30	52	12	3	0.00026	0.241
30	52	12	15	0.00026	0.241
30	53	2	4	0.00013	0.129
30	53	2	13	0.00013	0.129
30	53	4	4	0.00013	0.129
30	53	4	13	0.00013	0.129
30	55	1	3	0.00013	0.129
30	55	1	4	0.00013	0.129
30	55	1	11	0.00026	0.241
30	55	3	3	0.00039	0.345
30	55	3	11	0.00013	0.129
30	55	3	14	0.00026	0.241
30	55	5	3	0.00053	0.453
30	55	5	11	0.00026	0.241
30	55	5	14	0.00026	0.241
30	55	15	4	0.00013	0.129
30	55	15	11	0.00013	0.129
30	57	2	7	0.00013	0.129
30	57	2	16	0.00013	0.129
30	57	5	11	0.00013	0.129
30	57	6	7	0.00026	0.241
30	57	6	10	0.00013	0.129
30	57	6	16	0.00013	0.129
30	57	12	11	0.00013	0.129
30	57	16	7	0.00013	0.129
30	57	16	10	0.00013	0.129
30	58	2	15	0.00013	0.129
30	58	2	16	0.00013	0.129
30	58	3	1	0.00013	0.129
30	58	3	3	0.00013	0.129
30	58	3	7	0.00013	0.129
30	58	3	16	0.00013	0.129
30	58	4	1	0.00026	0.241
30	58	4	3	0.00013	0.129
30	58	4	8	0.00013	0.129
30	58	4	16	0.00026	0.241
30	58	5	3	0.00184	1.343
30	58	5	7	0.00013	0.129
30	58	5	8	0.00013	0.129
30	58	5	10	0.00013	0.129
30	58	5	11	0.00026	0.241
30	58	5	15	0.00013	0.129
30	58	5	16	0.00145	1.093
30	58	6	4	0.00026	0.241
30	58	6	7	0.00013	0.129
30	58	6	16	0.00039	0.345
30	58	7	1	0.00013	0.129
30	58	7	3	0.00158	1.177
30	58	7	4	0.00039	0.345
30	58	7	7	0.00026	0.241
30	58	7	10	0.00013	0.129
30	58	7	11	0.00026	0.241
30	58	7	15	0.00026	0.241
30	58	7	16	0.00236	1.663
30	58	8	7	0.00013	0.129
30	58	8	16	0.00013	0.129
30	58	15	4	0.00013	0.129
30	58	15	16	0.00013	0.129
30	73	1	4	0.00013	0.129
30	73	1	11	0.00013	0.129
30	73	5	3	0.00026	0.241
30	73	5	4	0.00026	0.241
30	73	6	4	0.00013	0.129
30	73	6	10	0.00013	0.129
30	73	7	4	0.00039	0.345
30	73	7	16	0.00026	0.241
30	73	15	3	0.00026	0.241
30	73	15	4	0.00092	0.735
30	73	15	10	0.00013	0.129
30	73	15	11	0.00013	0.129
30	73	15	16	0.00026	0.241
30	78	2	4	0.00013	0.129
30	78	2	10	0.00013	0.129
30	78	5	1	0.00013	0.129
30	78	5	3	0.00013	0.129
30	78	6	7	0.00013	0.129
30	78	6	10	0.00013	0.129
30	78	12	4	0.00013	0.129
30	78	12	10	0.00013	0.129
30	78	16	1	0.00013	0.129
30	78	16	3	0.00013	0.129
30	78	16	4	0.00026	0.241
30	78	16	7	0.00013	0.129
30	78	16	10	0.00039	0.345
31	8	6	3	0.00013	0.129
31	8	6	4	0.00013	0.129
31	8	7	3	0.00013	0.129
31	8	7	4	0.00013	0.129
31	15	2	4	0.00013	0.129
31	15	2	11	0.00013	0.129
31	15	5	3	0.00013	0.129
31	15	5	11	0.00013	0.129
31	15	7	3	0.00013	0.129
31	15	7	4	0.00013	0.129
31	15	7	11	0.00026	0.241
31	18	4	3	0.00026	0.241
31	18	4	4	0.00013	0.129
31	18	4	13	0.00013	0.129
31	18	5	3	0.00039	0.345
31	18	5	4	0.00013	0.129
31	18	5	11	0.00013	0.129
31	18	5	13	0.00013	0.129
31	18	7	1	0.00013	0.129
31	18	7	3	0.00013	0.129
31	18	7	11	0.00026	0.241
31	18	15	1	0.00013	0.129
31	18	15	11	0.00013	0.129
31	35	4	3	0.00013	0.129
31	35	4	4	0.00013	0.129
31	35	4	7	0.00013	0.129
31	35	4	11	0.00026	0.241
31	35	4	12	0.00013	0.129
31	35	5	3	0.00013	0.129
31	35	5	4	0.00013	0.129
31	35	12	11	0.00013	0.129
31	35	12	12	0.00013	0.129
31	35	14	7	0.00013	0.129
31	35	14	11	0.00013	0.129
31	37	6	11	0.00013	0.129
31	37	6	12	0.00013	0.129
31	37	7	11	0.00013	0.129
31	37	7	12	0.00013	0.129
31	38	4	11	0.00013	0.129
31	38	4	12	0.00013	0.129
31	38	12	11	0.00013	0.129
31	38	12	12	0.00013	0.129
31	44	4	3	0.00013	0.129
31	44	4	4	0.00013	0.129
31	44	4	13	0.00066	0.549
31	44	4	16	0.00013	0.129
31	44	5	3	0.00013	0.129
31	44	5	13	0.00013	0.129
31	44	6	4	0.00013	0.129
31	44	6	13	0.00013	0.129
31	44	7	13	0.00013	0.129
31	44	7	16	0.00013	0.129
31	45	4	4	0.00013	0.129
31	45	4	13	0.00013	0.129
31	45	6	3	0.00013	0.129
31	45	6	4	0.00026	0.241
31	45	6	13	0.00013	0.129
31	45	7	3	0.00013	0.129
31	45	7	4	0.00013	0.129
31	51	2	4	0.00026	0.241
31	51	2	11	0.00026	0.241
31	51	4	7	0.00013	0.129
31	51	4	11	0.00013	0.129
31	51	7	1	0.00013	0.129
31	51	7	4	0.00013	0.129
31	51	7	11	0.00026	0.241
31	51	14	7	0.00013	0.129
31	51	14	11	0.00013	0.129
31	51	15	1	0.00013	0.129
31	51	15	11	0.00013	0.129
31	58	4	13	0.00013	0.129
31	58	4	16	0.00013	0.129
31	58	6	11	0.00013	0.129
31	58	6	12	0.00013	0.129
31	58	7	11	0.00013	0.129
31	58	7	12	0.00013	0.129
31	58	7	13	0.00013	0.129
31	58	7	16	0.00013	0.129
32	7	2	16	0.00013	0.129
32	7	5	3	0.00026	0.241
32	7	5	4	0.00013	0.129

32	7	5	16	0.00013	0.129
32	7	7	3	0.00026	0.241
32	7	7	4	0.00013	0.129
32	7	7	16	0.00026	0.241
32	7	15	7	0.00013	0.129
32	7	15	16	0.00013	0.129
32	8	2	3	0.00013	0.129
32	8	2	8	0.00026	0.241
32	8	2	11	0.00013	0.129
32	8	7	3	0.00039	0.345
32	8	7	4	0.00013	0.129
32	8	7	8	0.00039	0.345
32	8	7	11	0.00013	0.129
32	8	7	16	0.00026	0.241
32	13	2	11	0.00013	0.129
32	13	2	16	0.00013	0.129
32	13	6	3	0.00013	0.129
32	13	6	7	0.00026	0.241
32	13	6	11	0.00013	0.129
32	13	6	16	0.00013	0.129
32	13	7	3	0.00013	0.129
32	13	7	7	0.00013	0.129
32	14	2	4	0.00013	0.129
32	14	2	7	0.00013	0.129
32	14	3	1	0.00013	0.129
32	14	3	4	0.00013	0.129
32	14	3	16	0.00026	0.241
32	14	4	1	0.00013	0.129
32	14	4	4	0.00013	0.129
32	14	7	1	0.00026	0.241
32	14	7	7	0.00013	0.129
32	14	7	11	0.00013	0.129
32	14	7	16	0.00026	0.241
32	14	8	1	0.00053	0.453
32	14	8	3	0.00013	0.129
32	14	8	4	0.00053	0.453
32	14	8	7	0.00053	0.453
32	14	8	11	0.00026	0.241
32	14	8	15	0.00013	0.129
32	14	8	16	0.00066	0.549
32	14	16	4	0.00013	0.129
32	14	16	7	0.00026	0.241
32	14	16	11	0.00013	0.129
32	14	16	15	0.00013	0.129
32	14	16	16	0.00013	0.129
32	14	17	3	0.00013	0.129
32	15	3	1	0.00013	0.129
32	15	3	11	0.00013	0.129

32	15	4	1	0.00013	0.129
32	15	4	4	0.00013	0.129
32	15	7	1	0.00013	0.129
32	15	7	11	0.00013	0.129
32	15	8	1	0.00013	0.129
32	15	8	4	0.00013	0.129
32	18	1	3	0.00026	0.241
32	18	1	11	0.00013	0.129
32	18	1	16	0.00013	0.129
32	18	2	3	0.00026	0.241
32	18	2	4	0.00013	0.129
32	18	2	11	0.00026	0.241
32	18	2	13	0.00013	0.129
32	18	2	16	0.00092	0.735
32	18	3	3	0.00039	0.345
32	18	3	15	0.00013	0.129
32	18	3	16	0.00013	0.129
32	18	4	1	0.00013	0.129
32	18	4	4	0.00026	0.241
32	18	4	10	0.00013	0.129
32	18	4	11	0.00026	0.241
32	18	4	16	0.00026	0.241
32	18	5	1	0.00013	0.129
32	18	5	3	0.00276	1.902
32	18	5	4	0.00026	0.241
32	18	5	7	0.00026	0.241
32	18	5	11	0.00053	0.453
32	18	5	13	0.00013	0.129
32	18	5	15	0.00026	0.241
32	18	5	16	0.00145	1.093
32	18	6	1	0.00013	0.129
32	18	6	3	0.00079	0.643
32	18	6	4	0.00026	0.241
32	18	6	7	0.00026	0.241
32	18	6	11	0.00013	0.129
32	18	6	16	0.00013	0.129
32	18	7	1	0.00053	0.453
32	18	7	3	0.00171	1.26
32	18	7	4	0.00092	0.735
32	18	7	7	0.00026	0.241
32	18	7	10	0.00013	0.129
32	18	7	11	0.00066	0.549
32	18	7	13	0.00013	0.129
32	18	7	14	0.00013	0.129
32	18	7	16	0.00289	1.978
32	18	8	1	0.00026	0.241
32	18	8	7	0.00013	0.129
32	18	8	11	0.00013	0.129

32	18	8	16	0.00026	0.241
32	18	12	1	0.00013	0.129
32	18	12	3	0.00026	0.241
32	18	12	11	0.00039	0.345
32	18	12	13	0.00013	0.129
32	18	12	15	0.00013	0.129
32	18	12	16	0.00039	0.345
32	18	14	4	0.00013	0.129
32	18	14	11	0.00013	0.129
32	18	15	3	0.00026	0.241
32	18	15	4	0.00013	0.129
32	18	15	11	0.00013	0.129
32	18	15	16	0.00066	0.549
32	18	16	14	0.00013	0.129
32	18	16	16	0.00013	0.129
32	27	1	1	0.00013	0.129
32	27	1	3	0.00013	0.129
32	27	1	11	0.00013	0.129
32	27	1	12	0.00026	0.241
32	27	1	15	0.00013	0.129
32	27	1	16	0.00026	0.241
32	27	2	1	0.00013	0.129
32	27	2	3	0.00013	0.129
32	27	2	4	0.00013	0.129
32	27	2	8	0.00026	0.241
32	27	2	10	0.00013	0.129
32	27	2	11	0.00013	0.129
32	27	2	16	0.00053	0.453
32	27	4	1	0.00013	0.129
32	27	4	11	0.00013	0.129
32	27	5	3	0.00013	0.129
32	27	5	16	0.00013	0.129
32	27	7	1	0.00013	0.129
32	27	7	3	0.00013	0.129
32	27	7	4	0.00013	0.129
32	27	7	8	0.00026	0.241
32	27	7	10	0.00013	0.129
32	27	7	12	0.00013	0.129
32	27	7	16	0.00053	0.453
32	27	8	15	0.00013	0.129
32	27	8	16	0.00013	0.129
32	27	12	12	0.00013	0.129
32	27	12	15	0.00013	0.129
32	27	15	11	0.00013	0.129
32	27	15	16	0.00013	0.129
32	27	16	15	0.00013	0.129
32	27	16	16	0.00013	0.129
32	35	1	1	0.00013	0.129

32	35	1	11	0.00013	0.129
32	35	2	3	0.00013	0.129
32	35	2	10	0.00013	0.129
32	35	2	11	0.00026	0.241
32	35	2	16	0.00026	0.241
32	35	3	3	0.00039	0.345
32	35	3	15	0.00013	0.129
32	35	3	16	0.00013	0.129
32	35	4	1	0.00053	0.453
32	35	4	4	0.00039	0.345
32	35	4	7	0.00026	0.241
32	35	4	10	0.00013	0.129
32	35	4	11	0.00053	0.453
32	35	4	16	0.00092	0.735
32	35	5	3	0.00053	0.453
32	35	5	4	0.00013	0.129
32	35	5	11	0.00013	0.129
32	35	5	15	0.00013	0.129
32	35	5	16	0.00026	0.241
32	35	7	1	0.00026	0.241
32	35	7	4	0.00013	0.129
32	35	7	7	0.00026	0.241
32	35	7	10	0.00013	0.129
32	35	7	11	0.00013	0.129
32	35	7	16	0.00105	0.825
32	35	8	7	0.00013	0.129
32	35	8	11	0.00013	0.129
32	35	12	4	0.00013	0.129
32	35	12	11	0.00013	0.129
32	35	12	16	0.00026	0.241
32	35	15	1	0.00013	0.129
32	35	15	3	0.00026	0.241
32	35	15	4	0.00053	0.453
32	35	15	7	0.00013	0.129
32	35	15	10	0.00013	0.129
32	35	15	11	0.00039	0.345
32	35	15	16	0.00171	1.26
32	35	16	7	0.00013	0.129
32	35	16	11	0.00026	0.241
32	37	5	1	0.00013	0.129
32	37	5	3	0.00013	0.129
32	37	6	1	0.00013	0.129
32	37	6	3	0.00013	0.129
32	38	2	13	0.00013	0.129
32	38	2	16	0.00013	0.129
32	38	12	4	0.00013	0.129
32	38	12	13	0.00013	0.129
32	38	12	16	0.00026	0.241

32	38	15	4	0.00013	0.129
32	38	15	16	0.00013	0.129
32	39	2	1	0.00026	0.241
32	39	2	3	0.00013	0.129
32	39	2	11	0.00013	0.129
32	39	2	16	0.00013	0.129
32	39	7	1	0.00066	0.549
32	39	7	11	0.00039	0.345
32	39	7	16	0.00026	0.241
32	39	12	1	0.00013	0.129
32	39	12	3	0.00013	0.129
32	39	12	11	0.00013	0.129
32	39	15	1	0.00013	0.129
32	39	15	11	0.00013	0.129
32	40	2	16	0.00013	0.129
32	40	7	16	0.00013	0.129
32	41	1	4	0.00013	0.129
32	41	1	12	0.00013	0.129
32	41	7	3	0.00013	0.129
32	41	7	4	0.00013	0.129
32	41	8	3	0.00013	0.129
32	41	16	1	0.00013	0.129
32	41	16	3	0.00013	0.129
32	41	17	1	0.00013	0.129
32	41	17	3	0.00039	0.345
32	41	17	4	0.00026	0.241
32	41	17	12	0.00013	0.129
32	44	2	1	0.00013	0.129
32	44	2	15	0.00013	0.129
32	44	4	1	0.00013	0.129
32	44	4	15	0.00013	0.129
32	44	5	3	0.00026	0.241
32	44	5	11	0.00026	0.241
32	44	5	13	0.00013	0.129
32	44	5	14	0.00026	0.241
32	44	5	16	0.00013	0.129
32	44	7	3	0.00026	0.241
32	44	7	4	0.00013	0.129
32	44	7	7	0.00013	0.129
32	44	7	11	0.00013	0.129
32	44	7	13	0.00013	0.129
32	44	7	14	0.00013	0.129
32	44	7	16	0.00026	0.241
32	44	8	4	0.00013	0.129
32	44	8	7	0.00013	0.129
32	44	12	11	0.00013	0.129
32	44	14	4	0.00013	0.129
32	44	14	11	0.00013	0.129

32	44	16	4	0.00013	0.129
32	44	16	7	0.00013	0.129
32	44	16	14	0.00013	0.129
32	44	16	16	0.00013	0.129
32	45	3	11	0.00013	0.129
32	45	3	16	0.00013	0.129
32	45	7	11	0.00013	0.129
32	45	7	16	0.00013	0.129
32	49	1	8	0.00013	0.129
32	49	1	16	0.00013	0.129
32	49	6	3	0.00026	0.241
32	49	6	4	0.00026	0.241
32	49	7	3	0.00053	0.453
32	49	7	4	0.00066	0.549
32	49	7	8	0.00013	0.129
32	49	7	9	0.00013	0.129
32	49	7	15	0.00013	0.129
32	49	7	16	0.00013	0.129
32	49	16	9	0.00013	0.129
32	49	16	15	0.00013	0.129
32	49	17	3	0.00013	0.129
32	49	17	4	0.00013	0.129
32	50	5	3	0.00026	0.241
32	50	5	7	0.00026	0.241
32	50	6	3	0.00026	0.241
32	50	6	7	0.00053	0.453
32	50	6	16	0.00013	0.129
32	50	7	7	0.00013	0.129
32	50	7	16	0.00013	0.129
32	51	1	3	0.00013	0.129
32	51	1	4	0.00013	0.129
32	51	1	11	0.00013	0.129
32	51	1	12	0.00013	0.129
32	51	2	1	0.00013	0.129
32	51	2	4	0.00026	0.241
32	51	2	7	0.00013	0.129
32	51	2	8	0.00013	0.129
32	51	2	11	0.00053	0.453
32	51	2	16	0.00013	0.129
32	51	3	4	0.00013	0.129
32	51	3	16	0.00013	0.129
32	51	4	11	0.00013	0.129
32	51	5	3	0.00013	0.129
32	51	5	11	0.00013	0.129
32	51	7	1	0.00026	0.241
32	51	7	8	0.00013	0.129
32	51	7	11	0.00053	0.453
32	51	7	16	0.00013	0.129

32	51	8	4	0.00013	0.129
32	51	8	7	0.00013	0.129
32	51	15	1	0.00013	0.129
32	51	15	11	0.00013	0.129
32	51	16	1	0.00013	0.129
32	51	16	3	0.00013	0.129
32	51	16	4	0.00013	0.129
32	51	16	11	0.00013	0.129
32	51	16	16	0.00013	0.129
32	51	17	1	0.00013	0.129
32	51	17	3	0.00013	0.129
32	51	17	4	0.00013	0.129
32	51	17	12	0.00013	0.129
32	52	1	12	0.00013	0.129
32	52	1	15	0.00013	0.129
32	52	5	3	0.00013	0.129
32	52	5	15	0.00013	0.129
32	52	7	9	0.00013	0.129
32	52	7	15	0.00013	0.129
32	52	12	3	0.00013	0.129
32	52	12	12	0.00013	0.129
32	52	12	15	0.00026	0.241
32	52	16	9	0.00013	0.129
32	52	16	15	0.00013	0.129
32	53	2	1	0.00013	0.129
32	53	2	15	0.00013	0.129
32	53	4	1	0.00013	0.129
32	53	4	4	0.00013	0.129
32	53	4	15	0.00013	0.129
32	53	4	16	0.00013	0.129
32	53	7	4	0.00013	0.129
32	53	7	16	0.00013	0.129
32	55	3	1	0.00026	0.241
32	55	3	4	0.00026	0.241
32	55	3	11	0.00039	0.345
32	55	3	16	0.00079	0.643
32	55	7	1	0.00013	0.129
32	55	7	11	0.00039	0.345
32	55	7	16	0.00039	0.345
32	55	8	1	0.00013	0.129
32	55	8	4	0.00013	0.129
32	55	8	16	0.00026	0.241
32	55	16	4	0.00013	0.129
32	55	16	16	0.00013	0.129
32	56	1	8	0.00013	0.129
32	56	1	16	0.00013	0.129
32	56	7	8	0.00013	0.129
32	56	7	16	0.00013	0.129

32	57	4	7	0.00013	0.129
32	57	4	16	0.00013	0.129
32	57	7	7	0.00013	0.129
32	57	7	16	0.00013	0.129
32	58	1	12	0.00013	0.129
32	58	1	16	0.00013	0.129
32	58	2	8	0.00013	0.129
32	58	2	10	0.00013	0.129
32	58	2	16	0.00013	0.129
32	58	3	11	0.00013	0.129
32	58	3	16	0.00026	0.241
32	58	4	1	0.00013	0.129
32	58	4	7	0.00013	0.129
32	58	4	16	0.00026	0.241
32	58	5	3	0.00013	0.129
32	58	5	16	0.00013	0.129
32	58	6	3	0.00013	0.129
32	58	6	7	0.00026	0.241
32	58	6	16	0.00013	0.129
32	58	7	1	0.00026	0.241
32	58	7	3	0.00026	0.241
32	58	7	7	0.00039	0.345
32	58	7	8	0.00026	0.241
32	58	7	10	0.00013	0.129
32	58	7	11	0.00013	0.129
32	58	7	12	0.00013	0.129
32	58	7	16	0.00184	1.343
32	58	12	16	0.00013	0.129
32	58	15	16	0.00026	0.241
33	7	7	3	0.00013	0.129
33	7	7	4	0.00013	0.129
33	7	7	11	0.00026	0.241
33	7	8	3	0.00026	0.241
33	7	8	11	0.00026	0.241
33	7	15	3	0.00013	0.129
33	7	15	4	0.00013	0.129
33	7	15	11	0.00026	0.241
33	14	2	1	0.00039	0.345
33	14	2	3	0.00013	0.129
33	14	2	7	0.00013	0.129
33	14	2	16	0.00013	0.129
33	14	3	3	0.00026	0.241
33	14	3	4	0.00013	0.129
33	14	3	11	0.00026	0.241
33	14	3	16	0.00013	0.129
33	14	4	4	0.00013	0.129
33	14	4	11	0.00026	0.241
33	14	4	15	0.00013	0.129

33	14	5	1	0.00053	0.453
33	14	5	3	0.00066	0.549
33	14	5	7	0.00026	0.241
33	14	5	8	0.00013	0.129
33	14	5	16	0.00013	0.129
33	14	6	1	0.00039	0.345
33	14	6	3	0.00013	0.129
33	14	6	4	0.00026	0.241
33	14	6	7	0.00013	0.129
33	14	6	11	0.00013	0.129
33	14	7	1	0.00066	0.549
33	14	7	3	0.00039	0.345
33	14	7	4	0.00013	0.129
33	14	7	7	0.00013	0.129
33	14	7	11	0.00013	0.129
33	14	7	12	0.00013	0.129
33	14	7	13	0.00013	0.129
33	14	7	16	0.00066	0.549
33	14	8	1	0.00263	1.825
33	14	8	3	0.00197	1.424
33	14	8	4	0.00079	0.643
33	14	8	7	0.00092	0.735
33	14	8	8	0.00013	0.129
33	14	8	11	0.00105	0.825
33	14	8	12	0.00013	0.129
33	14	8	13	0.00039	0.345
33	14	8	15	0.00026	0.241
33	14	8	16	0.00145	1.093
33	14	12	1	0.00039	0.345
33	14	12	3	0.00013	0.129
33	14	12	4	0.00013	0.129
33	14	12	11	0.00013	0.129
33	14	12	13	0.00013	0.129
33	14	12	16	0.00013	0.129
33	14	14	13	0.00013	0.129
33	14	14	16	0.00013	0.129
33	14	15	3	0.00013	0.129
33	14	15	11	0.00013	0.129
33	14	16	15	0.00013	0.129
33	14	16	16	0.00013	0.129
33	14	17	3	0.00013	0.129
33	15	7	1	0.00013	0.129
33	15	7	13	0.00013	0.129
33	15	8	1	0.00013	0.129
33	15	8	13	0.00013	0.129
33	18	3	13	0.00013	0.129
33	18	3	16	0.00013	0.129
33	18	5	1	0.00053	0.453

33	18	5	3	0.00079	0.643
33	18	5	7	0.00026	0.241
33	18	5	8	0.00013	0.129
33	18	7	1	0.00013	0.129
33	18	7	3	0.00013	0.129
33	18	7	4	0.00013	0.129
33	18	7	11	0.00013	0.129
33	18	7	13	0.00013	0.129
33	18	7	16	0.00039	0.345
33	18	8	1	0.00066	0.549
33	18	8	3	0.00079	0.643
33	18	8	7	0.00026	0.241
33	18	8	8	0.00013	0.129
33	18	8	11	0.00013	0.129
33	18	8	16	0.00026	0.241
33	18	12	1	0.00013	0.129
33	18	12	11	0.00013	0.129
33	18	15	4	0.00013	0.129
33	18	15	11	0.00013	0.129
33	18	16	1	0.00013	0.129
33	18	16	3	0.00013	0.129
33	27	2	1	0.00013	0.129
33	27	2	3	0.00013	0.129
33	27	8	1	0.00013	0.129
33	27	8	3	0.00013	0.129
33	27	8	15	0.00013	0.129
33	27	8	16	0.00013	0.129
33	27	16	15	0.00013	0.129
33	27	16	16	0.00013	0.129
33	35	4	3	0.00013	0.129
33	35	4	4	0.00013	0.129
33	35	4	11	0.00039	0.345
33	35	4	15	0.00013	0.129
33	35	4	16	0.00013	0.129
33	35	6	11	0.00013	0.129
33	35	6	12	0.00013	0.129
33	35	6	16	0.00013	0.129
33	35	7	1	0.00013	0.129
33	35	7	3	0.00013	0.129
33	35	7	12	0.00026	0.241
33	35	7	16	0.00026	0.241
33	35	8	1	0.00013	0.129
33	35	8	4	0.00013	0.129
33	35	8	11	0.00026	0.241
33	35	8	12	0.00013	0.129
33	35	8	15	0.00013	0.129
33	37	4	11	0.00013	0.129
33	37	6	11	0.00013	0.129

33	37	6	12	0.00013	0.129
33	37	6	16	0.00013	0.129
33	37	7	12	0.00013	0.129
33	37	7	16	0.00013	0.129
33	38	8	1	0.00013	0.129
33	38	8	3	0.00013	0.129
33	38	8	4	0.00013	0.129
33	38	8	13	0.00013	0.129
33	38	12	1	0.00013	0.129
33	38	12	3	0.00013	0.129
33	38	12	4	0.00013	0.129
33	38	12	13	0.00013	0.129
33	41	8	3	0.00013	0.129
33	41	17	3	0.00013	0.129
33	44	5	1	0.00013	0.129
33	44	5	16	0.00013	0.129
33	44	8	1	0.00013	0.129
33	44	8	16	0.00013	0.129
33	45	6	1	0.00013	0.129
33	45	6	4	0.00026	0.241
33	45	6	11	0.00013	0.129
33	45	8	1	0.00013	0.129
33	45	8	4	0.00026	0.241
33	45	8	11	0.00013	0.129
33	49	7	3	0.00013	0.129
33	49	7	4	0.00013	0.129
33	49	8	3	0.00013	0.129
33	49	8	4	0.00013	0.129
33	51	2	1	0.00026	0.241
33	51	2	7	0.00013	0.129
33	51	2	16	0.00013	0.129
33	51	8	1	0.00026	0.241
33	51	8	7	0.00013	0.129
33	51	8	13	0.00013	0.129
33	51	8	16	0.00026	0.241
33	51	14	13	0.00013	0.129
33	51	14	16	0.00013	0.129
33	52	8	1	0.00013	0.129
33	52	8	16	0.00013	0.129
33	52	12	1	0.00013	0.129
33	52	12	16	0.00013	0.129
33	55	3	3	0.00013	0.129
33	55	3	4	0.00013	0.129
33	55	3	11	0.00013	0.129
33	55	3	13	0.00013	0.129
33	55	3	16	0.00026	0.241
33	55	7	13	0.00013	0.129
33	55	7	16	0.00013	0.129

33	55	8	3	0.00013	0.129
33	55	8	4	0.00013	0.129
33	55	8	11	0.00013	0.129
33	55	8	16	0.00013	0.129
33	57	6	1	0.00026	0.241
33	57	6	3	0.00013	0.129
33	57	6	7	0.00013	0.129
33	57	8	1	0.00026	0.241
33	57	8	3	0.00013	0.129
33	57	8	7	0.00013	0.129
33	58	3	3	0.00013	0.129
33	58	3	11	0.00013	0.129
33	58	4	3	0.00013	0.129
33	58	4	16	0.00013	0.129
33	58	7	1	0.00026	0.241
33	58	7	3	0.00013	0.129
33	58	7	7	0.00013	0.129
33	58	7	16	0.00053	0.453
33	58	8	1	0.00026	0.241
33	58	8	3	0.00013	0.129
33	58	8	7	0.00013	0.129
33	58	8	11	0.00013	0.129
33	58	8	16	0.00039	0.345
33	78	5	1	0.00013	0.129
33	78	5	3	0.00013	0.129
33	78	16	1	0.00013	0.129
33	78	16	3	0.00013	0.129
66	15	7	7	0.00013	0.129
66	15	7	11	0.00013	0.129
66	15	12	7	0.00013	0.129
66	15	12	11	0.00013	0.129
66	18	7	7	0.00013	0.129
66	18	7	11	0.00013	0.129
66	18	12	7	0.00013	0.129
66	18	12	11	0.00013	0.129
68	7	7	4	0.00013	0.129
68	7	7	16	0.00013	0.129
68	13	5	4	0.00013	0.129
68	13	5	11	0.00013	0.129
68	13	7	4	0.00013	0.129
68	13	7	11	0.00013	0.129
68	14	5	1	0.00013	0.129
68	14	5	3	0.00013	0.129
68	14	7	3	0.00013	0.129
68	14	7	16	0.00013	0.129
68	14	8	1	0.00039	0.345
68	14	8	3	0.00039	0.345
68	14	8	7	0.00013	0.129

68	14	8	13	0.00013	0.129
68	14	8	15	0.00013	0.129
68	14	8	16	0.00013	0.129
68	14	12	3	0.00013	0.129
68	14	12	15	0.00013	0.129
68	14	17	1	0.00013	0.129
68	14	17	13	0.00013	0.129
68	18	4	11	0.00013	0.129
68	18	4	13	0.00013	0.129
68	18	5	1	0.00013	0.129
68	18	5	3	0.00066	0.549
68	18	5	4	0.00013	0.129
68	18	5	15	0.00013	0.129
68	18	5	16	0.00013	0.129
68	18	6	3	0.00013	0.129
68	18	7	3	0.00039	0.345
68	18	7	4	0.00013	0.129
68	18	7	16	0.00026	0.241
68	18	8	1	0.00013	0.129
68	18	8	3	0.00026	0.241
68	18	8	16	0.00013	0.129
68	18	12	3	0.00013	0.129
68	18	12	11	0.00013	0.129
68	18	12	13	0.00013	0.129
68	18	12	15	0.00013	0.129
68	35	4	1	0.00013	0.129
68	35	4	4	0.00013	0.129
68	35	4	7	0.00013	0.129
68	35	4	11	0.00013	0.129

68	35	4	16	0.00013	0.129
68	35	14	7	0.00013	0.129
68	35	14	11	0.00013	0.129
68	35	15	1	0.00013	0.129
68	35	15	4	0.00013	0.129
68	35	15	16	0.00013	0.129
68	38	3	7	0.00013	0.129
68	38	3	8	0.00013	0.129
68	38	12	7	0.00013	0.129
68	38	12	8	0.00013	0.129
68	41	8	1	0.00013	0.129
68	41	8	13	0.00013	0.129
68	41	17	1	0.00013	0.129
68	41	17	13	0.00013	0.129
68	45	5	3	0.00013	0.129
68	45	6	3	0.00013	0.129
68	49	1	8	0.00013	0.129
68	49	1	16	0.00013	0.129
68	49	4	1	0.00013	0.129
68	49	4	13	0.00013	0.129
68	49	5	3	0.00013	0.129
68	49	5	4	0.00026	0.241
68	49	5	11	0.00013	0.129
68	49	7	1	0.00013	0.129
68	49	7	3	0.00013	0.129
68	49	7	4	0.00039	0.345
68	49	7	8	0.00013	0.129
68	49	7	11	0.00013	0.129
68	49	7	13	0.00013	0.129

68	49	7	16	0.00026	0.241
68	51	4	1	0.00013	0.129
68	51	4	4	0.00013	0.129
68	51	4	7	0.00013	0.129
68	51	4	11	0.00013	0.129
68	51	4	16	0.00013	0.129
68	51	7	1	0.00013	0.129
68	51	7	16	0.00013	0.129
68	51	14	7	0.00013	0.129
68	51	14	11	0.00013	0.129
68	51	15	1	0.00026	0.241
68	51	15	4	0.00013	0.129
68	51	15	16	0.00026	0.241
68	52	5	3	0.00013	0.129
68	52	5	15	0.00013	0.129
68	52	8	3	0.00013	0.129
68	52	8	15	0.00013	0.129
68	52	12	3	0.00026	0.241
68	52	12	15	0.00026	0.241
68	53	4	1	0.00013	0.129
68	53	4	11	0.00013	0.129
68	53	4	13	0.00026	0.241
68	53	7	1	0.00013	0.129
68	53	7	13	0.00013	0.129
68	53	12	11	0.00013	0.129
68	53	12	13	0.00013	0.129
68	55	3	7	0.00013	0.129
68	55	3	8	0.00013	0.129
68	55	12	7	0.00013	0.129

68	55	12	8	0.00013	0.129
68	56	1	8	0.00013	0.129
68	56	1	16	0.00013	0.129
68	56	7	8	0.00013	0.129
68	56	7	16	0.00013	0.129
68	58	5	3	0.00013	0.129
68	58	5	16	0.00013	0.129
68	58	7	1	0.00013	0.129
68	58	7	3	0.00013	0.129
68	58	7	16	0.00026	0.241
68	58	15	1	0.00013	0.129
68	58	15	16	0.00013	0.129
69	18	1	11	0.00026	0.241
69	18	7	11	0.00013	0.129
69	18	12	11	0.00013	0.129
69	35	1	4	0.00013	0.129
69	35	1	11	0.00013	0.129
69	35	3	8	0.00013	0.129
69	35	3	11	0.00013	0.129
69	35	4	1	0.00026	0.241
69	35	4	4	0.00026	0.241
69	35	4	8	0.00013	0.129
69	35	4	11	0.00013	0.129
69	35	12	1	0.00013	0.129
69	35	12	4	0.00026	0.241
69	35	12	11	0.00013	0.129
69	52	1	3	0.00013	0.129
69	52	1	11	0.00013	0.129
69	52	5	3	0.00013	0.129

69	52	5	11	0.00013	0.129
69	55	1	3	0.00026	0.241
69	55	1	4	0.00013	0.129
69	55	1	11	0.00079	0.643
69	55	1	16	0.00013	0.129
69	55	3	3	0.00013	0.129
69	55	3	11	0.00013	0.129
69	55	5	3	0.00013	0.129
69	55	5	11	0.00013	0.129
69	55	7	11	0.00026	0.241
69	55	7	16	0.00013	0.129
69	55	12	4	0.00013	0.129
69	55	12	11	0.00026	0.241
69	58	1	3	0.00013	0.129
69	58	1	11	0.00026	0.241
69	58	1	16	0.00013	0.129
69	58	3	3	0.00013	0.129
69	58	3	8	0.00013	0.129
69	58	3	11	0.00026	0.241
69	58	4	8	0.00013	0.129
69	58	4	11	0.00013	0.129
69	58	7	11	0.00013	0.129
69	58	7	16	0.00013	0.129
74	14	3	3	0.00013	0.129
74	14	3	11	0.00013	0.129
74	14	8	3	0.00013	0.129
74	14	8	11	0.00013	0.129
74	15	2	10	0.00039	0.345
74	15	2	11	0.00013	0.129

74	15	2	14	0.00013	0.129
74	15	2	16	0.00013	0.129
74	15	4	10	0.00026	0.241
74	15	4	11	0.00013	0.129
74	15	4	14	0.00013	0.129
74	15	7	10	0.00013	0.129
74	15	7	16	0.00013	0.129
74	35	2	10	0.00026	0.241
74	35	2	11	0.00013	0.129
74	35	2	14	0.00013	0.129
74	35	4	10	0.00026	0.241
74	35	4	11	0.00013	0.129
74	35	4	14	0.00013	0.129
74	49	2	11	0.00013	0.129
74	49	7	11	0.00013	0.129
74	51	2	11	0.00013	0.129
74	51	7	11	0.00013	0.129
74	58	2	10	0.00013	0.129
74	58	2	16	0.00013	0.129
74	58	3	3	0.00013	0.129
74	58	3	11	0.00013	0.129
74	58	7	10	0.00013	0.129
74	58	7	16	0.00013	0.129
74	58	8	3	0.00013	0.129
74	58	8	11	0.00013	0.129
