# Supplementary material for: Entropy of human leukocyte antigen and killer-cell immunoglobulin-like receptor systems in immune-mediated disorders: A pilot study on multiple sclerosis
Source: PLoS One. 2019 Dec 17;14(12):e0226615. doi: 10.1371/journal.pone.0226615 (PMC6917289; doi:10.1371/journal.pone.0226615)
Supplement: S4 Table — (PDF) [file pone.0226615.s009.pdf]

S4 Table. Linkage Disequilibrium

The following Table shows the observed and expected frequencies of the HLA four-loci haplotype HLA-A*30, B*18, C*05, DR*03 in RRMS patients and controls. This haplotype has the highest frequency in the Sardinian population. The Linkage Disequilibrium (LD) and the normalized LD are expressed by the parameters *D* and *D′*, respectively.

No statistically significant differences for LD were observed between the two groups of patients and controls. All corrected P_c values – which were calculated by multiplying the P values obtained with Fisher's exact test by the number of tested HLA haplotypes – were greater than 0.05.

HLA Haplotypes	Sardinian healthy controls (619 controls)				RRMS patients (181 patients)			
	Observed n (%)	Expected n (%)	*D* (%)	*D′*	Observed n (%)	Expected n (%)	*D* (%)	*D′*
**Complete HLA haplotype**								
HLA-A*30, B*18, C*05, DR*03	154 (12.44)	99 (8.04)	4.40	1	78 (20.63)	63 (16.59)	4.04	1
**Partial HLA haplotypes**								
HLA-A*30, B*18, C*05	171 (13.81)	95 (7.71)	6.10	0.89	84 (22.22)	63 (16.79)	5.43	1
HLA-A*30, B*18, DR*03	154 (12.44)	90 (7.24)	5.20	0.93	80 (21.16)	63 (16.75)	4.41	0.89
HLA-A*30, C*05, DR*03	155 (12.52)	85 (6.89)	5.63	0.91	78 (20.63)	59 (15.56)	5.07	0.60
HLA-B*18, C*05, DR*03	188 (15.19)	108 (8.70)	6.49	0.86	98 (25.93)	74 (19.61)	6.32	1
HLA-A*30, B*18	174 (14.05)	59 (4.73)	9.32	0.67	89 (23.54)	40 (10.60)	12.94	0.71
HLA-A30, C*05	175 (14.14)	45 (3.65)	10.49	0.70	84 (22.22)	32 (8.39)	13.83	0.68
HLA-A*30, DR*03	158 (12.76)	51 (4.08)	8.68	0.60	82 (21.69)	35 (9.38)	12.31	0.63
HLA-B*18, C*05	214 (17.29)	62 (4.99)	12.30	0.84	104 (27.51)	40 (10.70)	16.81	0.91
HLA-B*18, DR*03	199 (16.07)	69 (5.59)	10.48	0.64	105 (27.78)	45 (11.97)	15.81	0.77
HLA-C*05, DR*03	192 (15.51)	53 (4.31)	11.20	0.73	98 (25.93)	36 (9.47)	16.46	0.84
**Single HLA alleles**								
HLA-A*30	230 (18.58)				109 (28.84)			
HLA-B*18	315 (25.44)				139 (36.77)			
HLA-C*05	243 (19.63)				110 (29.10)			
HLA-DR*03	272 (21.97)				123 (32.54)			

An analogous result was found for the second most frequent HLA four-loci haplotype HLA-A*02, B*58, C*07, DR*16 in the Sardinian population, as shown in the following Table. No statistically significant differences for LD were observed between controls and RRMS patients.

HLA Haplotypes	Sardinian healthy controls (619 controls)				RRMS patients (181 patients)			
	**Observed n (%)**	**Expected n (%)**	***D* (%)**	***D′***	**Observed n (%)**	**Expected n (%)**	***D* (%)**	***D′***
**Complete HLA haplotype**								
HLA-A*02, B*58, C*07, DR*16	82 (6.62)	57 (4.57)	2.05	1	10 (2.65)	6 (1.58)	1.07	1
**Partial HLA haplotypes**								
HLA-A*02, B*58, C*07	107 (8.64)	71 (5.73)	2.91	0.71	17 (4.50)	9 (2.39)	2.11	0.50
HLA-A*02, B*58, DR*16	82 (6.62)	51 (4.10)	2.52	0.38	10 (2.65)	5 (1.43)	1.22	0.60
HLA-A*02, C*07, DR*16	106 (8.56)	86 (6.95)	1.61	0.38	16 (4.23)	11 (3.01)	1.22	0.34
HLA-B*58, C*07, DR*16	101 (8.16)	58 (4.69)	3.47	0.86	12 (3.17)	5 (1.43)	1.74	1
HLA-A*02, B*58	109 (8.80)	41 (3.34)	5.46	0.68	19 (5.03)	6 (1.58)	3.45	0.72
HLA-A30, C*07	209 (16.88)	108 (8.74)	8.14	0.40	38 (10.05)	22 (5.79)	4.26	0.24
HLA-A*02, DR*16	138 (11.15)	70 (5.68)	5.47	0.40	25 (6.61)	11 (2.89)	3.72	0.43
HLA-B*58, C*07	134 (10.82)	42 (3.39)	7.43	0.93	20 (5.29)	6 (1.48)	3.81	0.78
HLA-B*58, DR*16	102 (8.24)	27 (2.21)	6.03	0.66	12 (3.17)	3 (0.74)	2.43	0.43
HLA-C*07, DR*16	158 (12.76)	72 (5.78)	6.98	0.51	25 (6.61)	10 (2.71)	3.90	0.44
**Single HLA alleles**								
HLA-A*02	363 (29.32)				94 (24.87)			
HLA-B*58	141 (11.39)				24 (6.35)			
HLA-C*07	369 (29.81)				88 (23.28)			
HLA-DR*16	240 (19.39)				44 (11.64)			

The expected frequencies for two-locus haplotypes were obtained by multiplying the frequencies of the component alleles, as proposed by Lewontin[1] and discussed by Contu[2] *et al.*

For three- and four-locus haplotypes the expected frequencies were obtained by applying the formulas proposed by Thomson and Baur[3] and Slatkin[4], respectively.

The Linkage Disequilibrium is expressed by the parameter *D* which corresponds to the difference between the observed and expected frequencies. *D′* is the parameter *D* normalized to one ($-1 \leq D' \leq 1$) which was calculated by applying the normalization formulas proposed by Lewontin, Thomson and Baur and Slatkin for two-, three- and four-locus haplotypes, respectively, as summarized by Robinson[5]*et al.*

[1]Lewontin RC. The interaction of selection and linkage. General considerations: heterotic models. Genetics 1964; 49: 49-67.

[2]Contu L, Arras M, Carcassi C, La Nasa G and Mulargia M. HLA structure of the Sardinian population: a haplotype study of 551 families. Tissue Antigens 1992; 40: 165-174.

[3]Thomson G and Baur M. Third order linkage disequilibrium. Tissue Antigens 1984; 24: 250-255.

[4]Slatkin M. On treating the chromosome as the unit of selection. Genetics 1972; 72: 157-168.

[5]Robinson WP, Asmussen MA and Thomson G. Three-locus systems impose additional constraints on pairwise disequilibria. Genetics 1991; 129: 925-930.

If p_A, p_B, p_C, p_D are the frequencies of the HLA-A, -B, -C and -DR alleles and D_{AB}, D_{ABC}, etc. are the Linkage Disequilibrium values for the AB, ABC, etc. haplotypes, the expected frequencies f_{exp} for two-, three- and four-loci haplotypes are given by:

$$f_{exp}(AB) = p_A p_B$$

$$f_{exp}(ABC) = p_A p_B p_C + p_A D_{BC} + p_B D_{AC} + p_C D_{AB}$$

$$f_{exp}(ABCD) = p_A p_B p_C p_D + p_A D_{BCD} + p_B D_{ACD} + p_C D_{ABD} + p_D D_{ABC} + p_A p_B D_{CD} + p_A p_C D_{BD} + p_A p_D D_{BC} + p_B p_C D_{AD} + p_B p_D D_{AC} + p_C p_D D_{AB}$$
