# Supplementary figures and images for: Entropy of human leukocyte antigen and killer-cell immunoglobulin-like receptor systems in immune-mediated disorders: A pilot study on multiple sclerosis
Source: PLoS One. 2019 Dec 17;14(12):e0226615. doi: 10.1371/journal.pone.0226615 (PMC6917289; doi:10.1371/journal.pone.0226615)

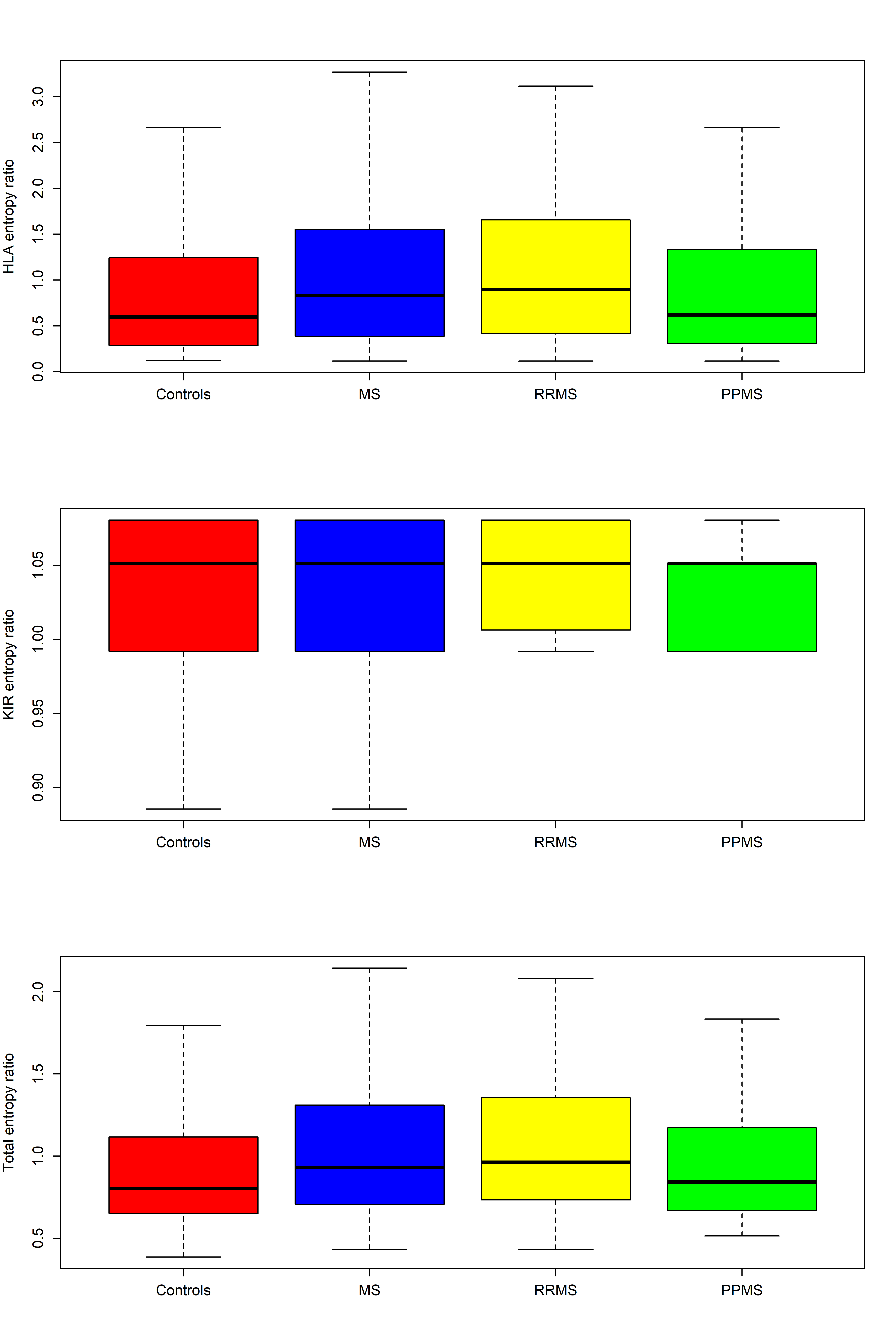

Supplement: S1 Fig — The following boxplots represent Shannon’s entropy associated to HLA-A, -B, -C, -DR haplotypes and couples of inhibitory KIR genes in a cohort of 270 patients affected by multiple sclerosis (MS), stratified into a group of 81 patients with primary progressive multiple sclerosis (PPMS) and a group of 189 patients with relapsing remitting multiple sclerosis (RRMS). The HLA, KIR and total entropies for each group of patients were compared to the respective entropies of a group of 619 healthy controls. In the box and whisker plots, the whiskers represent the extremities of the interquartile ranges (IQR), i.e. the lower and upper quartiles, while the median of each sample is marked as a bold line. The three boxplots clearly show that the median of the HLA and total entropy is higher in patients affected by RRMS in comparison to healthy controls and patients affected by PPMS. (TIFF) [file pone.0226615.s010.tiff]
